# *α*-Glucosidase inhibitive diarylheptanoids from *Ottelia acuminata* var. *acuminata*, a traditional vegetable of Bai Nationality in Yunnan

**DOI:** 10.1007/s13659-022-00341-4

**Published:** 2022-06-10

**Authors:** Hong-Xing Liu, Jun-Zeng Ma, Yan-Song Ye, Jian-Jun Zhao, Shi-Jie Wan, Xin-Yue Hu, Gang Xu

**Affiliations:** 1 State Key Laboratory of Phytochemistry and Plant Resources in West China and Yunnan Key Laboratory of Natural Medicinal Chemistry, Kunming Institute of Botany, Chinese Academy of Sciences, Kunming, 650201 China; 2 University of Chinese Academy of Sciences, Beijing, 100049 China

**Keywords:** *Ottelia acuminata* var. *acuminata*, Bai nationality, Vegetable, Diarylheptanoids, *α*-glucosidase

## Abstract

Diabetes is an urgent health issue characterized by ethnic and regional variations, and is inseparable from the different dietary habits. It is worthy to note that the incidence of diabetes in Bai nationality has been reported to be much lower than Han in China. As a daily vegetable of Bai, the phytochemical and antidiabetic study of *Ottelia* *acuminata* var. *acuminata* had not been carried out. In this study, 41 metabolites with diverse diarylheptanoid (six new ones, Otteacumienes A–F), flavone, sesquiterpenoid, coumarin, lignan, polyacetylene, and alkaloid skeletons were characterized from *O. acuminata* var. *acuminata*. Among them, the racemic nature of **3** was characterized by chiral resolution and calculated ECD methods. The biological study revealed diarylheptanoids showed significant *α*-glucosidase inhibitory activities with **5** as the most effective one (60-fold stronger than acarbose). Molecular docking studies indicated that these structures have different binding cavities with acarbose. This study demonstrated that *O. acuminata* var. *acuminata* might correlated with the low incidence diabetes of Bai and the diarylheptanoids may have potential therapeutic value for diabetes mellitus.

**Graphical Abstract:**

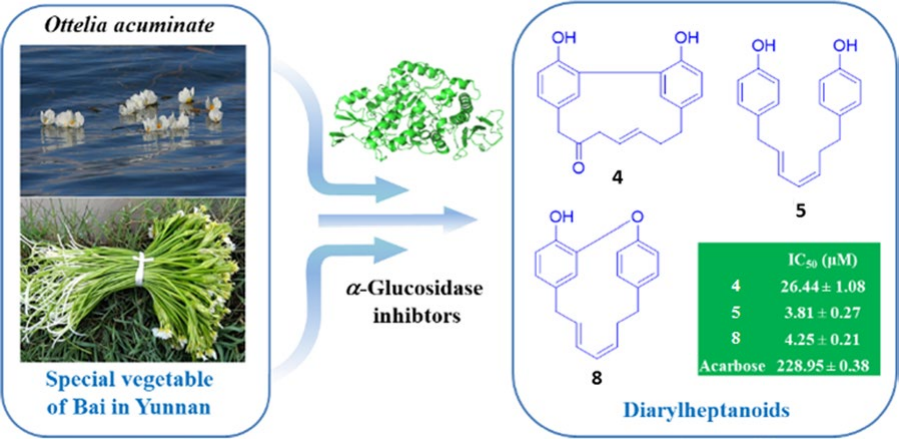

**Supplementary Information:**

The online version contains supplementary material available at 10.1007/s13659-022-00341-4.

## Introduction

The latest research from IDF (International Diabetes Federation) revealed that about 8.8% of the world’s population suffers from diabetes disease. Among them, type 2 diabetes (T2DM) is the most common type, accounting for more than 90% of all diabetic cases worldwide [1]. Treatment of T2DM requires both diet and exercise to address the overweight or obesity. A range of combination therapy options were available for T2DM such as sulfonylureas, *α*-glucosidase inhibitors (AGI), thiazolidinediones, dipeptidyl peptidase 4 (DPP-4) inhibitors, glucagon-like peptide 1 (GLP-1) agonists, and sodium-glucose co-transporter 2 (SGLT-2) inhibitors [2]. Among them, *α*-glucosidase is one of the most important digestive enzymes in the human body involved in the final step of carbohydrate digestion, and its inhibitors contributes to control postprandial glucose for diabetes treatment [3]. However, the common side effects of flatulence, diarrhea, and hepatotoxicity for the clinical *α-*glucosidase inhibitors (including acarbose and miglitol) encouraged us to find new type of inhibitors with high safety and efficiency [4].

It’s well-know that the development of diabetes is closely relevant to the dietary and lifestyle. Interestingly, the investigation of the incidence of diabetes in China has showed the characteristics of ethnic and regional variations [5], especially for the Bai nationality (7.83%) in contrast to the Han nationality (11.83%) in rural Yunnan [6]. As one of the major ethnic minorities in Yunnan, the Bai nationality has a long history and splendid culture, most of whom live in Dali Bai Autonomous Prefecture [7]. Notably, *O. acuminata* var. *acuminata* had long been used as a traditional daily vegetable in Bai as record in the “Textual Research on Reality and Titles of Plants” published in 175 years ago [8].

As an edible unique plant in China, *Ottelia acuminata* var. *acuminata* belongs to the genus *Ottelia* of the Hydrocharitaceae family, and mainly distributed in Yunnan Province [9]. This plant is also a famous aquatic ornamental plant, attracting millions of tourists to Erhai and Lugu Lakes every year during the blooming period from May to October. Additionally, the high requirements for water quality could be applied to monitor water pollution and environmental protection [10]. In order to explore antidiabetic chemical constituents of a daily vegetable for Bai nationality, *O. acuminata* var. *acuminata* was selected for this study. However, except for a preliminary analytical investigation by HPLC–ESI–MS, the phytochemical and antidiabetic ingredients of this vegetable have not been reported so far [11].

**Fig. 1:**
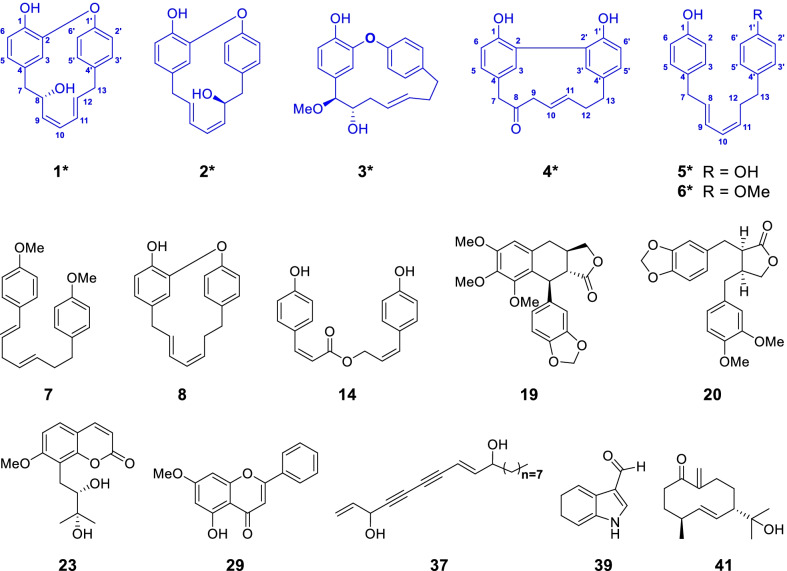
Structures of new diarylheptanoids (**1**–**6**) and other representative compounds

In this study, the first study of phytochemistry on *O. acuminata* var. *acuminata* was conducted and 41 compounds including six new diarylheptanoids (Otteacumienes A-F, **1**–**6**) and 35 known ones possessing diverse diarylheptanoid, flavone, sesquiterpenoid, coumarin, lignan, and polyacetylene skeletons (**7**–**41**) were obtained (Fig. 1). Structurally, the six new diarylheptanoids could be classified into three different types: diarylether, biaryl, and linear types of diaryheptanoids. Being distinct from diarylheptanoids reported from families Officinarum, Katsumadai, Blepharocalyx, Zingiberaceae, and Betulaceae [12], these structures in this study are characterized by low degree of oxidation. Bioactive study of compounds **3**–**8** showed substantial inhibitory effects on *α*-glucosidase but negligible effects on PTP1B, suggesting that they might be selective inhibitors of *α*-glucosidase. In addition, the molecular docking studies implied that different types diarylheptanoids are binding to the different sites of *α*-glucosidase, and the phenolic hydroxyl groups on diaryheptanoids might play a key role for their inhibitory activity.

## Result and discussion

The DCM fraction of the EtOAc extract of *O*. *acuminata* var. *acuminata* was subjected to MCI-gel column, silica gel column chromatography, RP-C_18_ column chromatography, and preparative HPLC to afford eight diarylheptanoids including six new diarylheptanoids (Otteacumienes A–F, **1**–**6**) and 35 known compounds possessing different phenylpropionids, coumarins, lignins, flavonoids, polyacetylene, sesquiterpenoid, and alkaloid architectures. Their structures were elucidated by comprehensive methods including NMR, MS, X-ray diffraction analyses, and calculated ECD spectra. In the antidiabetic studies, the diarylheptanoids showed significant *α-*glucosidase inhibitory activities and negligible effects on PTP1B. Additionally, molecular docking study further illustrated the possible binding cavities of the diarylheptanoids.

### Structural identification of compounds

Otteacumiene A (**1**) was isolated as a yellow crystal and its molecular formula was determined as C_19_H_18_O_3_ by the HRESIMS at *m/z* 293.1182 [M–H]^–^ (calculated for 293.1178). The IR spectrum showed characteristic hydroxyl broad absorption band at 3442 cm^–1^ and aromatic ring absorption band at 1632, 1590, 1519, 1428 cm^–1^. The ^1^H-NMR signals at *δ*_H_ 5.36 (1H, d, *J* = 2.2 Hz, H-3), 6.57 (1H, dd, *J* = 8.0, 2.2 Hz, H-5), 6.68 (1H, d, *J* = 8.0 Hz, H-6), *δ*_H_ 7.23 (1H, dd, *J* = 8.2, 2.6 Hz, H-2′), 7.29 (1H, dd, *J* = 8.2, 2.3 Hz, H-3′), 7.34 (1H, dd, *J* = 8.3, 2.3 Hz, H-5′), and 7.05 (1H, dd, *J* = 8.3, 2.6 Hz, H-6′) suggested a 1,2,4-trisubstituted benzene of an ABX spin system and a 1′,4′-disubstituted aromatic rings of an AA′BB′ spin system (Table 1), respectively [13]. In addition, a *cis* and a *trans* carbon–carbon double bonds were evidenced by key ^1^H NMR data of *δ*_H_ 5.40 (1H, t, *J* = 10.1 Hz, H-9), 5.90 (1H, t, *J* = 10.1 Hz, H-10), 5.33 (1H, m, H-11), and 6.07 (1H, dt, *J* = 15.0, 4.0 Hz, H-12). Besides 16 carbon atoms of two benzene ring and two carbon–carbon double bonds, the remaining three carbon atoms were attributed to be two methylene (C-7 and C-13) and an oxygenated methine (C-8) according to the ^13^C-NMR and DEPT spectra (Table 2). The above characteristic signals implied that **1** could be a diaryheptanoid derivatives [14].

**Table 1:** ^1^H NMR data of compounds **1**–**6**^a^

No.	**1**^b^	**2**^b^	**3**^b^	**4**^b^	**5**^c^	**6**^c^
2					6.75, m	6.76, m
3	5.36, d (2.2)	5.33, d (2.1)	5.77, d (1.9)	6.87, d (2.5)	7.00, m	7.00, m
5	6.57, dd (8.0, 2.2)	6.51, dd (8.3, 2.1)	6.71, dd (8.2, 1.9)	7.03, dd (8.4, 2.5)	7.00, m	7.00, m
6	6.68, d (8.0)	6.65, d (8.3)	6.81, d (8.2)	6.85, d (8.4)	6.75, m	6.76, m
7	2.34, d (4.5)	2.96, m	3.92, d (7.5)	3.79, m	3.31, d (7.0)	3.31, d (7.0)
8	4.24, m	5.52, m	3.42, dt (7.5, 2.5)		5.74, dt (15.0, 7.0)	5.74, dt (14.4, 7.0)
9	5.40, t (10.1)	5.69, dd (15.6, 11.0)	1.95, m	3.48, d (6.4)	6.38, m	6.36, m
			1.55, m			
10	5.90, t (10.1)	5.96, t (11.2)	4.76, ddd, (15.3, 7.6, 4.4)	5.54, dt (14.5, 6.4)	5.96, t (11.0)	5.96, t (11.0)
11	5.33, m	5.23, t (11.2)	5.11, ddd, (15.3, 9.3, 5.9)	5.79, dt (14.5, 6.9)	5.35, dt (11.0, 7.5)	5.35, dt (11.0, 7.5)
12	6.07, dt (15.0, 4.0)	4.46, td (10.3, 3.2)	2.36, m	2.41, m	2.42, m	2.44, m
			2.04, m			
13	3.51, m	3.15, dd (12.0, 3.2)	2.87, dt, (12.7, 5.0)	2.76, t (7.3, 5.8)	2.58, m	2.61, t (7.3)
		2.68, t (12.0)	2.62, ddd, (12.7, 10.3, 4.3)			
2ʹ	7.23, dd (8.2, 2.6)	7.05, dd (8.4, 2.6)	6.79, dd (8.2, 1.5)		6.74, m	6.84, m
3ʹ	7.29, dd (8.2, 2.3)	7.42, dd (8.4, 2.3)	6.93, dd (8.2, 1.2)	7.28, d (2.4)	7.04, m	7.13, m
5ʹ	7.34, dd (8.3, 2.3)	7.10, dd (8.2, 2.3)	7.14, overlap	6.98, dd (8.3, 2.4)	7.04, m	7.13, m
6ʹ	7.05, dd (8.3, 2.6)	6.83, dd (8.2, 2.6)	7.14, overlap	6.72, d (8.3)	6.74, m	6.84, m
1-OH					8.16, s	8.17, s
1-OCH_3_						
7-OCH_3_			3.20, s			
1ʹ-OH					8,12, s	
1ʹ-OCH_3_						3.75, s

^a^*δ* in parts per million, *J* in Hz, and obtained at 600 MHz

^b^The solvent was CD_3_OD

^c^The solvent was CD_3_COCD_3_

**Table 2:** ^13^C NMR and DEPT (150 MHz) data of **1**–**6**^a^

No.	**1**^b^	**2**^b^	**3**^b^	**4**^b^	**5**^c^	**6**^c^
1	145.0, s	144.6, s	147.1, s	152.9, s	156.5, s	156.5, s
2	152.3, s	151.6, s	151.5, s	128.3, s	116.0, d	116.0, d
3	118.0, d	118.0, d	120.1, d	135.5, d	130.3, d	130.3, d
4	134.1, s	131.3, s	130.6, s	128.7, s	131.8, s	131.8, s
5	122.5, d	121.7, d	120.9, d	129.8, d	130.3, d	130.3, d
6	116.4, d	116.5, d	117.2, d	117.7, d	116.0, d	116.0, d
7	45.7, t	36.9, t	87.5, d	50.2, t	38.8, t	38.8, t
8	74.9, d	135.6, d	75.0, d	212.5, s	134.6, d	134.6, d
9	135.4, d	129.3, d	35.2, t	47.8, t	127.0, d	127.0, d
10	126.7, d	131.5, d	128.5, d	126.0, d	129.7, d	129.8, d
11	128.2, d	131.6, d	131.5, d	137.5, d	130.2, d	130.0, d
12	136.7, d	72.2, d	37.1, t	35.7, t	30.6, t	30.6, t
13	38.3, t	45.2, t	35.9, t	33.7, t	35.6, t	35.6, t
1′	157.6, s	157.2, s	158.4, s	152.9, s	156.3, s	158.9, s
2′	126.9, d	125.3, d	123.3, d	126.6, s	115.8, d	114.4, d
3′	132.1, d	131.6, d	133.8, d	137.1, d	130.1, d	130.1, d
4′	138.6, s	136.7, s	140.5, s	134.5, s	133.3, s	134.5, s
5′	134.4, d	133.8, d	130.9, d	130.0, d	130.1, d	130.1, d
6′	124.5, d	124.4, d	124.6, d	116.3, d	115.8, d	114.4, d
7-OCH_3_			57.4, q			
1′-OCH_3_						55.3, q

^a^*δ* in parts per million, *J* in Hz, and obtained at 150 MHz

^b^Solven: CD_3_OD

^c^Solvent: CD_3_COCD_3_

**Fig. 2:**
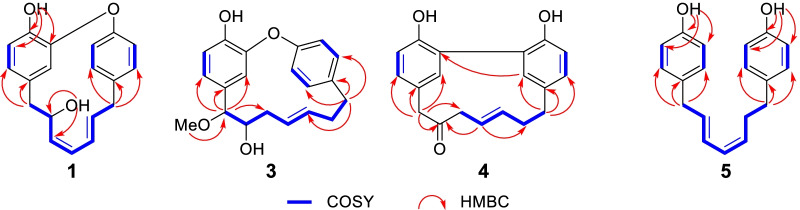
Key ^1^H-^1^H COSY and HMBC correlations of **1**, **3**, **4**, and **5**

**Fig. 3:**
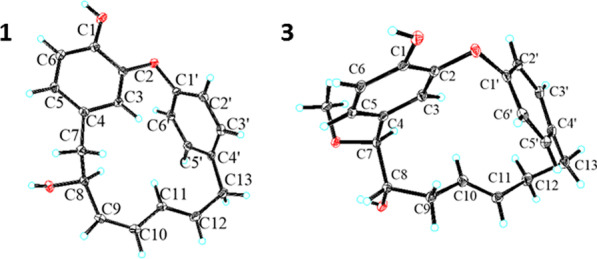
X-ray crystallographic structures for **1** and **3**

In the 2D NMR spectra, the key ^1^H-^1^H COSY correlations of H-7 (*δ*_H_ 2.34, d, *J* = 4.5 Hz)/H-8/H-9/H-10/H-11/H-12/H-13 (*δ*_H_ 3.51, m), together with the obvious HMBC correlations from H_2_-7 to C-3/C-5 and H_2_-13 to C-3′/C-5′/C-11 confirmed the presence of oxygenated unsaturated heptane chain and the connection of two benzene ring. The linkage of C-2 and C-1′ through an oxygen atom was elucidated by the downfield chemical shift of C-1 (*δ*_C_ 145.0), C-1′ (*δ*_C_ 157.6), and C-2 (*δ*_C_ 152.3), the key HMBC correlations from 1-OH to C-1/C-2/C-6, together with the degrees of unsaturation. (Fig. 2). Finally, the absolute configuration of C-8 was undoubtedly determined to be 8*S* by X-ray diffraction analysis using Cu K*α* radiation. [Flack parameter = 0.08(4)] (Fig. 3, CCDC 2156830).

**Fig. 4:**
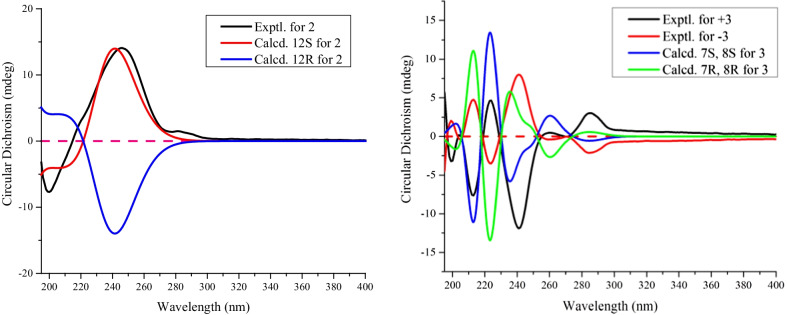
Calculated and experimental ECD spectra of **2** and **3**

Otteacumienes B (**2)** was isolated as yellow oil. The molecular formula was determined to be the same as **1** by HREIMS. Detailed analysis of the ^1^H and ^13^C NMR spectra indicated that the structure of **2** was similar with that of **1** (Tables 1, 2). For **2**, the main difference from **1** was ascribed to the position of carbon–carbon bond (Δ^8,9^ and Δ^10,11^) and hydroxyl (at C-12) in the heptane chain as deduced by the ^1^H-^1^H COSY correlations of H-7 (*δ*_H_ 2.96, m)/H-8 (*δ*_H_ 5.52, m)/H-9 (*δ*_H_ 5.69, dd, *J* = 15.6, 11.0 Hz)/H-10 (*δ*_H_ 5.96, t, *J* = 11.2 Hz)/H-11 (*δ*_H_ 5.23, t, *J* = 11.2 Hz)/H-12 (*δ*_H_ 4.46, td, *J* = 10.3, 3.2 Hz)/H-13 (*δ*_H_ 3.15, dd, *J* = 12.0, 3.2 Hz). The absolute configuration of C-12 was identified as 12*S* by comparison experimental with calculated ECD spectra (Fig. 4).

Otteacumiene C (**3)** was isolated as a colorless needle crystal. Its molecular formula was identified as C_20_H_22_O_4_ by the HRESIMS data at *m/z* 349.1407 [M + Na]^+^ (calcd for 349.1410). Detailed comparison of their 1D NMR data indicated **3** and **1** are structurally similar (Tables 1, 2). The most obvious differences of **3** compared to that of **1** lie in the appearance of an additional methoxy signal at *δ*_H_ 3.20/*δ*_C_ 57.4 as well as the absence of signals for a double bond in the heptane chain. These deductions were confirmed by downfield chemical shifts C-7 (*δ*_C_ 87.5) and C-8 (*δ*_C_ 75.0), the HMBC correlations from H-7 to C-3/C-4/C-5/C-9 and H-13 to C-3′/C-4′/C-5′/C-11, and COSY correlations of H-7/H-8/H-9/H-10/H-11/H-12/H-13 (Fig. 2). In addition, the crystals for single-crystal X-ray diffraction (Fig. 3, CCDC 2155136) were obtained, which clarified the relative configuration and the racemic nature of **3** with the crystal space group *P2/n*. After attempts with various chiral columns and conditions of mobile phase, the chiral separation of **3** was achieved on a chiral-phase HPLC apparatus using a DAICEL CORPORATION semi-preparative column (Fig. S2). To further determine the absolute configurations of enantiomers, quantum-chemical calculation method was used, which eventually assigned the absolute configurations of (+)-**3** and (−)-**3** to be 7*S*, 8*S* and 7*R*, 8*R*, respectively (Fig. 4).

Otteacumiene D (**4**) was also isolated as yellow oil. The molecular formula was established as C_19_H_18_O_3_ from its HRESIMS data at *m/z* 293.1182 [M–H]^−^ (calculated 293.1187). The IR bands at 1508, 1612, 1703, 3390 cm^−1^ suggested the existence of aromatic ring, carbonyl, and hydroxyl groups in the structure. The ^1^H NMR spectrum showed two sets of 1,2,4-trisubstituted benzene rings signals at *δ*_H_ 7.28 (1H, d, *J* = 2.4 Hz, H-3′), 6.98 (1H, dd, *J* = 8.3, 2.4 Hz, H-5′), 6.72 (1H, d, *J* = 8.3 Hz, H-6′) and 6.87 (1H, d, *J* = 2.5 Hz, H-3), 7.03 (1H, dd, *J* = 8.4, 2.5 Hz, H-5), 6.85 (1H, d, *J* = 8.4 Hz, H-6), together with a pair of *trans* double bond signals at *δ*_H_ 5.54 and 5.79 (1H, dt, *J* = 14.5, 6.4 Hz, H-10 and 1H, dt, *J* = 14.5, 6.9 Hz, H-11) (Table 1). The ^13^C NMR and DEPT spectra displayed 19 carbon signals attributing to seven quaternary carbons (one carbonyl), eight methine, and four methylene. The evidences mentioned above, conjugated with a pair of unusual high field aromatic quaternary carbon signals at *δ*_C_ 128.3/ 126.6 (C-2/C-2′) suggest that **4** could be a biaryl type cyclic diaryheptanoid derivative (Tables 1, 2) [13, 14]. The ^1^H-^1^H COSY correlations of H-9 (*δ*_H_ 3.48, d, *J* = 6.4 Hz)/H-10/H-11/H-12 (*δ*_H_ 2.41, m)/H-13 (*δ*_H_ 2.76, *J* = 7.3, 5.8 Hz) and HMBC correlations from H_2_-7 to C-3/C-5/C-9, from H-10 to C-8/C-12, and from H_2_-13 to C-3′/C-4′/C-5′ established its biaryl type cyclic diaryheptanoid architecture. Then, the connection between C-2 and C-2′ was confirmed by the degrees of unsaturation together with the HMBC correlations of H-3/C-2′ and H-3′/C-2 (Fig. 2). Therefore, the structure of **4** was elucidated.

Otteacumiene E (**5**) was obtained as green oil, its molecular formula was determined to be C_19_H_20_O_2_, based on HRESIMS spectrum at *m*/*z* 279.1390 [M–H]^−^ (calculated 279.1385). The ^1^H NMR spectrum revealed the presence of two sets of 1,4-disubstituted benzene rings *δ*_H_ 7.00 (2H, m, H-3, 5), 6.75 (2H, m, H-2, 6), 7.04 (2H, m, H-3′, 5′), 6.74 (2H, m, H-2′, 6′), a pair of *trans* carbon–carbon double bond ( *δ*_H_ 5.74, 1H, dt, *J* = 15.0, 7.0 Hz, H-8 and 6.38, 1H, m, H-9), and a pair of *cis* carbon–carbon double bond (*δ*_H_ 5.96 t, *J* = 11.0, Hz, H-10 and 5.35, 1H, dt, *J* = 11.0, 7.5 Hz, H-11) (Table 1) for a typical linear diaryheptanoid [13, 14]. The ^13^C NMR and DEPT spectroscopic data demonstrated 19 carbon signals attributing to four quaternary carbons, 12 methine, and three methylene (Table 2). In the 2D NMR spectra, the key ^1^H-^1^H COSY correlations of H-7/H-8/H-9/H-10/H-11/H-12H-13 and HMBC correlations from H_2_-7 to C-3/C-5 and H_2_-13 to C-3′/C-5′/C-11 confirmed diaryheptanoid nature of **5**. In addition, the location of two hydroxyl at C-1 and C-1′ was evidenced by the HMBC correlations from 1-OH to C-2/C-6 and from 1′-OH to C-2′/C-6′ (Fig. 2). Therefore, the structure of **5** was established as shown.

Compound **6** was also isolated as green oil, showed a molecular ion at *m*/*z* 293.1547 [M – H]^–^ in the HRESIMS (calculated 293.1542), which correlates to the molecular formula C_20_H_22_O_2_. The ^1^H and ^13^C NMR spectra were similar with those of **5** (Tables 1, 2) except for an extra methoxy signal (*δ*_H_ 3.75, *δ*_C_ 55.3) in **6**. And this methoxyl was deduced to be located at C-1′ by its HMBC correlation with C-1′ (*δ*_C_ 158.9). Therefore, its structure was established as the 1'-*O*-methylated **5** and named otteacumiene F.

By comparing spectroscopic data with literatures, the structures of 35 known compounds were elucidated as (1*E*,4*E*)-1,7-di(4-methoxyphenyl)-1,4-heptadiene (**7**) [15], tedarenes A (**8**) [16], *trans*-cinnamic acid (**9**) [17], *p*-hydroxymethylcinnamate (**10**) [18], *trans*-*p*-hydroxyl ethyl cinnamate (**11**) [19 3-(4-hydroxyphenyl)acrylic acid benzyl ester (**12**) [20], (2*E*)-3-(4-hydroxyphenyl)-2-propenoic acid 2-phenylethyl ester (**13**) [21] (*E*)-cinnamyl-(*E*)-*p*-coumarate (**14**) [22], (*E*)-cinnamyl-(*Z*)-*p*-Coumarate (**15**) [23], (*E*)-cinnamyl-(*E*)-ferulate (**16**) [23], bupleurumin (**17**) [24], marginatoxin (**18**) [25], 3-(3,4-dimethoxybenzyl)-2-(3,4-methylenedioxybenzyl) butyrolactone (**19**) [26], Suchilactone (**20**) [27], osthol (**21**) [28], micropubescin (**22**) [29], 8-(2,3-dihydroxy-3-methylbutyl)-7-methoxy-2*H*-1-benzopyran-2-one (**23**) [29, 30], murraol (**24**) [31] murrayacaurpin B (**25**) [32] 5,6-furanocoumarin (**26**) [33] xanthotoxin (**27**) [34], isopimpinellin (**28**) [35], sakuranetin (**29**) [36] 7,8-dihydroxyflavanone (**30**) [37], 5,3′-dihydroxy-7,4′-dimethoxyflavanone (**31**) [38] pinostrobin (**32**) [39], tectochrysin (**33**) [40] 5,7-dihydroxy-flavone (**34**) [41] desmethylnobiletin (**35**) [42] (9*Z*)-heptadeca-1,9-diene-4,6-diyn-3-one (**36**) [43] (8*E*)-octadeca-1,8-diene-4,6-diyne-3,10-diol (**37**) [43] *1H*-indole-3-carboxylic acid methyl ester (**38**) [44] *1H*-indole-3-carboxaldehyde (**39**) [45] vanillin (**40**) [46] and litseagermacrane (**41**) [47] respectively.

### *α*-Glucosidase and PTB1B inhibitory activity

To explore the antidiabetic chemical constituents from *O. acuminata* var. *acuminata*, the *α*-glucosidase and PTP1B of inhibitory activities of diaryheptanoids **1**–**8** were evaluated. The results revealed that these compounds exhibited different levels of inhibitory activity ranging from 38.29% to 103.55% at 50 μM (Table 3), in which **3**–**8** with inhibition rates more than 50.0% were screened for their IC_50_ values (acarbose as a positive control). Interestingly, all these compounds exhibited more potential inhibitory activity with IC_50_ values of 3.81–26.44 μM (Table 3). Especially, **5** represented most effective inhibitor with 60 times more potent than that of acarbose (228.95 μM), the first-line drug for diabetes treatment. It’s notable that these diaryheptanoids exhibited negligible effects on PTP1B (Table S11), suggesting that these active ingredients may serve as selective inhibitors on *α*-glucosidase.

**Table 3:** Inhibitory effects of 1–8 against *α*-Glucosidase^a^

Compounds	*α*-Glucosidase	
	IC_50_ (μM)^b^	Inhibition ratio (%)^c^
1	–	38.29 ± 1.79
2	–	43.98 ± 0.43
3	4.16 ± 0.27	70.40 ± 2.48
4	26.44 ± 1.08	75.01 ± 2.38
5	3.81 ± 0.27	90.53 ± 2.14
6	8.30 ± 0.29	75.66 ± 1.68
7	4.57 ± 0.23	103.55 ± 1.25
8	4.25 ± 0.21	94.63 ± 0.30
Acarbose^d^	228.95 ± 0.38	20.94 ± 1.91

^a^Data expressed as means ± SD (n = 3)

^b^Inhibition rates than 50.0% were screened for their IC_50_ values

^c^At a concentration of 50 μM

^d^Positive control

### Molecular docking studies

**Fig. 5:**
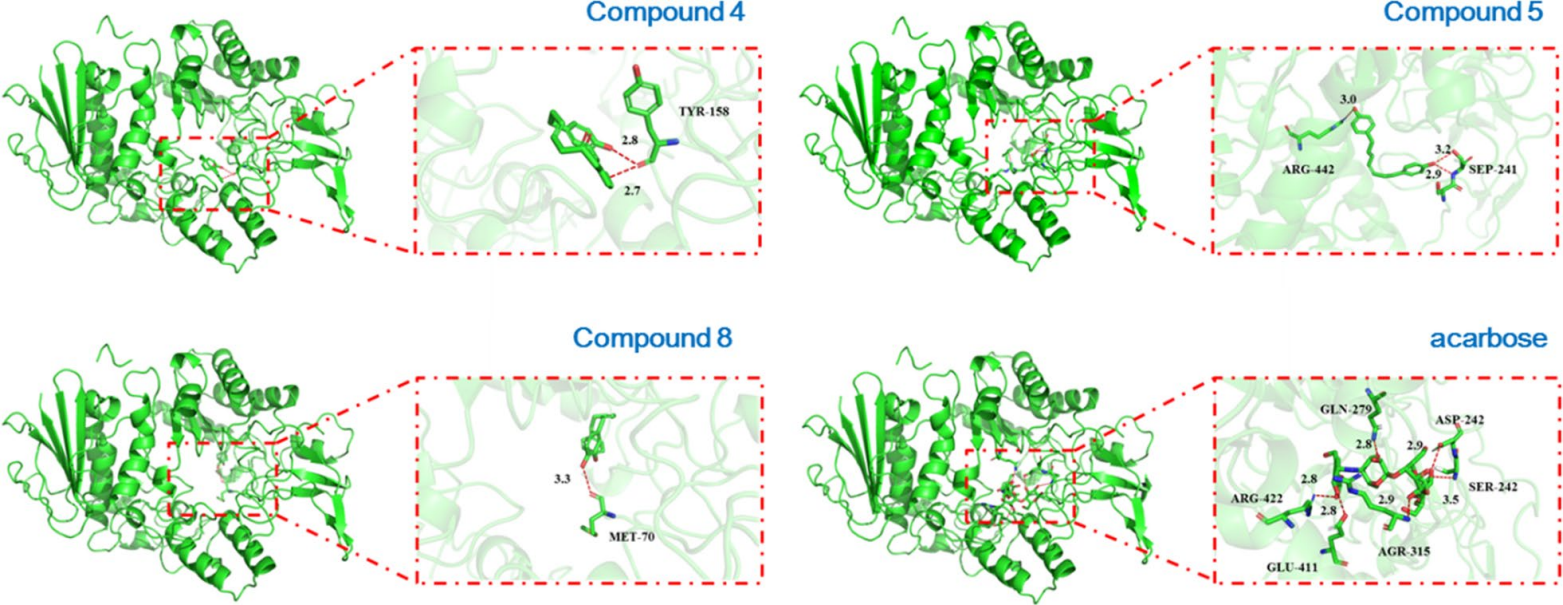
Molecular docking studies of **4**, **5**, **8**, and acarbose against *α*-glucosidase

To further explore the potential antidiabetic mechanism of the diarylheptanoids, the molecular docking studies were performed by using PyMol program. Compounds **4**, **5**, and **8** were selected as representative structures of diarylether, linear, and biaryl types of diaryheptanoids for molecular docking against *α*-glucosidase. The results showed that all the three types of diaryheptanoids act with different cavities mode with that of acarbose [48] which may have contributed to their substantial *α*-glucosidase inhibitory activities. The molecular docking study for acarbose against *α*-glucosidase indicated that this first-line medicine formed six hydrogen bonds with ASP-242 (2.9 Å), SER-242 (3.5 Å), GLN-279 (2.8 Å), ARG-422 (2.8 Å), GLU-411 (2.8 Å), and AGR-315 (2.9 Å), respectively. Notably, the 1′-OH of **5** formed three hydrogen bonds with SEP-241 (2.9 Å, 3.2 Å) and ARG-422 (3.0 Å), which might be the reason for its superior activity in contrast to **4** and **8**. Correspondingly, 1-OH and 1′-OH of **4** formed two hydrogen bonds with TRY-158 (2.7 Å, 2.8 Å) and 1-OH of **8** formed a hydrogen bond with MET-70 (3.3 Å). Although **4**, **5**, and **8** in the docking process tended to combine with the same cavity, the difference in the ability to form hydrogen bonds with amino acid residues might be the reason for their different activities (Fig. 5).

## Conclusion

To the best of our knowledge, this is the first study on the phytochemistry of *O. acuminata* var. *acuminata* and their antidiabetic activity. Totally, 41 metabolites ingcluding eight diarylheptanoids (six new ones), eight phenylpropanoids, four lignans, eight coumarins, seven flavonoids, two polyacetylenes, a sesquiterpenoid, two alkaloids, and a vanillin were characterized in this study. Among them, **3** was obtained as a pair of enantiomers whose absolute configurations were determined by calculated ECD method after chiral separation. The biological activity studies displayed that **3**–**8** exhibited substantial inhibitory activity on *α*-glucosidase as well as negligible effects on PTP1B, which indicated that these diarylheptanoids might be selective inhibitors of *α*-glucosidase. Notably, compound **5** was 60-fold stronger than positive control, acarbose. This study implied a common vegetable of Bai, *O. acuminata* var. *acuminata* may reduce the incidence of diabetes by inhibiting *α*-glucosidase. In addition, this work also provides new lead molecules for antidiabetic disease and a reference for medicinal use of *O. acuminata* var. *acuminata*.

## Experimental

### General experimental procedures

IR spectra were measured on a Bruker FT-IR Tensor-27 infrared spectrophotometer with KBr disks. Optical rotations were recorded on a JASCO P-1020 polarimeter. UV spectra were obtained with a Shimadzu UV-2401PC spectrometer. 1D and 2D NMR spectra were performed on a Bruker DRX-600 spectrometer using TMS as an internal standard. The chemical shifts (*δ*) were expressed in ppm with reference to the solvent signals. HREIMS and HRESIMS analysis were obtained from Waters Xevo TQS and Agilent G6230 TOF mass spectrometers, respectively. Single-crystal X-ray diffraction data were exhibited on a Bruker D8 QUEST diffractometer. Semi-preparative HPLC was performed on a Waters 1525 HPLC with a ZORBAX SB-C_18_ (9.4 × 250 mm) column. Silica gel (100–200 and 200–300 mesh, Qingdao Marine Chemical Co., Ltd., People’s Republic of China), and MCI gel (75–150 μm, Mitsubishi Chemical Corporation, Tokyo, Japan) were used for column chromatography. Fractions were monitored by TLC (GF 254, Qingdao Marine Chemical Co., Ltd.), and spots were visualized by heating silica gel plates immersed in 10% H_2_SO_4_ in ethanol. Methanol (HPLC grade) and acetonitrile (HPLC grade) was purchased from CINC High Purity Solvents Co., Ltd (Shanghai, China). 4-Nitrophenyl-*α*-*d*-glucopyranoside (PNPG), *α*-glucosidase, PTP1B assay kit, quercetin, and acarbose were purchased from Sigma Chemical (Merck KGaA, Darmstadt, Germany).

### Plant materials

The whole plants of *O. acuminata* var. *acuminata* were collected in Dali Prefecture (Yunnan, China) on October 2019. It was identified by Prof. Yun-Heng Ji in Kunming Institute of Botany, Chinese Academy of Sciences. The specimen of this plant was deposited at the State Key Laboratory of Phytochemistry and Plant Resources in West China, Kunming Institute of Botany, Chinese Academy of Sciences, and the voucher number was KIB L-20191001.

### Extraction and isolation

The dried samples of *O. acuminata* var. *acuminata* (20.0 kg) were crushed and extracted four times with methanol for two days each time, and the solvent was recovered under reduced pressure to obtain a crude extract (2.5 kg). The crude extract was eluted with 90% methanol through macroporous resin to obtain 200 g of eluted fractions. The 90% methanol eluent was applied to silica gel column chromatography eluted with dichloromethane (DCM), to afford fraction *O*-DCM (30.5 g). Fraction *O*-DMC (30.5 g) was separated over an MCI-gel column (MeOH-H_2_O from 6:4 to 10:0, v/v) to obtain six fractions from small to large polarities Fr. A–F, which were successively on purified by macroporous resin, silica gel, MCI-gel, RP-C_18_, and preparative or semi-preparative HPLC chromatographic metheds to give the 41 isolates (detailed process, Fig. S1).

***Otteacumiene A (1)****:* Yellow crystals; [*α*] + 52.4 (*c* 0.120, MeOH); UV (MeOH) *λ*_max_ (log *ε*) 205.0 (4.81), 280.8 (3.50) nm; IR (KBr) *ν*_max_ 3442, 1632, 1590, 1519, 1503, 1200, 1109, 1030, 983 cm^–1^; ECD (MeOH) *λ*_max_ (Δ*ε*) 285 (+ 3.75), 271 (– 0.82), 241 (+ 13.52) nm; HRESIMS *m/z* 293.1182 [M – H]^–^ (calculated for C_19_H_17_O_3_, 293.1178); and ^1^H and ^13^C NMR spectroscopic data, Tables 1, 2.

***Otteacumiene B (2)****:* Yellow oil; [*α*] + 384.0 (*c* 0.098, MeOH); UV (MeOH) *λ*_max_ (log *ε*) 206.0 (3.73), 285 (2.57) nm; IR (KBr) *ν*_max_ 3400, 1630, 1594, 1515, 1503, 1432, 1212, 1135 cm^–1^; ECD (MeOH) *λ*_max_ (Δ*ε*) 246 (+ 31.34), 200 (– 17.13) nm; HREIMS *m/z* 294.1262 [M]^+^ (calculated for C_19_H_18_O_3_, 294.1256); and ^1^H and ^13^C NMR spectroscopic data were shown in Tables 1, 2.

***Otteacumiene C (3)****:* Colorless crystals; [*α*] + 1.8 (*c* 0.075, MeOH); [*α*] – 68.3 (*c* 0.048, MeOH) for (–)-**3**; [*α*] + 96.8 (*c* 0.050, MeOH) for (+)-**3**; UV (MeOH) *λ*_max_ (log*ε*) 278.5 (3.71), 196.0 (4.83), 258.0 (3.56) nm; IR (KBr) *ν*_max_ 3408, 1892, 1732, 1516, 1500, 1429, 1208, 1159, 1017 cm^–1^; ECD (MeOH) *λ*_max_ (Δ*ε*) 285 (+ 19.98), 270 (– 0.21), 259 (+ 2.96), 241 (– 78.41), 224 (+ 30.54), 213 (– 50.23), 204 (+ 1.22) nm for (+)-**3**; ECD (MeOH) *λ*_max_ (Δ*ε*) 285 (– 13.99), 270 (+ 0.22), 259 (– 2.71), 241 (+ 52.79), 224 (– 22.91), 213 (+ 31,32), 204 (– 2.74) nm for (–)-**3**; HRESIMS *m/z* 349.1407 [M + Na]^+^ (calculated for C_20_H_22_O_4_Na, 349.1410); and ^1^H and ^13^C NMR spectroscopic data were shown in Tables 1, 2.

***Otteacumiene D (4)****:* Yellow oil; [*α*] – 0.8 (*c* 0.120, MeOH); UV (MeOH) *λ*_max_ (log *ε*) 297.5 (4.48), 196.0 (5.09) nm; IR (KBr) *ν*_max_ 3390, 1703, 1612, 1587, 1508, 1430, 1069, 1053, 994 cm^–1^; HRESIMS *m/z* 293.1182 [M – H]^–^ (calculated for C_19_H_17_O_3_, 293.1178); and ^1^H and ^13^C NMR spectroscopic data, Tables 1, 2.

***Otteacumiene E (5)****:* Green oil; [*α*] – 0.2 (*c* 0.098, MeOH); UV (MeOH) *λ*_max_ (log *ε*) 200.0 (4.42), 235.4 (4.52), 279.0 (3.69) nm; IR (KBr) *ν*_max_ 3417, 3017, 2923, 1613, 1514, 1450, 1383, 1246, 1101, 826 cm^–1^; HRESIMS *m/z* 279.1390 [M – H]^–^ (calculated for C_19_H_19_O_2_, 279.1385); and ^1^H and ^13^C NMR spectroscopic data were shown in Tables 1, 2.

***Otteacumiene F (6)****:* Green oil; [*α*] – 0.6 (*c* 0.110, MeOH); UV (MeOH) *λ*_max_ (log *ε*) 200.2 (4.20), 229.6 (4.24), 277.6 (3.39) nm; IR (KBr) *ν*_max_ 3425, 2924, 1612, 1513, 1442, 1245, 1177, 1036, 826 cm^–1^; HRESIMS *m/z* 293.1547 [M – H]^–^ (calculated for C_20_H_21_O_2_ 293.1542); and ^1^H and ^13^C NMR spectroscopic data were shown in Tables 1, 2.

**Crystal data for 1**: C_19_H_18_O_3_, *M* = 294.33, *a* = 7.5455(4) Å, *b* = 9.2259(4) Å, *c* = 21.7568(10) Å, *α* = 90°, *β* = 90°, *γ* = 90°, *V* = 1514.58(12) Å^3^, *T* = 100.(2) K, space group *P*212121, *Z* = 4, *μ*(Cu Kα) = 0.695 mm^−1^, 27,150 reflections measured, 2871 independent reflections (*R*_*int*_ = 0.0429). The final *R*_*1*_ values were 0.0251 (*I* > 2*σ*(*I*)). The final *wR*(*F*^2^) values were 0.0643 (*I* > 2*σ*(*I*)). The final *R*_*1*_ values were 0.0255 (all data). The final *wR*(*F*^2^) values were 0.0645 (all data). The goodness of fit on *F*^2^ was 1.060. Flack parameter = 0.08(4). (CDDC: 2156830).

**Crystal data for 3**: C_20_H_22_O_4_∙CH_4_O, *M* = 358.42, *a* = 10.1053(9) Å, *b* = 18.5003(16) Å, *c* = 19.6923(17) Å, *α* = 90°, *β* = 90°, *γ* = 90°, *V* = 3681.5(6) Å^3^, *T* = 100.(2) K, space group *Pbca*, *Z* = 8, *μ*(Cu Kα) = 0.744 mm^−1^, 60,171 reflections measured, 3516 independent reflections (*R*_*int*_ = 0.0599). The final *R*_*1*_ values were 0.0366 (*I* > 2*σ*(*I*)). The final *wR*(*F*^2^) values were 0.0943 (*I* > 2*σ*(*I*)). The final *R*_*1*_ values were 0.0386 (all data). The final *wR*(*F*^2^) values were 0.0959 (all data). The goodness of fit on *F*^2^ was 1.070. (CDDC: 2155136).

### *α*-Glucosidase and PTB1B inhibitory activities assay

The *α*-glucosidase and PTB1B inhibitory activity were conducted according to the previous reports with slight modifications [49, 50]. Briefly, in the *α*-glucosidase inhibitory assay, after 50 min incubation at 37 °C, the absorbance value at 405 nm was detected and acarbose was used as positive control. While in the PTB1B inhibitory assay, after addition of phosphate-based detection reagent then incubation at 30 °C for 20 min, and absorbance was measured at 620 nm and suramin was used as positive control. The inhibition percentage was calculated as follows: inhibition rate (%) = (*E *– *S*)/E × 100% (*E* is the OD of the control and *S* is the OD of the sample) and IC_50_ (50% concentration of inhibition) was calculated by Reed and Muench method.

### Molecular docking studies

To explore the structure–activity relationship, the molecular docking studies was conducted according to the previous reports with slight modifications [51]. In brief, the AutoDock and PyMol software was used to blind docking between 3D structure of *α*-glucosidase which is downloaded from RCSB PDB website (PDB ID: 3A4A) and compound ligands.

## Supplementary Information

Below is the link to the electronic supplementary material.

Supplementary file1 (DOCX 8783 KB)
