# Supplementary material for: α-Glucosidase inhibitive diarylheptanoids from Ottelia acuminata var. acuminata, a traditional vegetable of Bai Nationality in Yunnan
Source: Nat Prod Bioprospect. 2022 Jun 10;12(1):22. doi: 10.1007/s13659-022-00341-4 (PMC9184688; doi:10.1007/s13659-022-00341-4)
Supplement: Supplementary file 1 — Supplementary file1 (DOCX 8783 KB) [file 13659_2022_341_MOESM1_ESM.docx]

***α*-Glucosidase Inhibitive Diarylheptanoids from *Ottelia acuminata*** **var. *acuminata*, a Traditional Vegetable of Bai Nationality in Yunnan**

Hong-Xing Liu^1,2,§^, Jun-Zeng Ma^1,§^, Yan-Song Ye^1^ , Jian-Jun Zhao^1^, Shi-Jie Wan^1^, Xin-Yue Hu^1,2^, Gang Xu^1,*^

^1^State Key Laboratory of Phytochemistry and Plant Resources in West China, Kunming Institute of Botany, Chinese Academy of Sciences, and Yunnan Key Laboratory of Natural Medicinal Chemistry, Kunming 650201, China

^2^University of Chinese Academy of Sciences, Beijing 100049, China

* Email: xugang008@mail.kib.ac.cn.

^§^Hong-Xing Liu and Jun-Zeng Ma contributed equally to this work.

Catalog

[**Figure S1.** Flow chart of extraction and separation 5](#_Toc99784725)

[**Figure S2.** Individuals of racemic mixtures (±)-**3** isolate by chiral resolution^a^ 8](#_Toc99784726)

[**Figure S3.** ^1^H NMR spectrum of compound **1** (MeOD, 600 MHz) 9](#_Toc99784727)

[**Figure S4.** ^13^C NMR and DEPT spectra of compound **1** (MeOD, 150 MHz) 9](#_Toc99784728)

[**Figure S5.** HSQC spectrum of compound **1** 10](#_Toc99784729)

[**Figure S6.** ^1^H-^1^H COSY spectrum of compound **1** 10](#_Toc99784730)

[**Figure S7.** HMBC spectrum of compound **1** 11](#_Toc99784731)

[**Figure S8.** HRESIMS spectrum of compound **1** 12](#_Toc99784732)

[**Figure S9.** IR spectrum of compound **1** 13](#_Toc99784733)

[**Figure S10.** ECD (top) and UV (bottom) spectra of compound **1** 15](#_Toc99784734)

[**Figure S11.** ^1^H NMR spectrum of compound **2** (MeOD, 600 MHz) 16](#_Toc99784735)

[**Figure S12.**^13^C NMR and DEPT spectra of compound **2** (MeOD, 150 MHz) 16](#_Toc99784736)

[**Figure S13.** HSQC spectrum of compound **2** 17](#_Toc99784737)

[**Figure S14.** ^1^H-^1^H COSY spectrum of compound **2** 17](#_Toc99784738)

[**Figure S15.** HMBC spectrum of compound **2** 18](#_Toc99784739)

[**Figure S16.** HREIMS spectrum of compound **2** 19](#_Toc99784740)

[**Figure S17.** IR spectrum of compound **2** 20](#_Toc99784741)

[**Figure S18.** ECD (top) and UV (bottom) spectra of compound **2** 22](#_Toc99784742)

[**Figure S19.** ^1^H NMR spectrum of compound (±)-**3** (MeOD, 600 MHz) 23](#_Toc99784743)

[**Figure S20.** ^13^C NMR and DEPT spectrum of compound (±)-**3** (MeOD, 150 MHz) 23](#_Toc99784744)

[**Figure S21.** HSQC spectrum of compound (±)-**3** 24](#_Toc99784745)

[**Figure S22.** ^1^H-^1^H COSY spectrum of compound (±)-**3** 24](#_Toc99784746)

[**Figure S23.** HMBC spectrum of compound (±)-**3** 25](#_Toc99784747)

[**Figure S24.** ROESY spectrum of compound (±)-**3** 25](#_Toc99784748)

[**Figure S25.** HRESIMS spectrum of compound (±)-**3** 26](#_Toc99784749)

[**Figure S26.** IR spectrum of compound (±)-**3** 27](#_Toc99784750)

[**Figure S27.** ECD (top) and UV (bottom) spectra of compound (+)-**3** 29](#_Toc99784751)

[**Figure S28.** ECD (top) and UV (bottom) spectra of compound (−)-**3** 31](#_Toc99784752)

[**Figure S29.** ^1^H NMR spectrum of compound **4** (MeOD, 600 MHz) 32](#_Toc99784753)

[**Figure S30.** ^13^C NMR and DEPT spectra of compound **4** (MeOD, 150 MHz) 32](#_Toc99784754)

[**Figure S31.** HSQC spectrum of compound **4** 33](#_Toc99784755)

[**Figure S32.** ^1^H-^1^H COSY spectrum of compound **4** 33](#_Toc99784756)

[**Figure S33.** HMBC spectrum of compound **4** 34](#_Toc99784757)

[**Figure S34.** HRESIMS spectrum of compound **4** 35](#_Toc99784758)

[**Figure S35.** IR spectrum of compound **4** 36](#_Toc99784759)

[**Figure S36.** ECD (top) and UV (bottom) spectra of compound **4** 38](#_Toc99784760)

[**Figure S37.** ^1^H NMR spectrum of compound **5** (CD_3_COCD_3_, 600 MHz) 39](#_Toc99784761)

[**Figure S38.** ^13^C NMR and DEPT spectrum of compound **5** (CD_3_COCD_3_, 150 MHz) 39](#_Toc99784762)

[**Figure S39.** HSQC spectrum of compound **5** 40](#_Toc99784763)

[**Figure S40**. ^1^H-^1^H COSY spectrum of compound **5** 40](#_Toc99784764)

[**Figure S41.** HMBC spectrum of compound **5** 41](#_Toc99784765)

[**Figure S42.** HRESIMS spectrum of compound **5** 42](#_Toc99784766)

[**Figure S43.** IR spectrum of compound **5** 43](#_Toc99784767)

[**Figure S44.** UV spectrum of compound **5** 44](#_Toc99784768)

[**Figure S45.** ^1^H NMR spectrum of compound **6** (CD_3_COCD_3_, 600 MHz) 45](#_Toc99784769)

[**Figure S46.** ^13^C NMR and DEPT spectra of compound **6** (CD_3_COCD_3_, 150 MHz) 45](#_Toc99784770)

[**Figure S47.** HSQC spectrum of compound **6** 46](#_Toc99784771)

[**Figure S48.** ^1^H-^1^H COSY spectrum of compound **6** 46](#_Toc99784772)

[**Figure S49.** HMBC spectrum of compound **6** 47](#_Toc99784773)

[**Figure S50.** HRESIMS spectrum of compound **6** 48](#_Toc99784774)

[**Figure S51.** IR spectrum of compound **6** 49](#_Toc99784775)

[**Figure S53.** Optimized geometries of 4 dominate conformers of **2** at the M06-2X-D3/def2-SVP level of theory in the gas phase. 52](#_Toc99784776)

[**Table S1.** Conformational analysis of the M06-2X-D3/def2-SVP optimized conformers of **2** in the gas phase (T=298.15 K) 52](#_Toc99784777)

[**Table S2.** Atomic coordinates (Å) of **2-1** obtained at the M06-2X-D3/def2-SVP level of theory in the gas phase. 53](#_Toc99784778)

[**Table S3.** Atomic coordinates (Å) of **2-2** obtained at the M06-2X-D3/def2-SVP level of theory in the gas phase. 54](#_Toc99784779)

[**Table S4.** Atomic coordinates (Å) of **2-3** obtained at the M06-2X-D3/def2-SVP level of theory in the gas phase. 55](#_Toc99784780)

[**Table S5.** Atomic coordinates (Å) of **2-4** obtained at the M06-2X-D3/def2-SVP level of theory in the gas phase. 56](#_Toc99784781)

[**Figure S54.** Optimized geometries of 4 dominate conformers of **+3** at the M06-2X-D3/def2-SVP level of theory in the gas phase. 57](#_Toc99784782)

[**Table S6.** Conformational analysis of the M06-2X-D3/def2-SVP optimized conformers of **+3** in the gas phase (T=298.15 K) 57](#_Toc99784783)

[**Table S7.** Atomic coordinates (Å) of **+3-1** obtained at the M06-2X-D3/def2-SVP level of theory in the gas phase. 58](#_Toc99784784)

[**Table S8.** Atomic coordinates (Å) of **+3-2** obtained at the M06-2X-D3/def2-SVP level of theory in the gas phase. 59](#_Toc99784785)

[**Table S9.** Atomic coordinates (Å) of **+3-3** obtained at the M06-2X-D3/def2-SVP level of theory in the gas phase. 60](#_Toc99784786)

[**Table S10.** Atomic coordinates (Å) of **+3-4** obtained at the M06-2X-D3/def2-SVP level of theory in the gas phase. 61](#_Toc99784787)

[**Table S11.** Inhibitory effects of **1**–**8** against PTP1B^a^ 62](#_Toc99784788)

# **Figure S1.** Flow chart of extraction and separation

- Detailed Extraction and isolation:

Fr. A (4.7 g) was divided into five sub-fractions (Fr. A-1–A-5) by silica gel column chromatography with a gradient system of petroleum ether (PE)/acetone (PA) (300:1–0:1, v/v). Fr. A-2 (725.3 mg) was further separated by preparative HPLC (MeOH-H_2_O from 6:4 to 10:0, v/v) to yield Fr. A-2-1–A-2-4. Fr. A-2-2 and A-2-3 were purified by semi-preparative HPLC (ZORBAX SB-C8, 250 × 9.4 mm, 5.0 *μ*m) to obtain **5** (3.9 mg) and **8** (1.7 mg), respectively.

Fraction B (6.0 g) was further chromatographed over a silica gel column, eluted with PE/PA (300:1-0:1, v/v), to obtain six sub-fractions (Fr. B-1–B-6). Compound **17** (2.5 mg) were purified from B-2 (824 mg) by preparative HPLC. Fr. B-3 (940 mg) was separated on a silica gel column chromatography eluting with PE/ethyl acetate (EA) (400:1–0:1, v/v) to give four Fr. B-3-1–B-3-4. And B-3-3 was separated by semi-preparative HPLC obtained **1** (4.9 mg). Fr. B-4 (694 mg) was subjected on silica gel column chromatography (eluted by PE/PA, 400:1–0:1, v/v) and then purified using semi-preparative HPLC to afford **36** (2.1 mg) and **37**(1.6 mg).

Fr. C (5.2 g) was divided into four sub-fractions (Fr. C-1–C-4) separated by RP-C18 column chromatography (MeOH-H_2_O from 6:4 to 10:0, v/v). The Fr. C-2 (1254.2 mg) was chromatographed over a silica gel column eluted with PE/PA (300:1-0:1, v/v) to obtain eight fractions (Fr. C-2-1–C-2-5). And Fr. C-2-2 was separated by semi-preparative HPLC obtained **16** (6.1mg).

Fr. D (3.8 g) was separated by RP-C18 column chromatography (MeOH-H_2_O from 6:4 to 10:0, v/v) to yield five sub-fractions (Fr. D-1–D-5). Fr. D-1 (513.3 mg) was separated by silica gel column chromatography with a gradient system of PE/EA (400:1–100:1, v/v) to yield five sub-fractions (Fr. D-1–D-5). Fr. D-1-2 was separated by semi-preparative HPLC obtained **25** (2.6 mg) and **26** (6.9 mg). Similarly, **14** (4.9 mg), **29** (1.7 mg), **31** (1.9 mg), **13** (6.0 mg), **27** (4.5 mg), **28** (2.8 mg), **12** (4.3 mg), **15** (1.9 mg) were afforded from Fr. D-2, D-3, D-4, and D-5, respectively, by silica gel column chromatography and semi-preparative HPLC.

Fr. E (4.4 g) was divided into four sub-fractions (Fr. E-1–E-4) by RP-C18 column chromatography (MeOH-H_2_O from 6:4 to 10:0, v/v). Fr. E-1 (841.7 mg) was separated by silica gel column chromatography with a gradient system of PE/PA (300:1–0:1, v/v) to yield five sub-fractions (Fr. E-1-1–E-1-5). Fr. E-1-3 was purified by semi-preparative HPLC obtained **6** (2.3 mg) and **33** (6.7 mg). Compounds **4** (7.1 mg), **30** (4.6 mg), **32** (5.5 mg), **34** (1.8 mg), **35** (5.7 mg), **7** (3.2 mg), **41** (8.5 mg) were afforded from Fr. E-2, E-3, and E-4, respectively, by the same method.

Fr. F (4.0 g) was divided into six sub-fractions (Fr. F-1–F-6) through silica gel column chromatography with a gradient system of PE/PA (200:1–0:1, v/v). Fr. F-1 (409.9 mg) was separated by silica gel column chromatography with a gradient system of chloroform (TCM)/methyl alcohol (MT) (10:0–0:10, v/v) to yield three sub-fractions (Fr. F-1-1–F-1-3). Fr. F-1-2 was separated by semi-preparative HPLC to obtain **22** (2.4 mg), **39** (6.5 mg), and **40** (2.2 mg). Fr. F-2, F-3, F-4, F-5, and F-6 were subjected on silica gel column chromatography (eluted by TCM/MT, 10:0–0:10, v/v) and then purified using semi-preparative HPLC to afford **9** (4.4 mg), **23** (6.5 mg), **24** (2.0 mg), **3** (3.0 mg), **10** (8.8 mg), **11** (2.1 mg), **18** (2.9 mg), **38 (**1.9 mg), **2** (1.3 mg), **20** (1.7 mg), **21** (2.1 mg), and **19** (1.6 mg)

^a^ DAICEL CORPORATION semi-preparative column (CNCH_3_/H_2_O, 30:70, v/v)

# **Figure S2.** Individuals of racemic mixtures (±)-**3** isolate by chiral resolution^a^


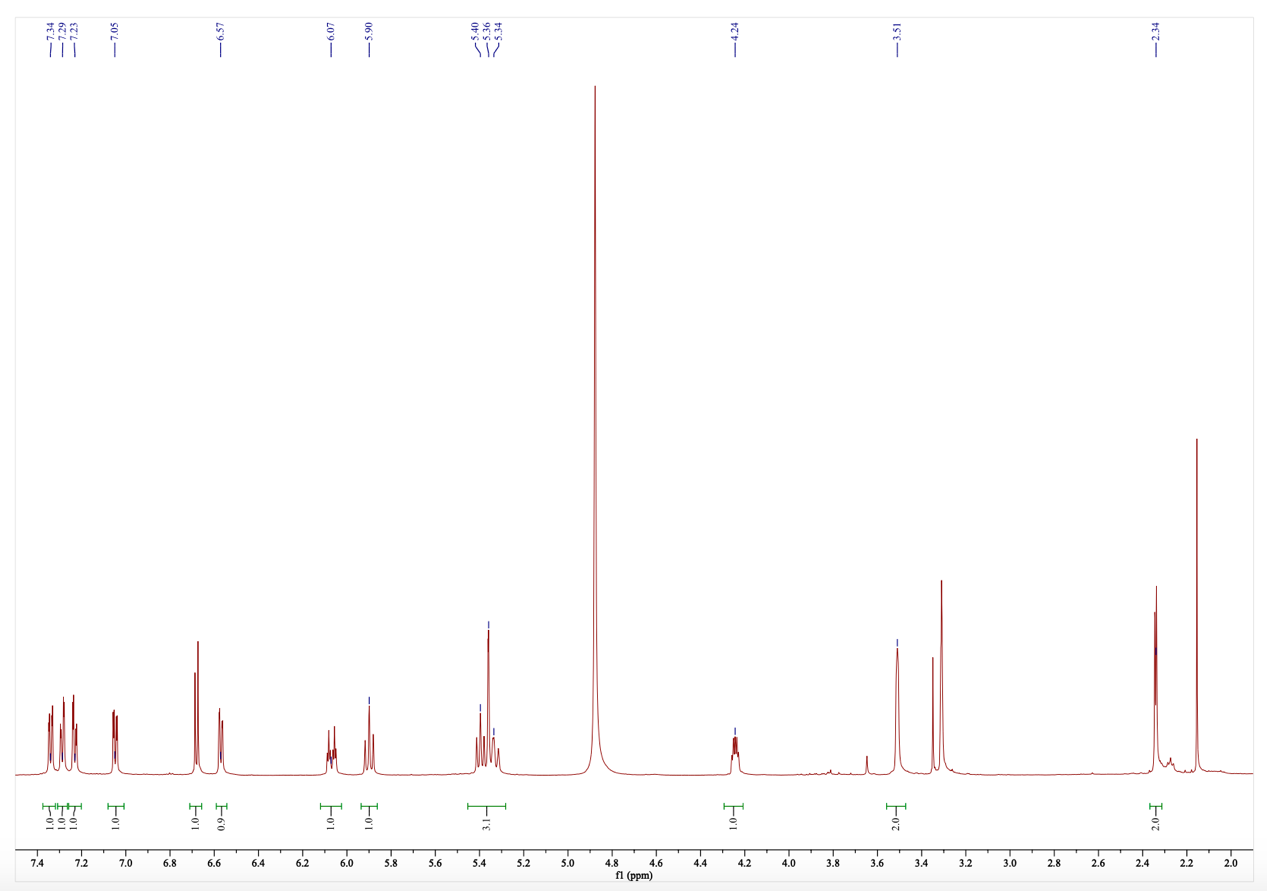


# **Figure S3.** ^1^H NMR spectrum of compound **1** (MeOD, 600 MHz)


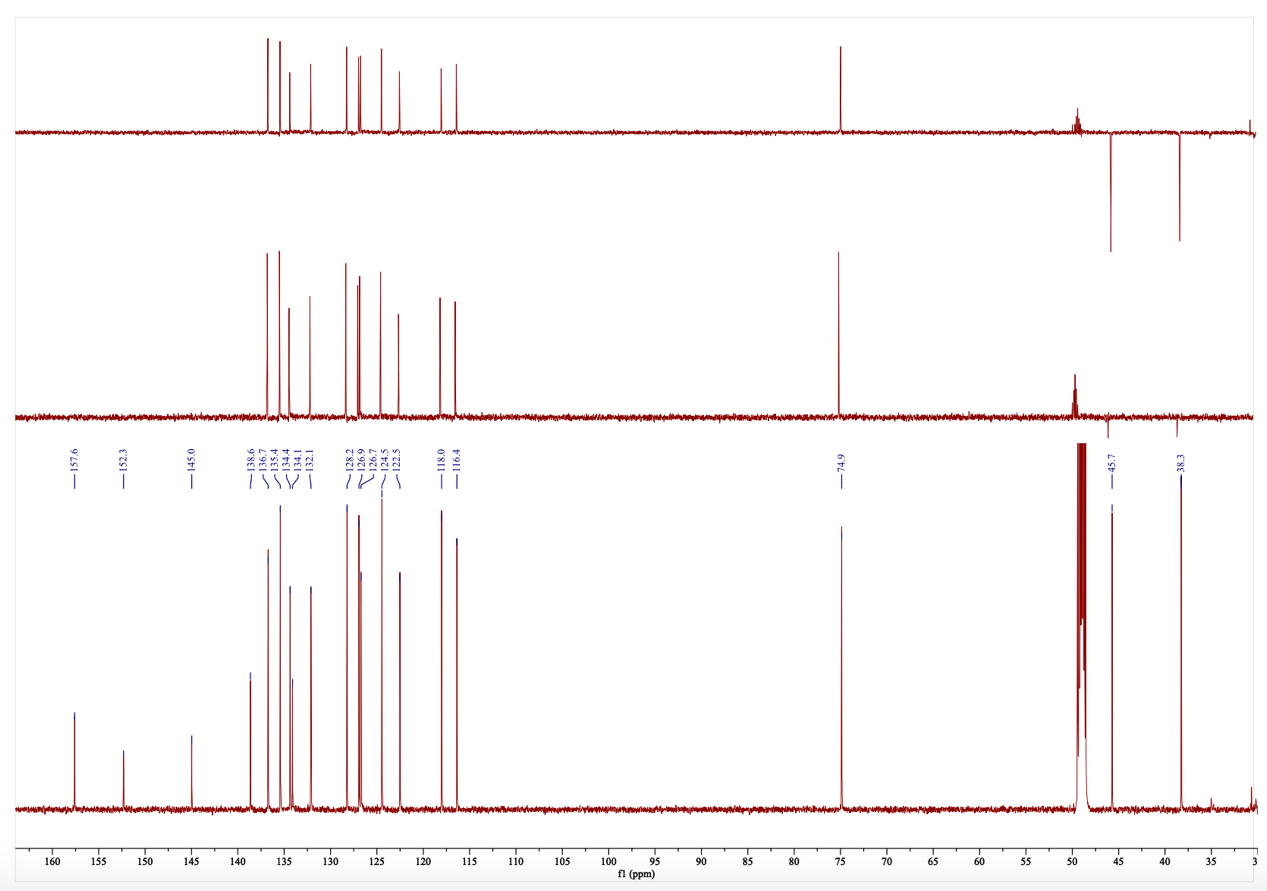


# **Figure S4.** ^13^C NMR and DEPT spectra of compound **1** (MeOD, 150 MHz)


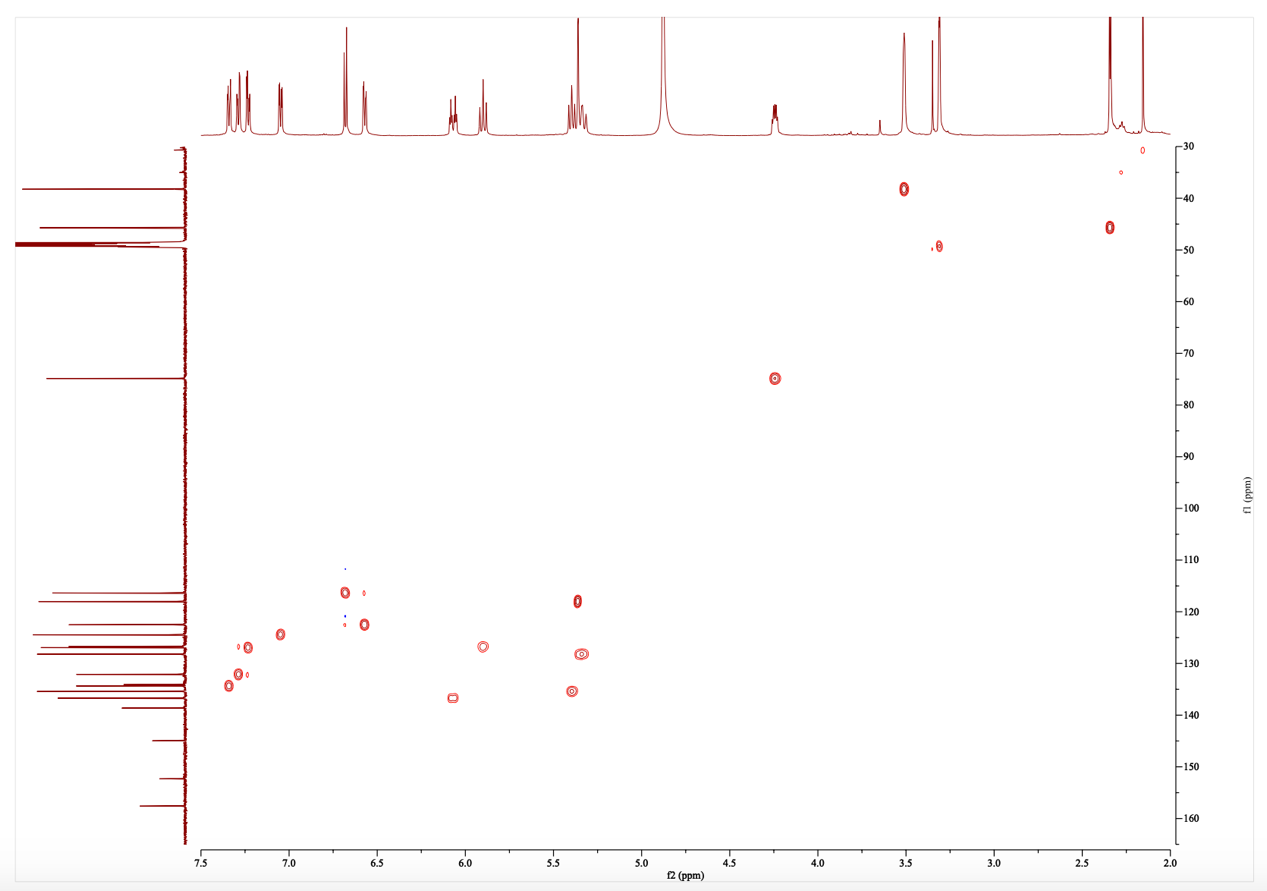


# **Figure S5.** HSQC spectrum of compound **1**


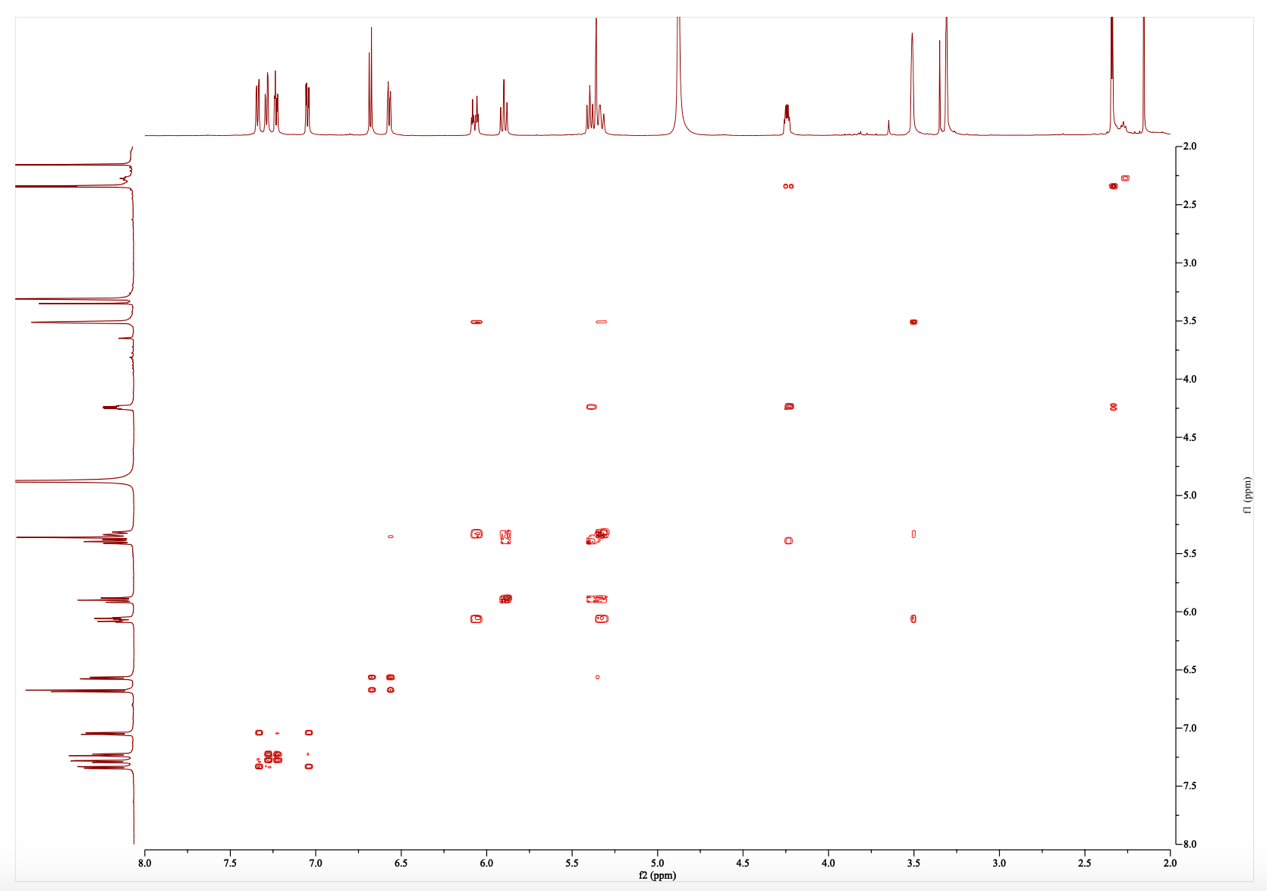


# **Figure S6.** ^1^H-^1^H COSY spectrum of compound **1**


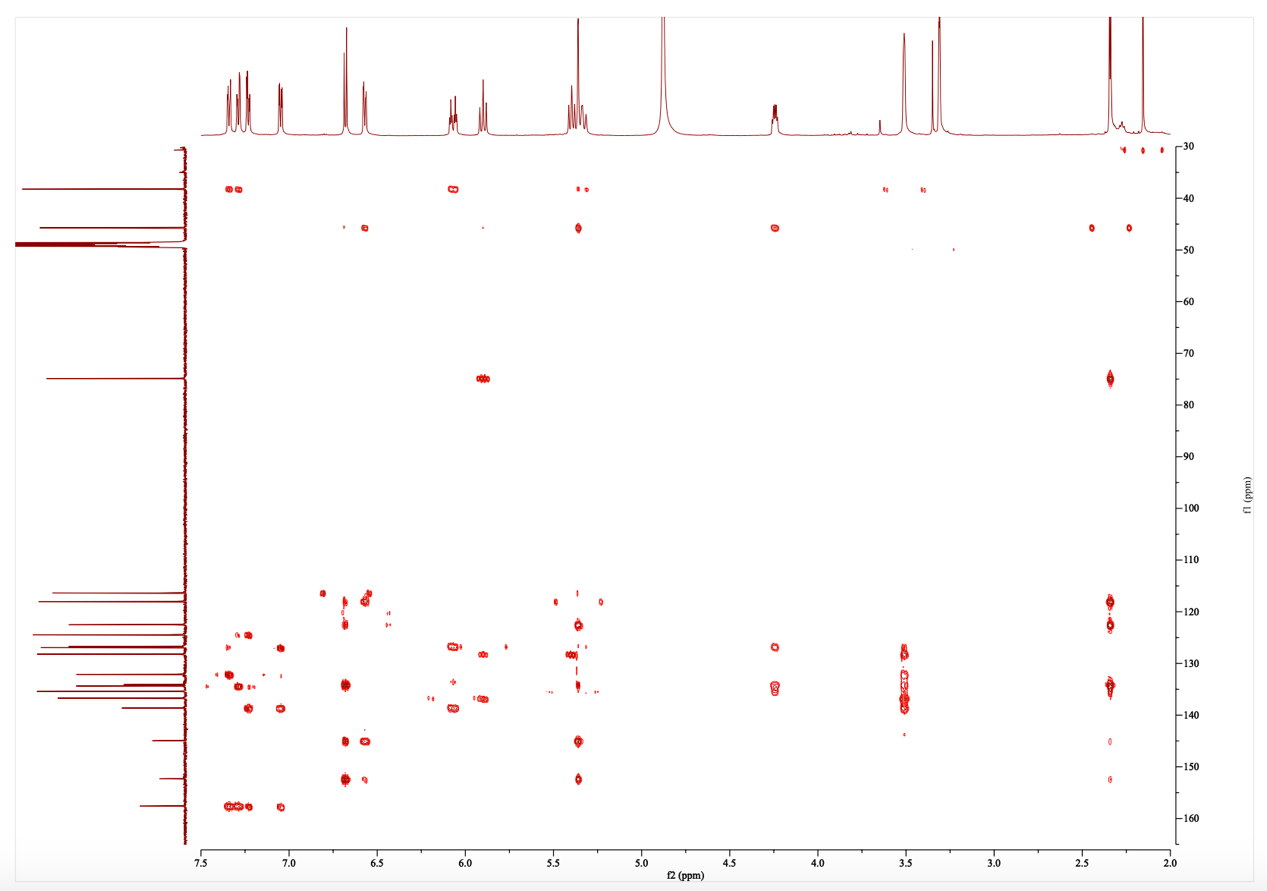


# **Figure S7.** HMBC spectrum of compound **1**


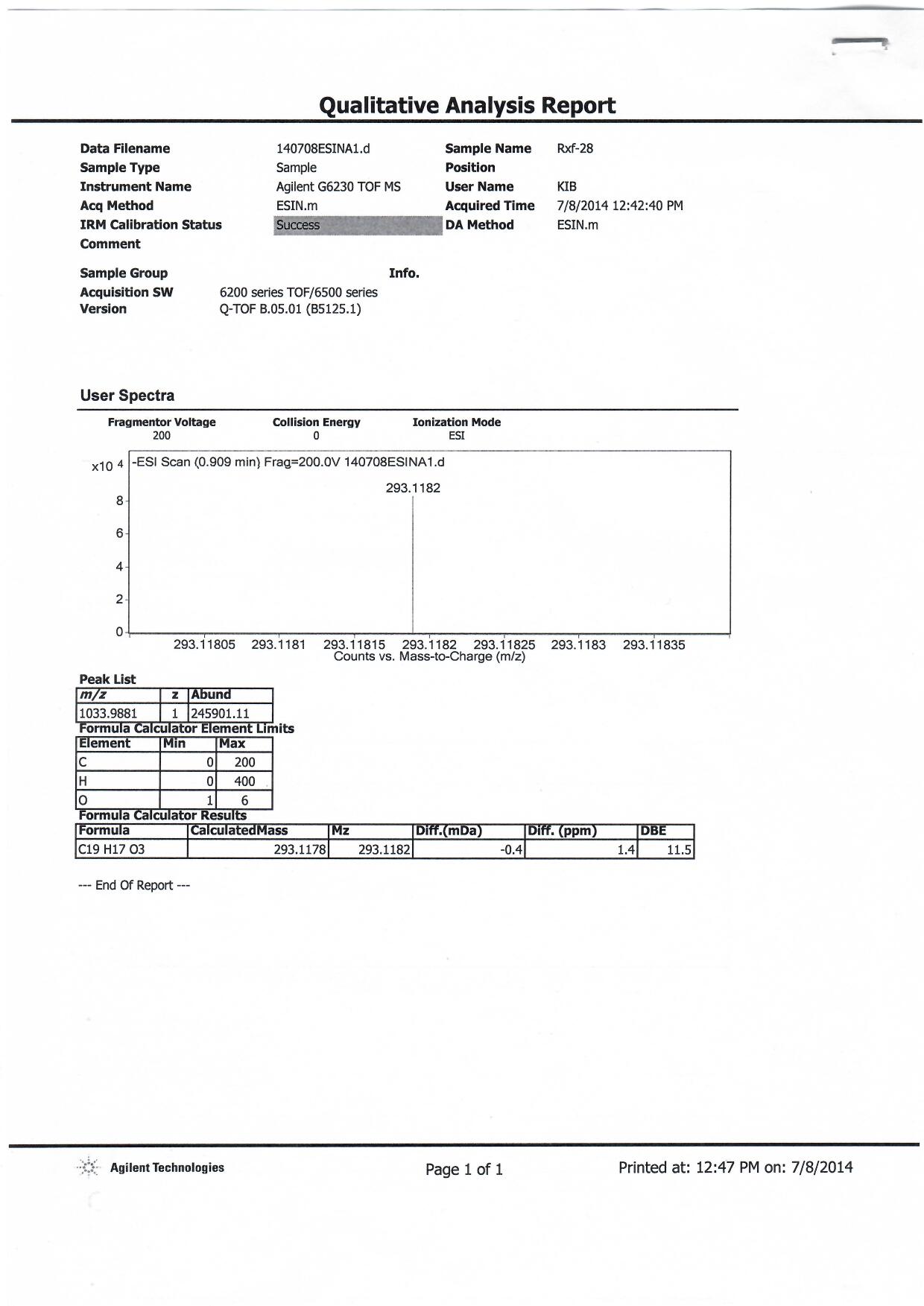


# **Figure S8.** HRESIMS spectrum of compound **1**

# **Figure S9.** IR spectrum of compound **1**


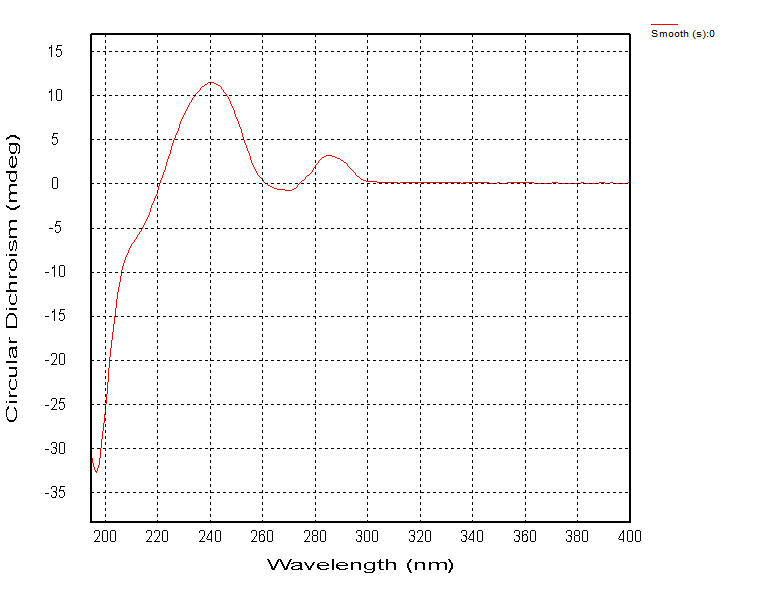


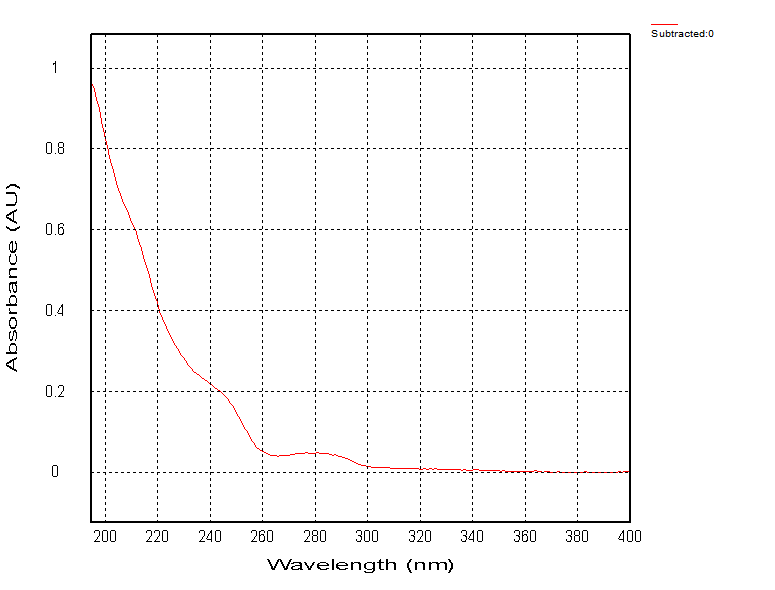


File: RXV-28.dsx

ProBinaryX

Attributes :

- Time Stamp :Mon Jan 10 16:05:19 2022

- File ID : {F4EDA52A-0F52-4182-8641-BFE74418871F}

- Is CFR Compliant : false

- Original unaltered data

Remarks:

- User: CD

- Date: 2022/01/10

- Instrument: 0547

- DetectorType: LAAPD

- DichOS Calibration Correction Curve: 0547/2

- HV (CDDC channel): 0 v

- Time per point: 1 s

- Description: Sample 1

- Concentration: 0.0750mg/mLMeOH

- Pathlength: 1 mm

- Temperature: 20℃

Settings:

- HV

- Time-per-point: 1s (25us x 40000)

- SE

- Wavelength: 195nm - 400nm

- Step Size: 1nm

- Bandwidth: 1nm

# **Figure S10.** ECD (top) and UV (bottom) spectra of compound **1**


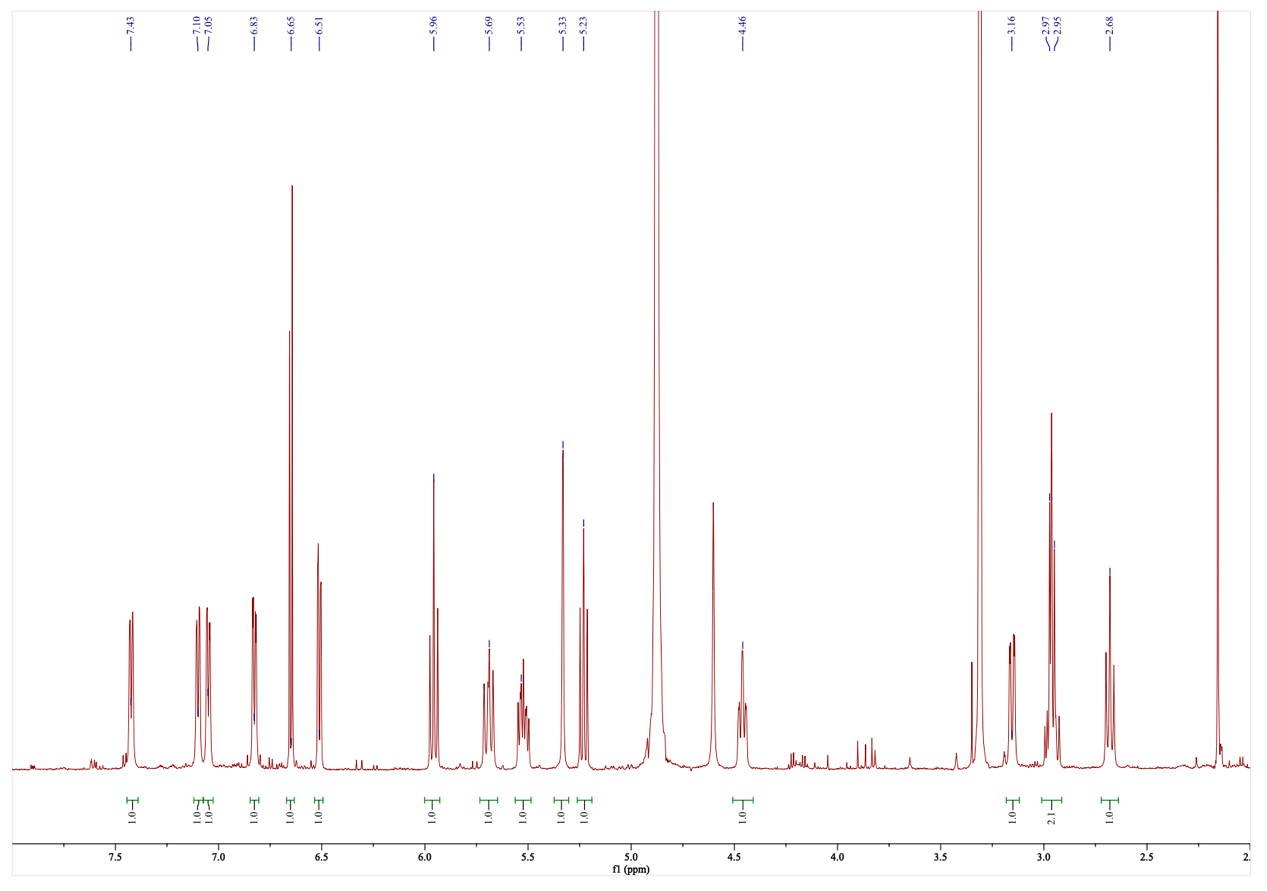


# **Figure S11.** ^1^H NMR spectrum of compound **2** (MeOD, 600 MHz)


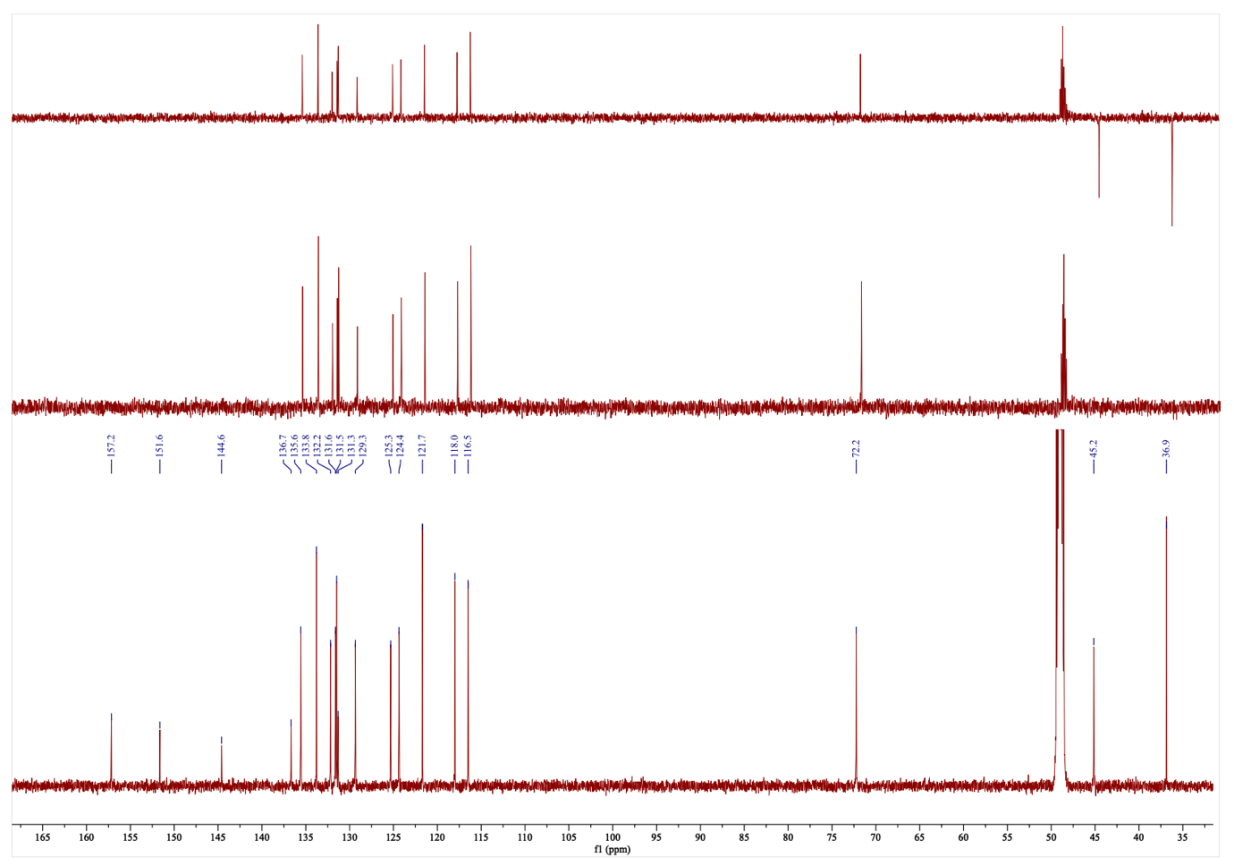


# **Figure S12.**^13^C NMR and DEPT spectra of compound **2** (MeOD, 150 MHz)


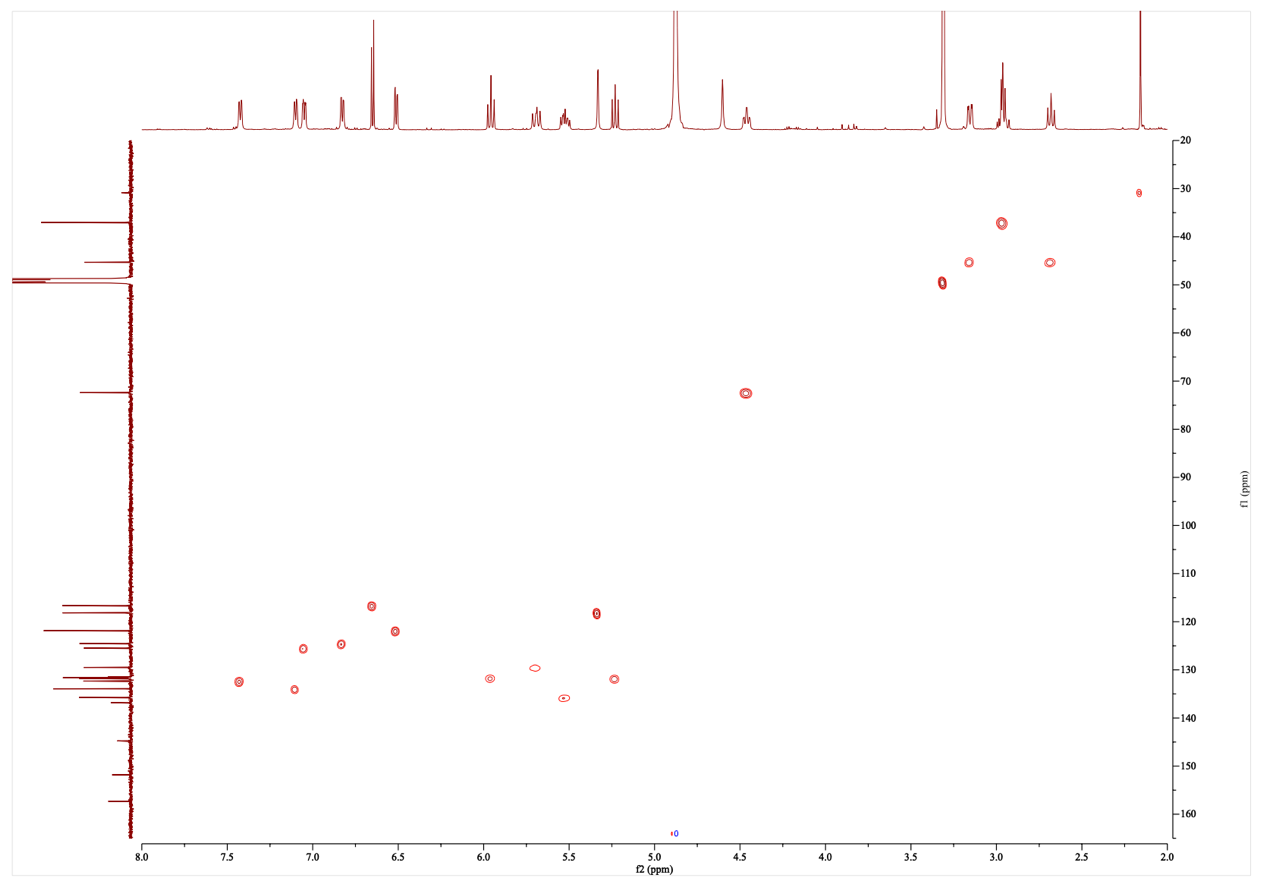


# **Figure S13.** HSQC spectrum of compound **2**


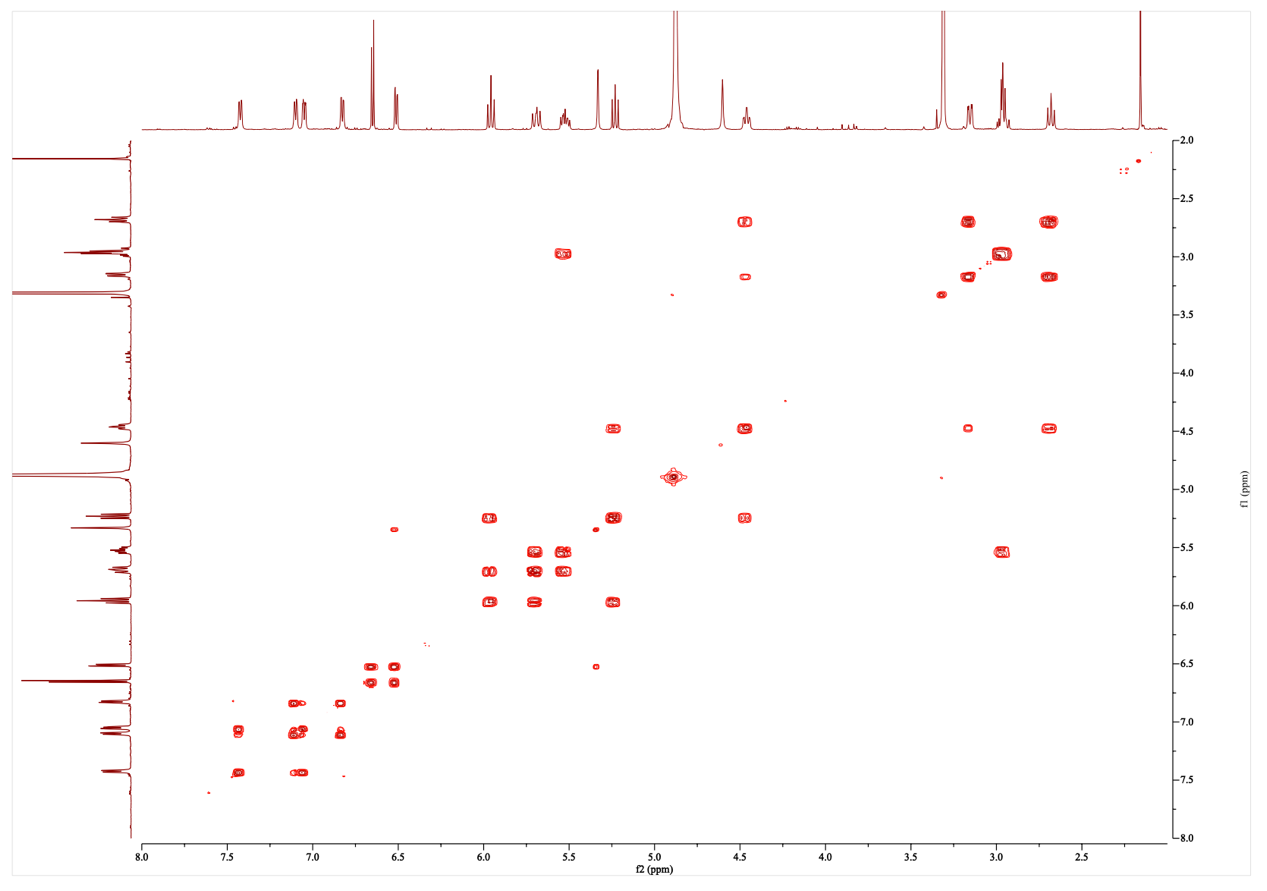


# **Figure S14.** ^1^H-^1^H COSY spectrum of compound **2**


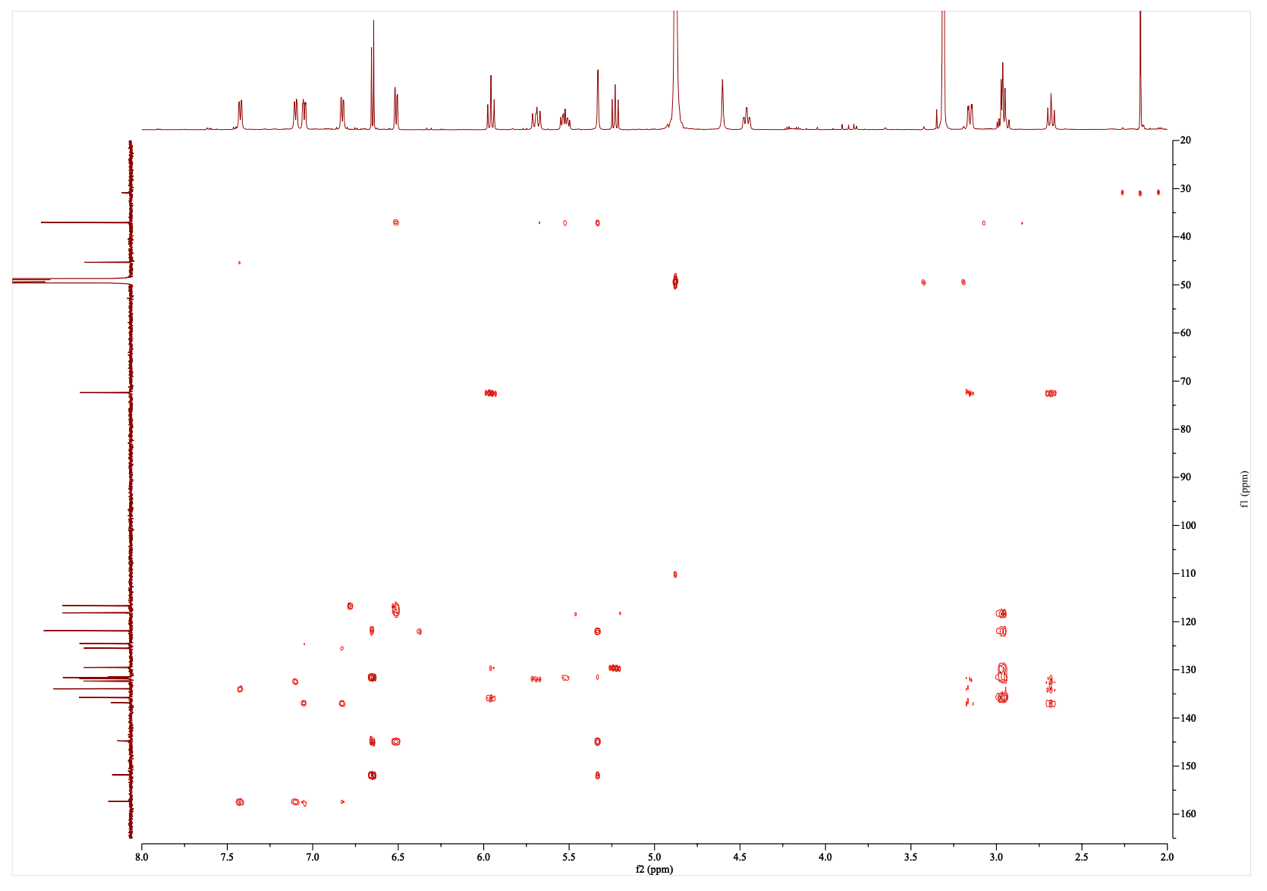


# **Figure S15.** HMBC spectrum of compound **2**


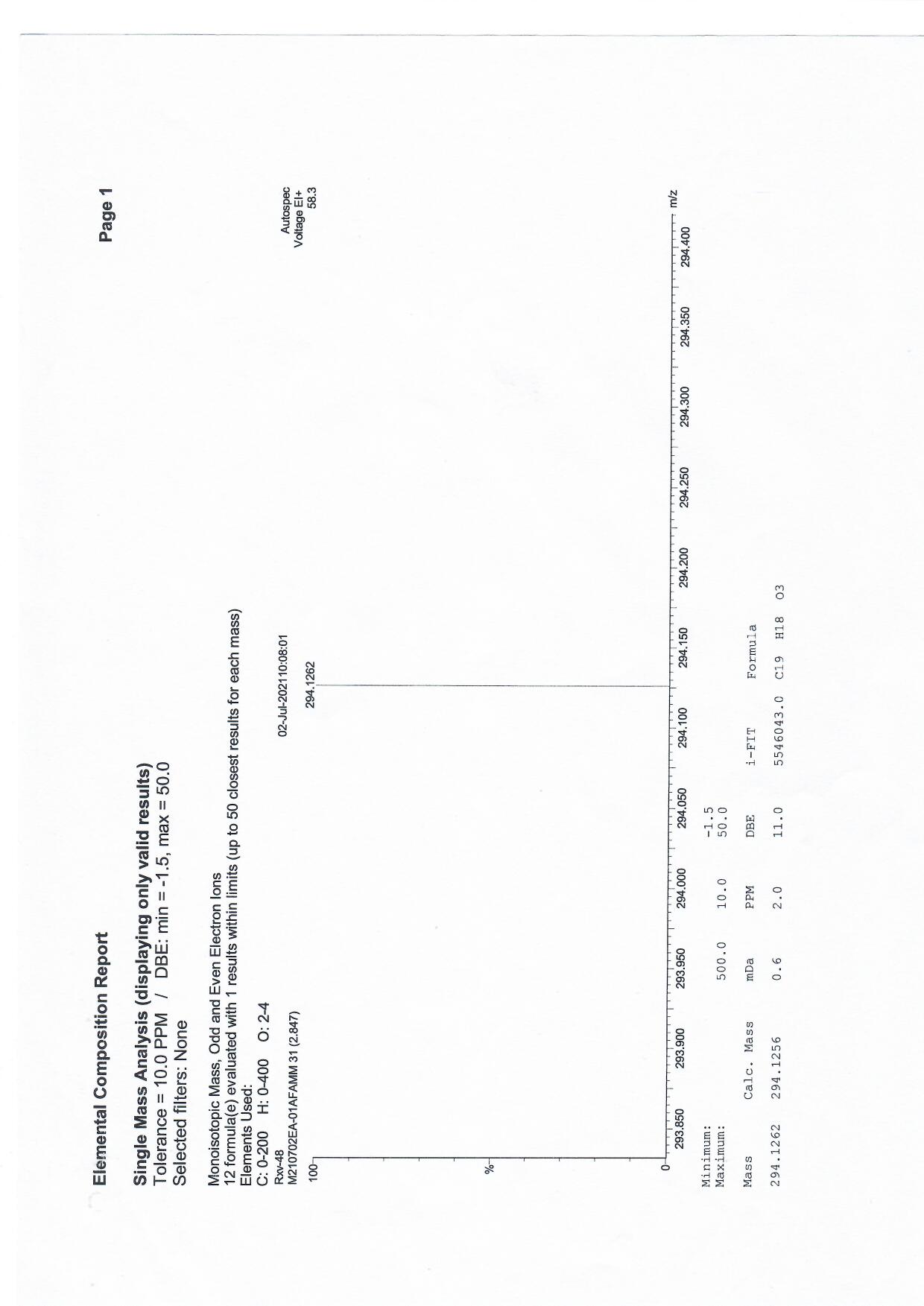


# **Figure S16.** HREIMS spectrum of compound **2**

# **Figure S17.** IR spectrum of compound **2**


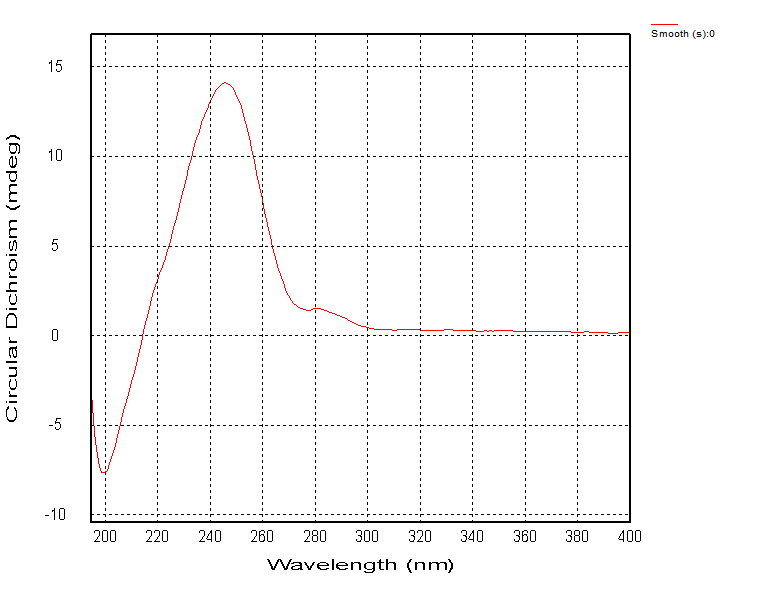


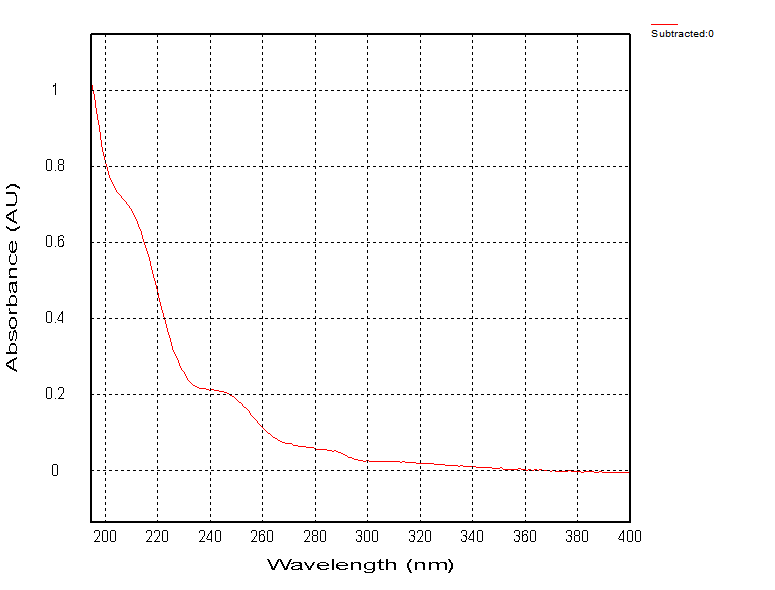


File: RXV-48-1mm(195-400)21090121.dsx

ProBinaryX

Attributes :

- Time Stamp :Wed Sep 01 20:59:36 2021

- File ID : {FA64FFC3-3545-46ac-9AC2-768203D41067}

- Is CFR Compliant : false

- Original data has not been modified.

Remarks:

- User: CD

- Date: 2021/09/01

- Instrument: 0547

- DetectorType: LAAPD

- DichOS Calibration Correction Curve: 0547/2

- HV (CDDC channel): 0 v

- Time per point: 1 s

- Description: Sample 1

- Concentration: 0.0400mg/mL MeOH

- Pathlength: 1 mm

- Temperature: 20℃

Settings:

- Time-per-point: 1s (25us x 40000)

- SE

- Wavelength: 195nm - 400nm

- Step Size: 1nm

- Bandwidth: 1nm

# **Figure S18.** ECD (top) and UV (bottom) spectra of compound **2**


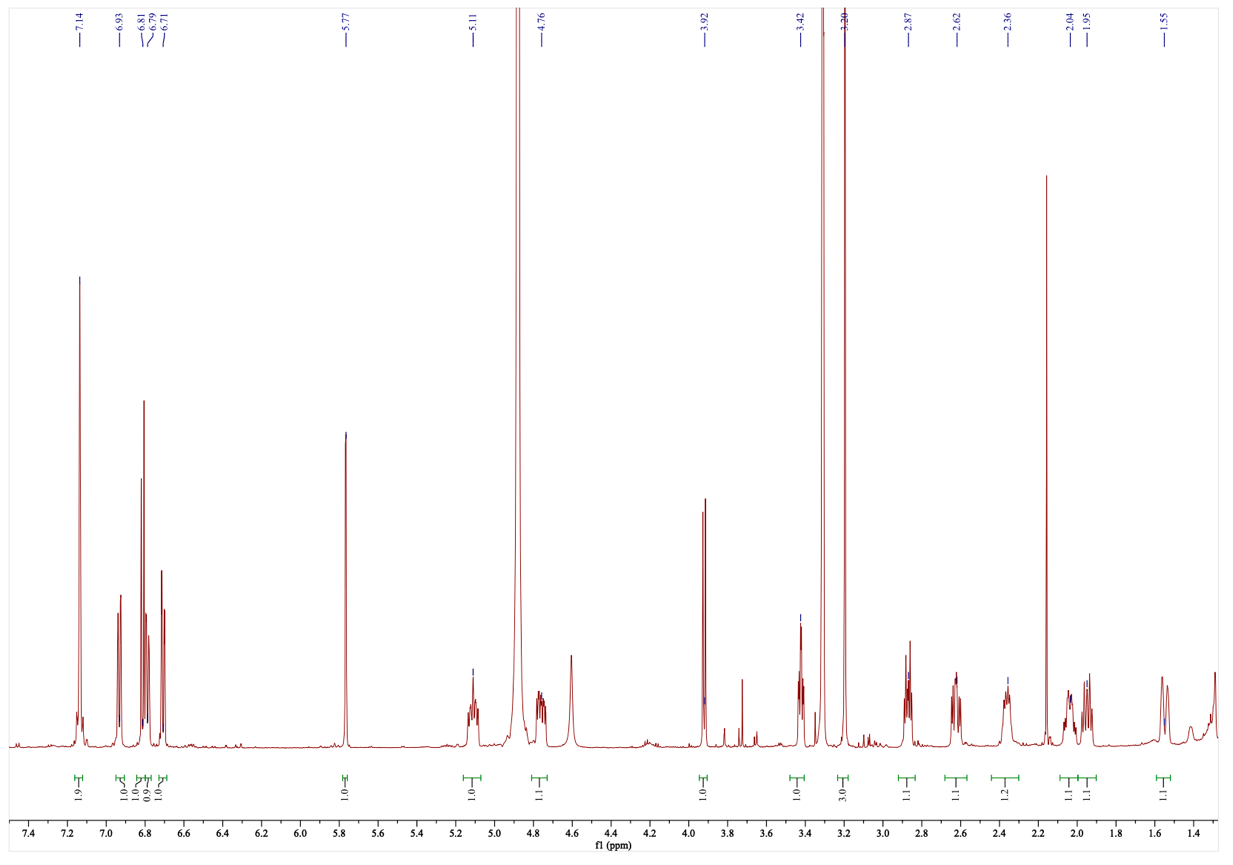


# **Figure S19.** ^1^H NMR spectrum of compound (±)-**3** (MeOD, 600 MHz)


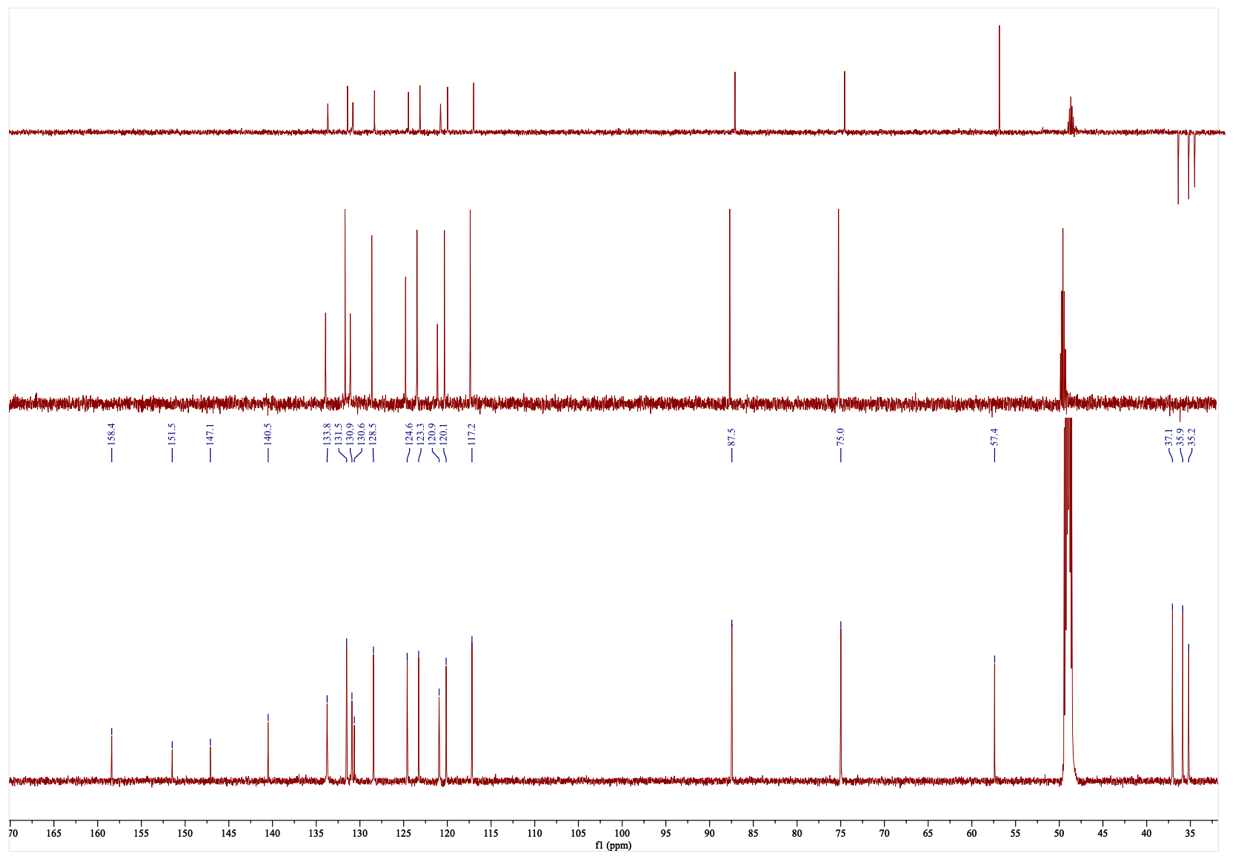


# **Figure S20.** ^13^C NMR and DEPT spectrum of compound (±)-**3** (MeOD, 150 MHz)


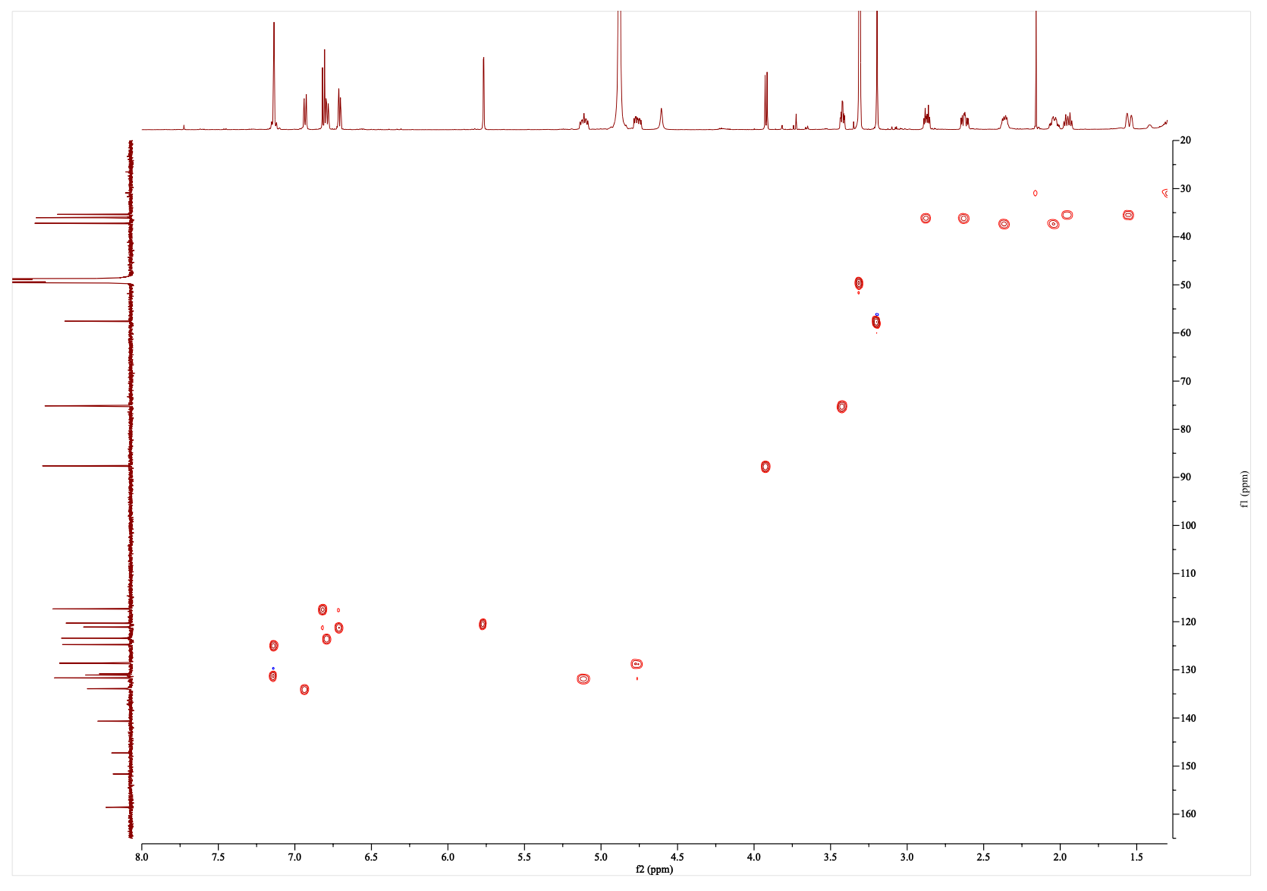


# **Figure S21.** HSQC spectrum of compound (±)-**3**


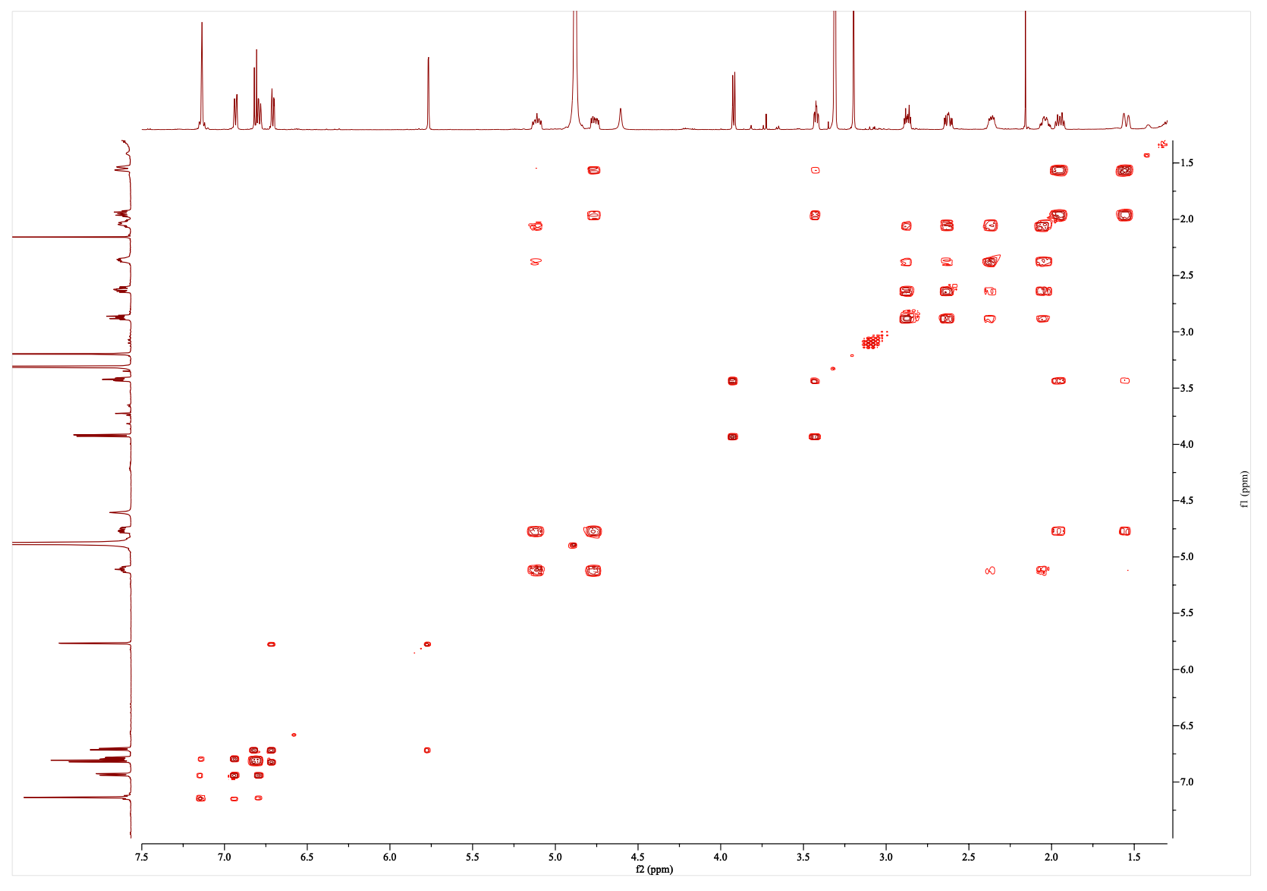


# **Figure S22.** ^1^H-^1^H COSY spectrum of compound (±)-**3**


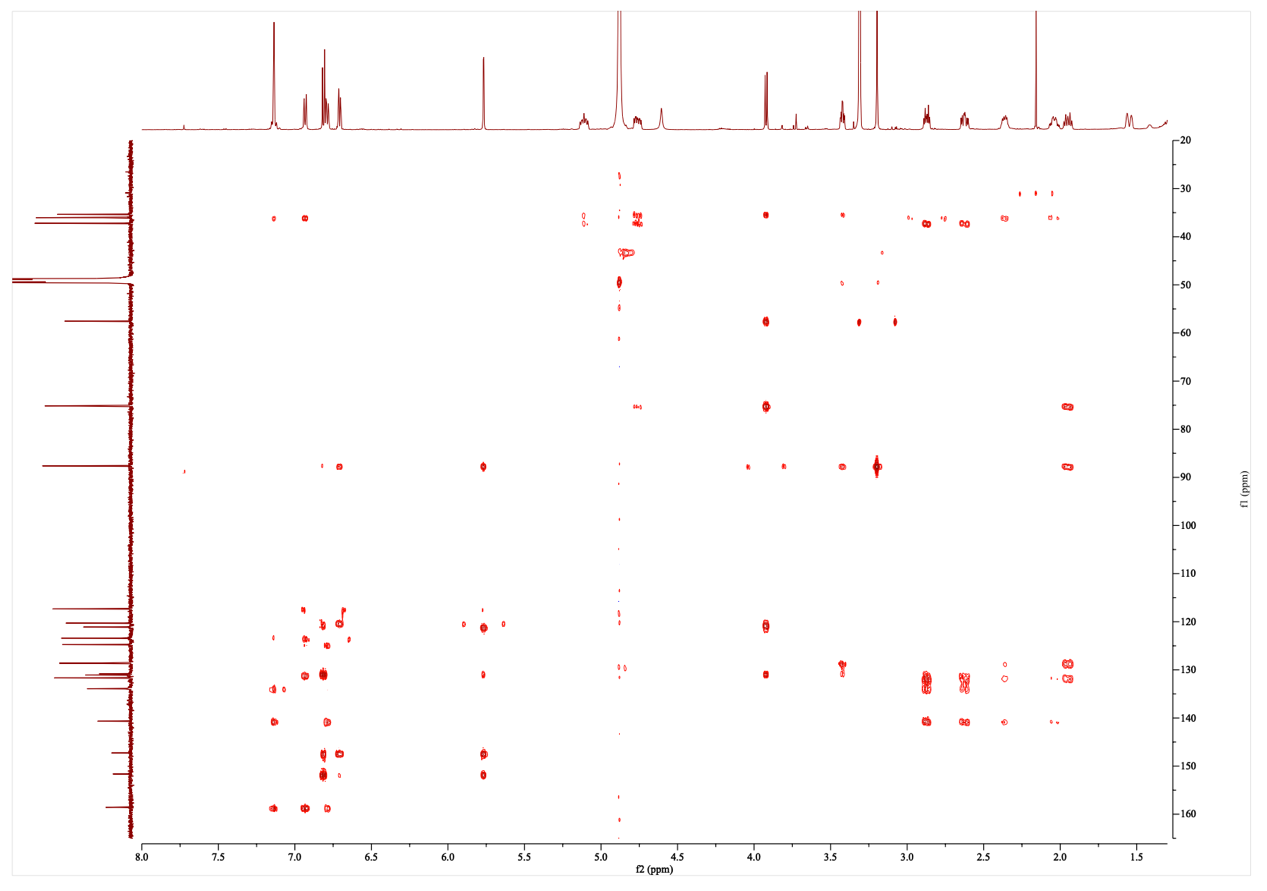


# **Figure S23.** HMBC spectrum of compound (±)-**3**


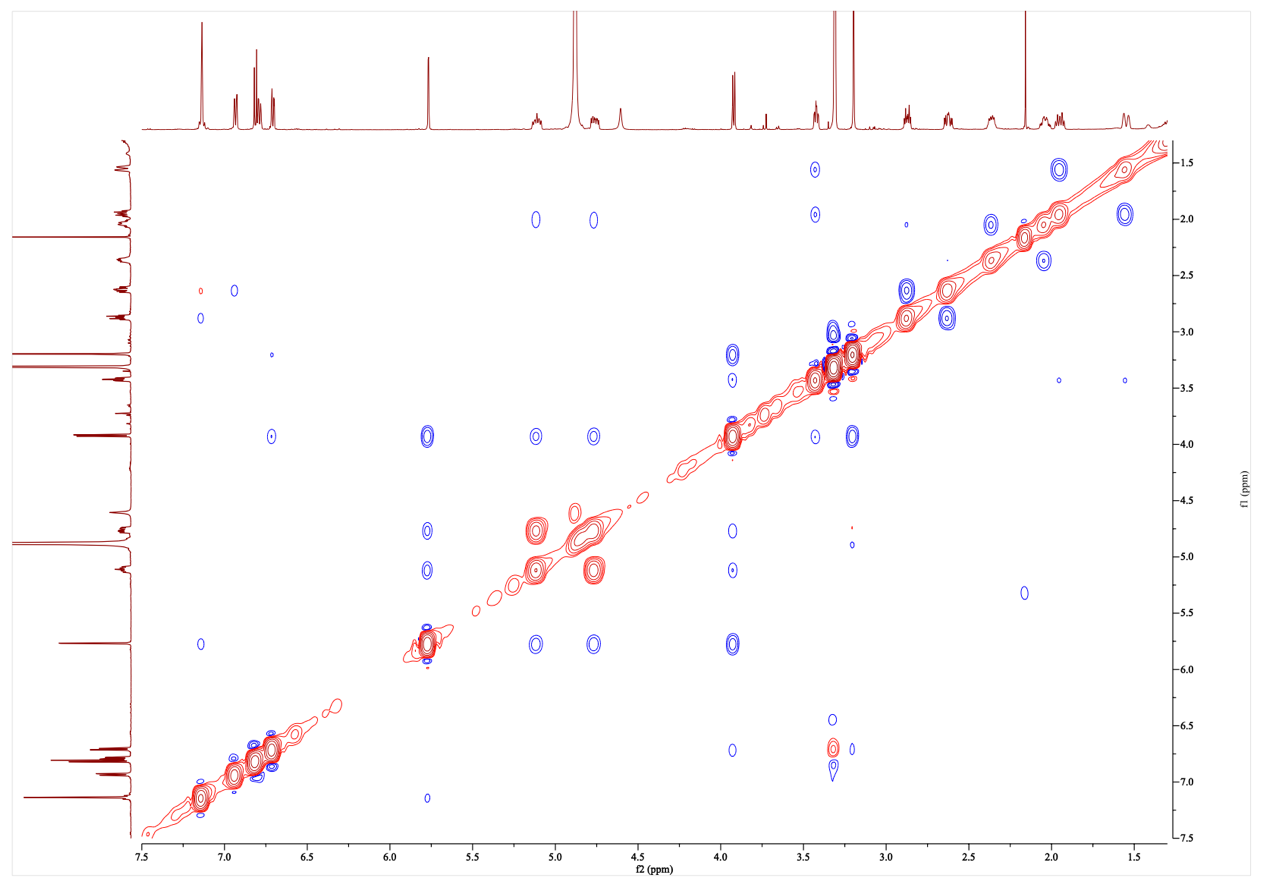


# **Figure S24.** ROESY spectrum of compound (±)-**3**


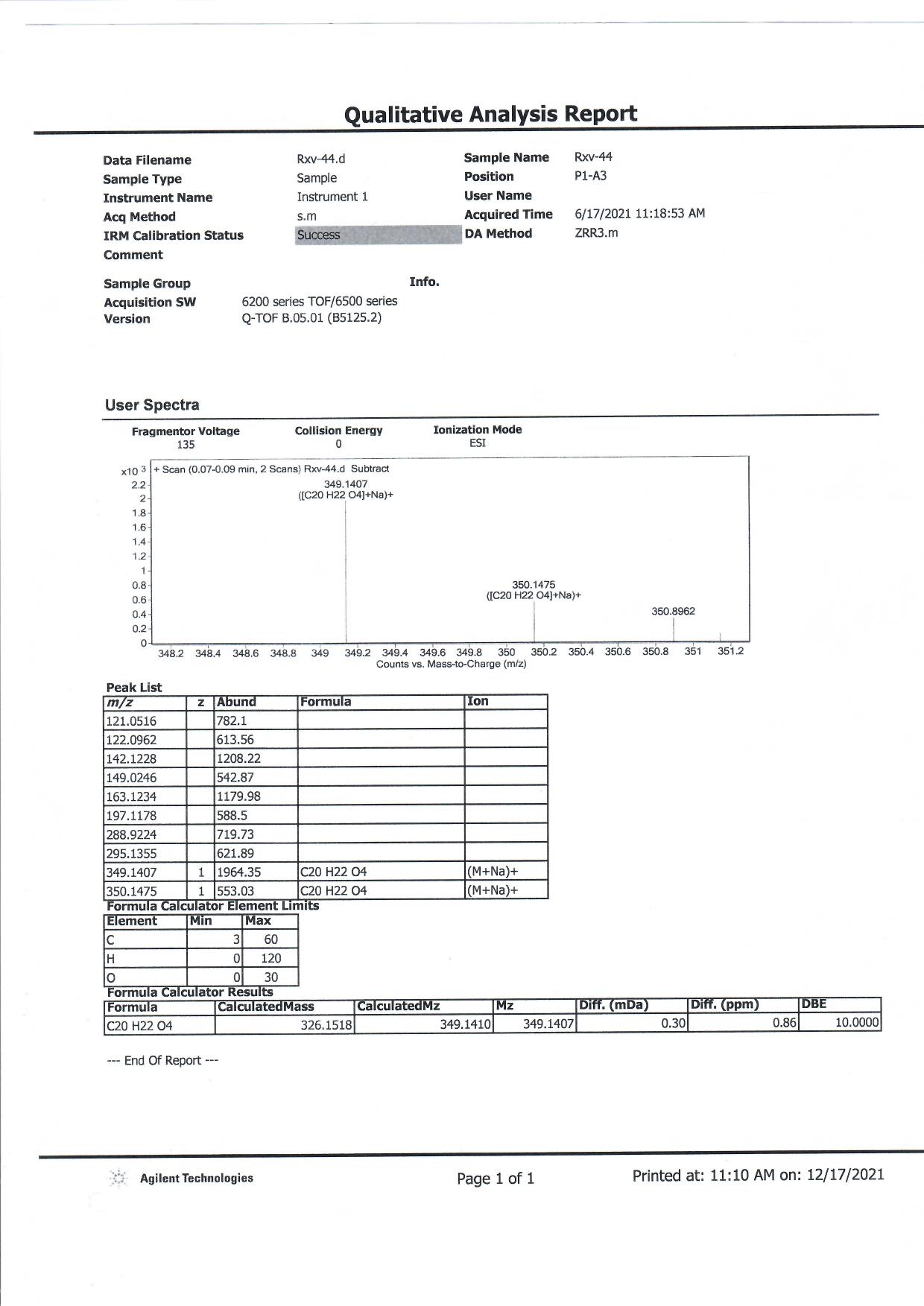


# **Figure S25.** HRESIMS spectrum of compound (±)-**3**


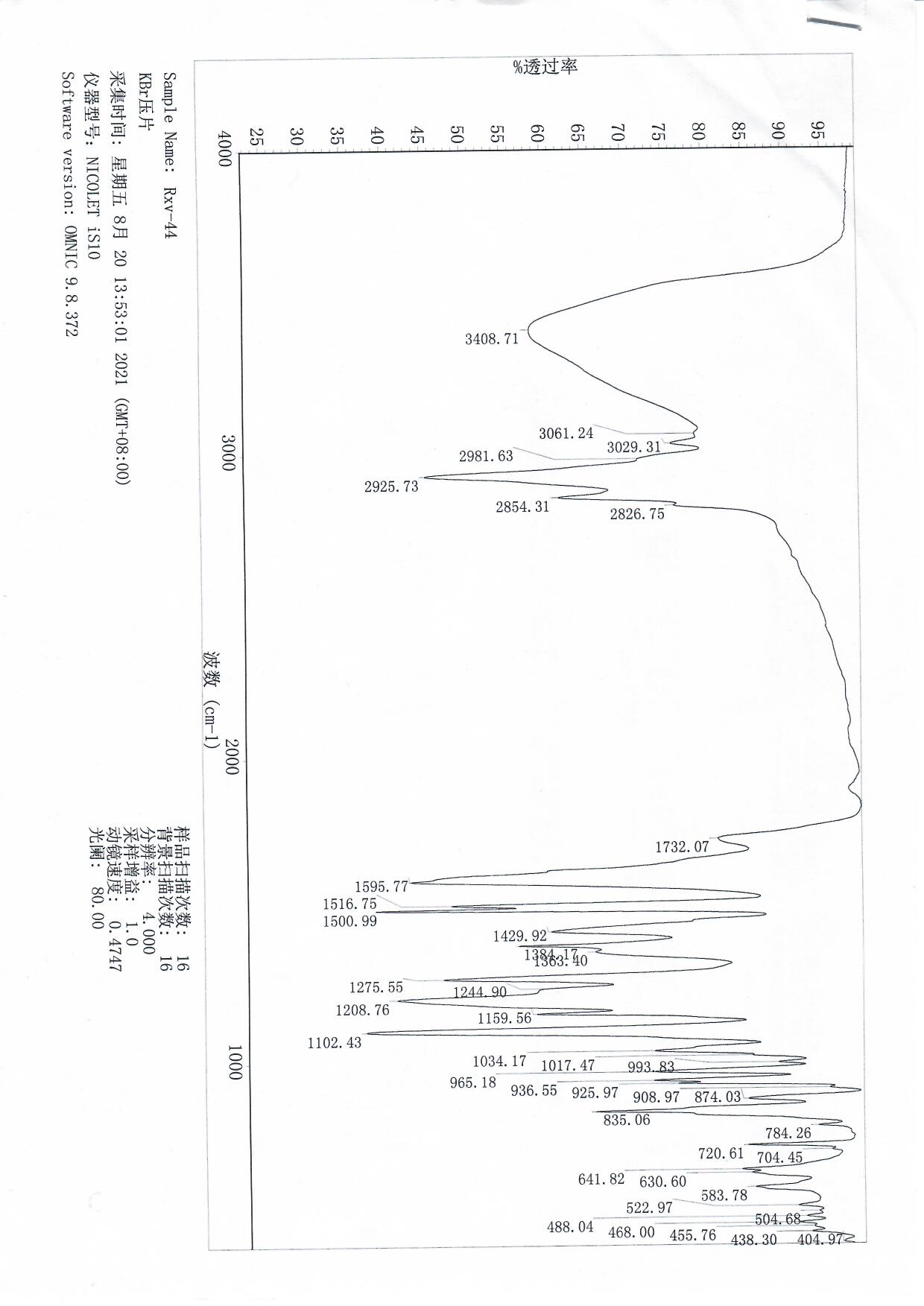


# **Figure S26.** IR spectrum of compound (±)-**3**


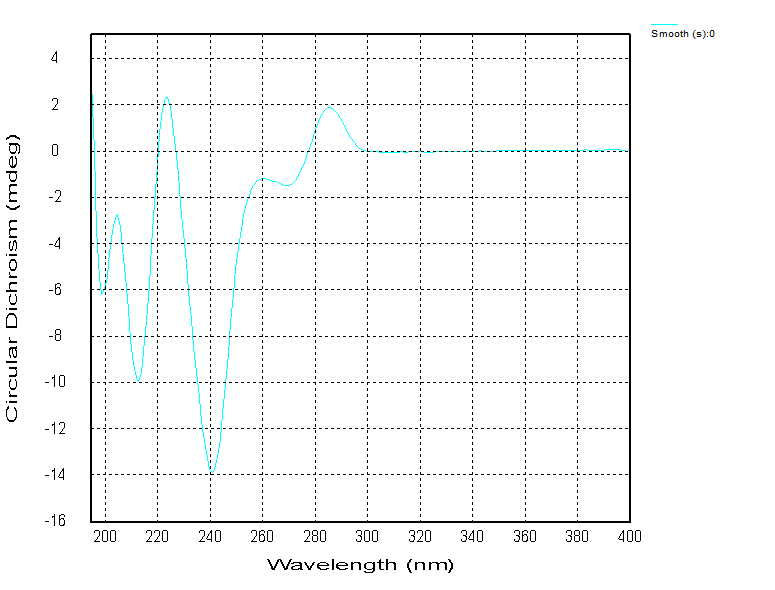


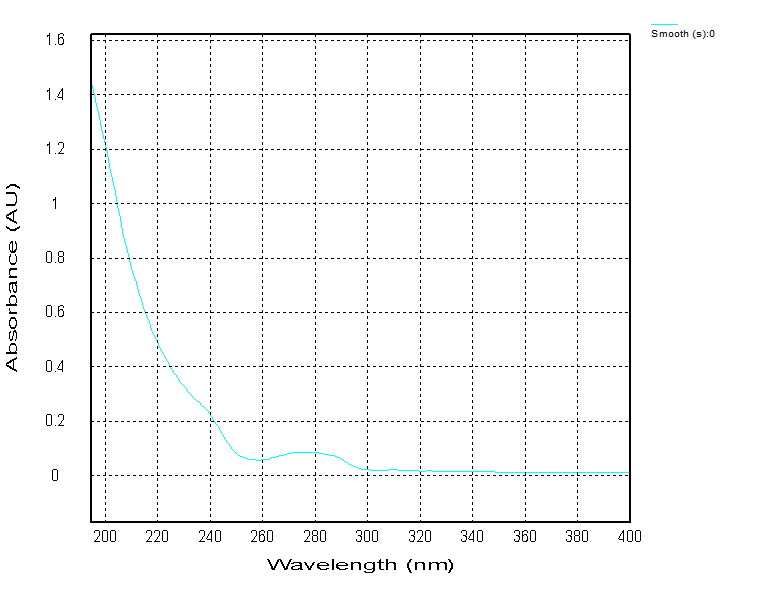


File: RXV-44A-1mm(195-400)21093012.dsx

ProBinaryX

Attributes :

- Time Stamp :Thu Sep 30 17:06:33 2021

- File ID : {DD1A2C7C-D8E1-4ca8-823A-DC98EAB55D00}

- Is CFR Compliant : false

- Original unaltered data

Remarks:

- User: CD

- Date: 2021/09/30

- Instrument: 0547

- DetectorType: LAAPD

- DichOS Calibration Correction Curve: 0547/2

- HV (CDDC channel): 0 v

- Time per point: 1 s

- Description: Sample 1

- Concentration: 0.0918mg/mLMeOH

- Pathlength: 1 mm

- Temperature: 20℃

Settings:

- HV

- Time-per-point: 1s (25us x 40000)

- SE

- Wavelength: 195nm - 400nm

- Step Size: 1nm

- Bandwidth: 1nm

# **Figure S27.** ECD (top) and UV (bottom) spectra of compound (+)-**3**


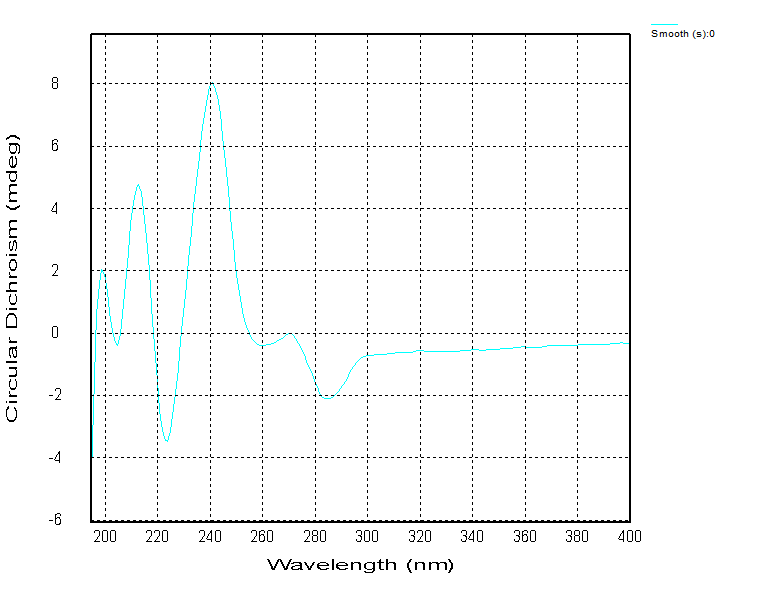


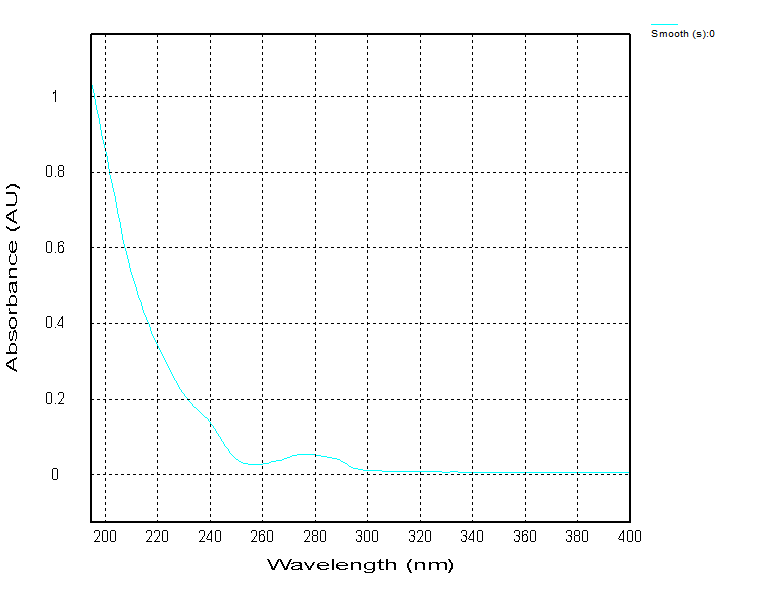


File: RXV-44B-1mm(195-400)21093013.dsx

ProBinaryX

Attributes :

- Time Stamp :Thu Sep 30 17:26:05 2021

- File ID : {F1EDFB00-634F-40fb-874A-58FD90398B6A}

- Is CFR Compliant : false

- Original unaltered data

Remarks:

- User: CD

- Date: 2021/09/30

- Instrument: 0547

- DetectorType: LAAPD

- DichOS Calibration Correction Curve: 0547/2

- HV (CDDC channel): 0 v

- Time per point: 1 s

- Description: Sample 1

- Concentration: 0.0905mg/mLMeOH

- Pathlength: 1 mm

- Temperature: 20℃

Settings:

- HV

Time-per-point: 1s (25us x 40000)

- SE

- Wavelength: 195nm - 400nm

- Step Size: 1nm

- Bandwidth: 1nm

# **Figure S28.** ECD (top) and UV (bottom) spectra of compound (−)-**3**


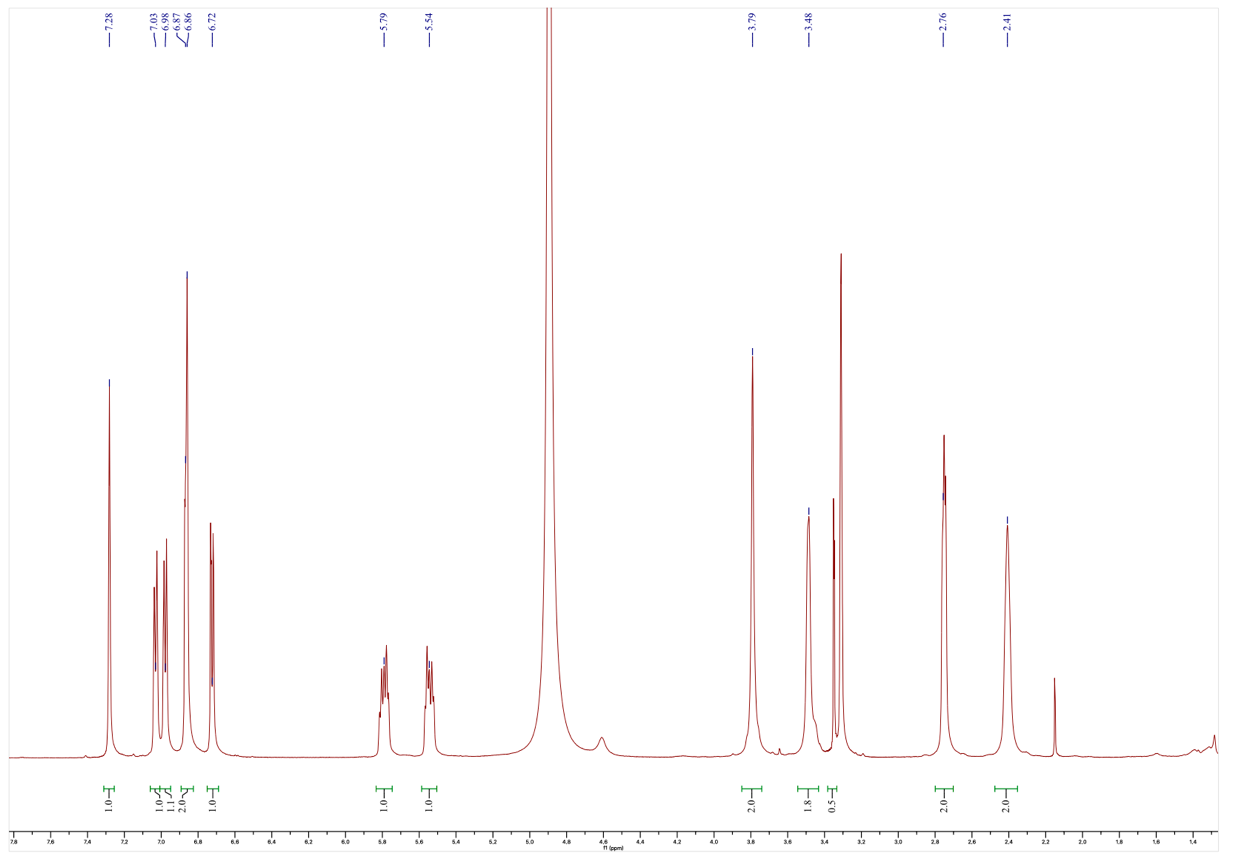


# **Figure S29.** ^1^H NMR spectrum of compound **4** (MeOD, 600 MHz)


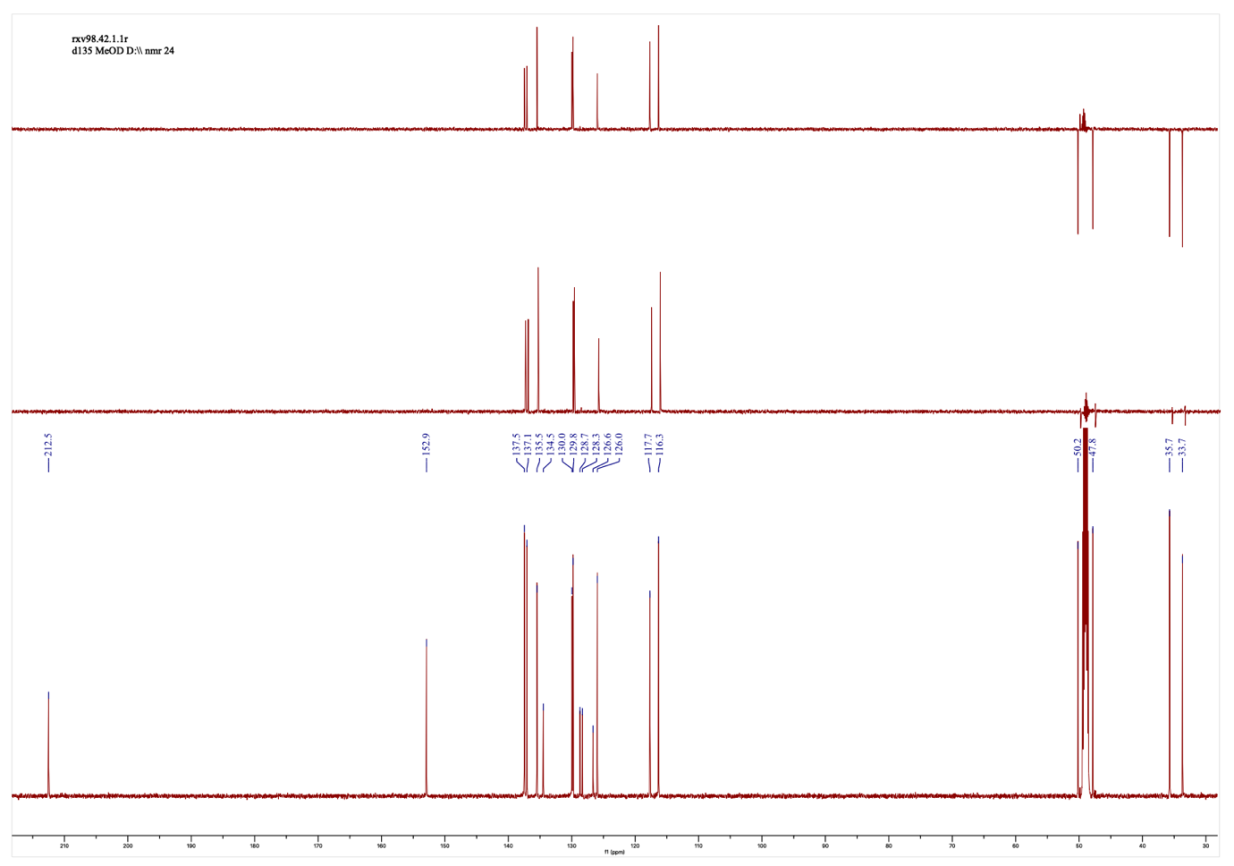


# **Figure S30.** ^13^C NMR and DEPT spectra of compound **4** (MeOD, 150 MHz)


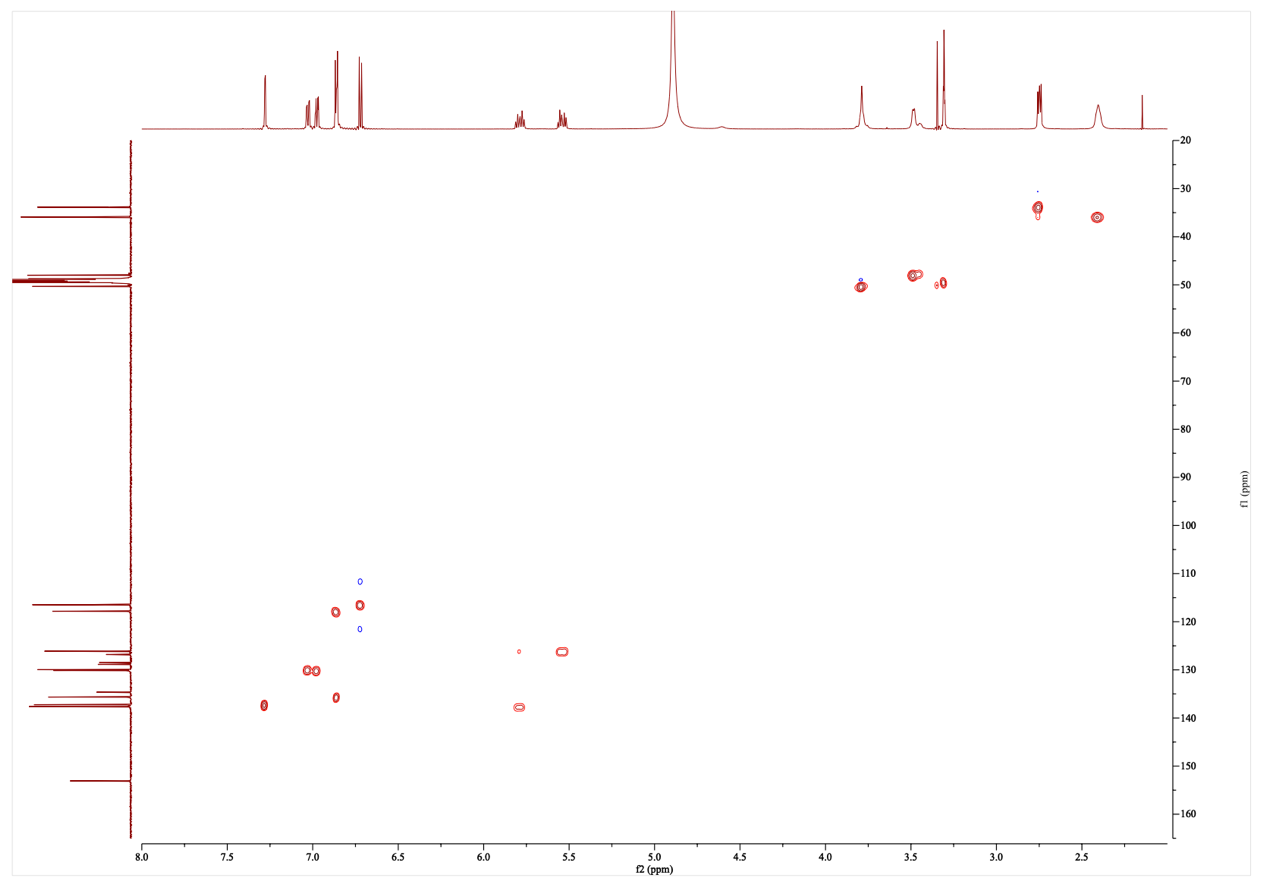


# **Figure S31.** HSQC spectrum of compound **4**


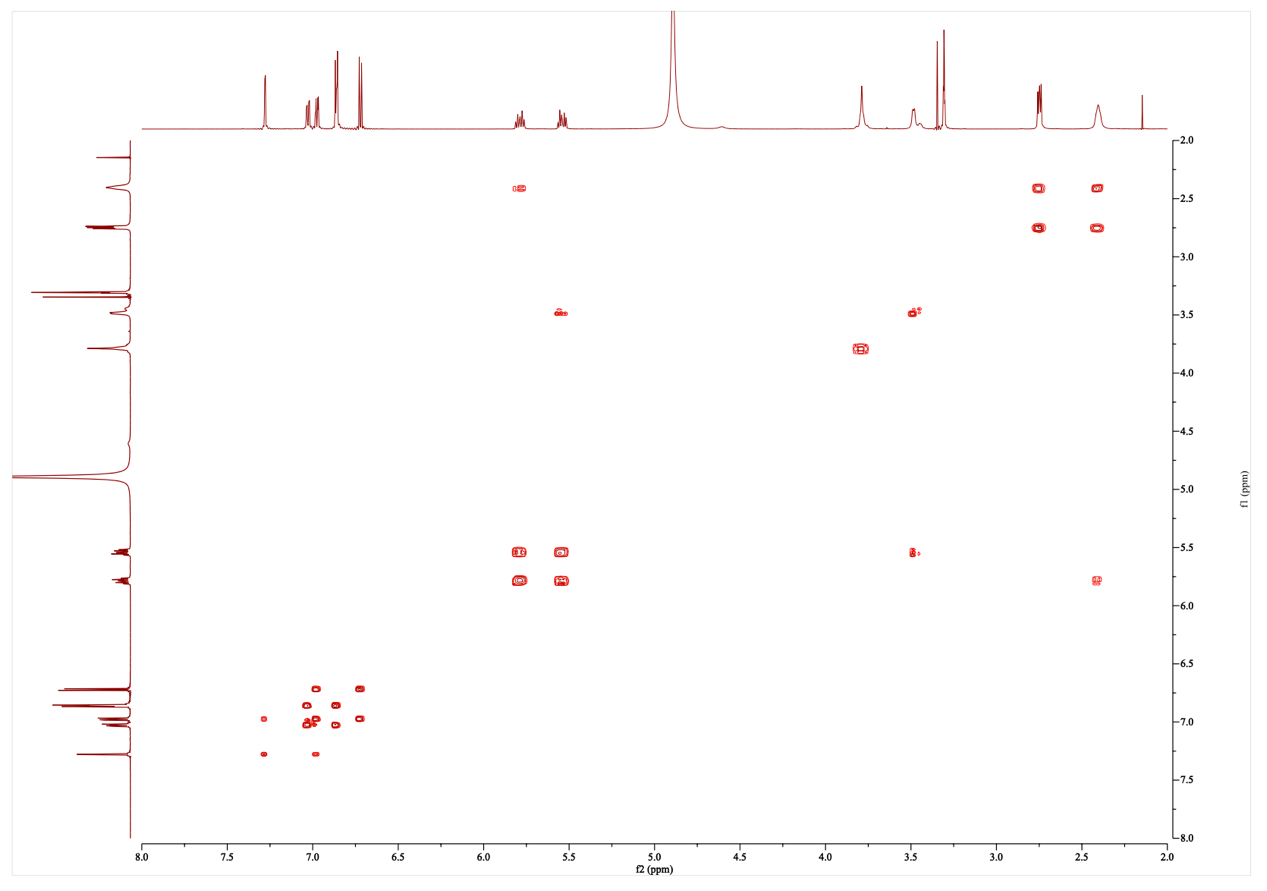


# **Figure S32.** ^1^H-^1^H COSY spectrum of compound **4**


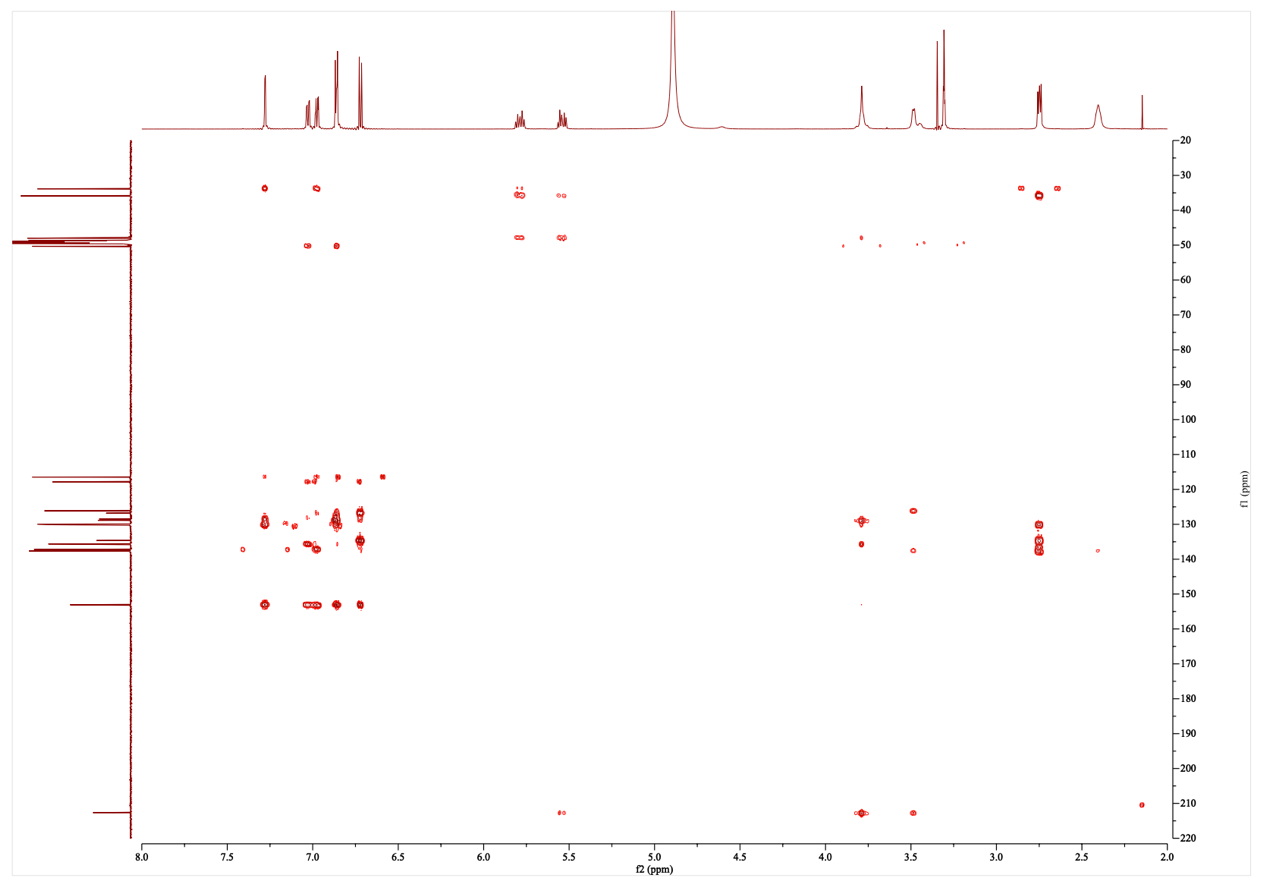


# **Figure S33.** HMBC spectrum of compound **4**


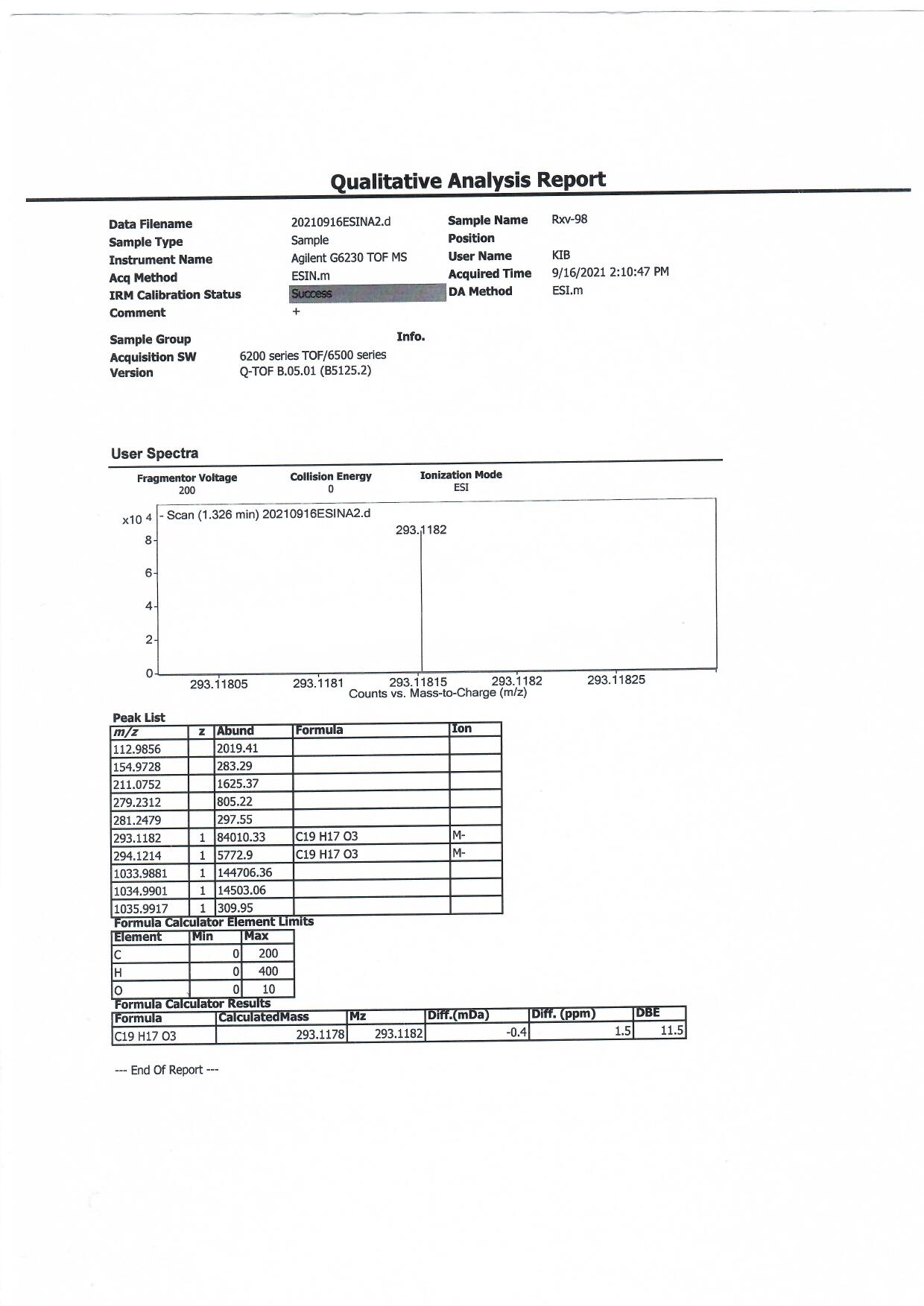


# **Figure S34.** HRESIMS spectrum of compound **4**

# **Figure S35.** IR spectrum of compound **4**


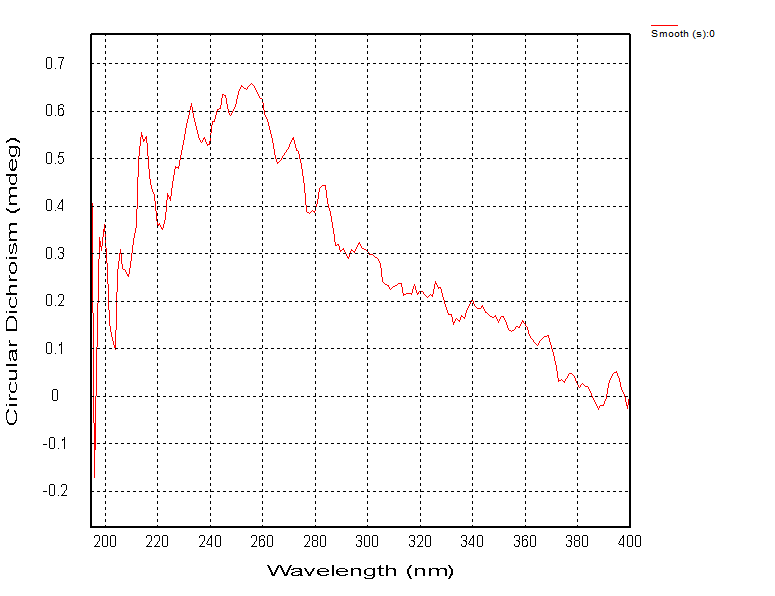


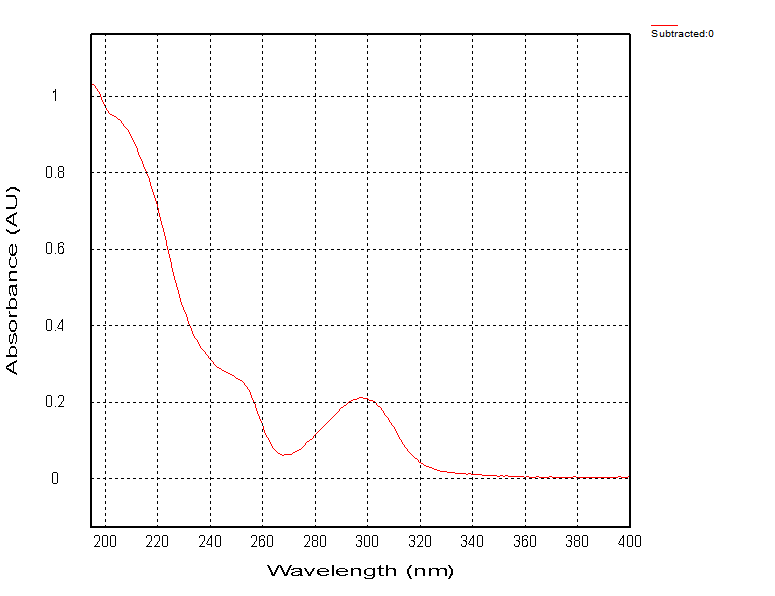


File: RXV-98-1mm(195-400)22040102.dsx

ProBinaryX

Attributes :

- Time Stamp :Fri Apr 01 13:17:33 2022

- File ID : {211ED230-94A8-409b-A938-173B4B411595}

- Is CFR Compliant : false

- Original data has not been modified.

Remarks:

- User: CD

- Date: 2022/04/01

- Instrument: 0547

- DetectorType: LAAPD

- DichOS Calibration Correction Curve: 0547/2

- HV (CDDC channel): 0 v

- Time per point: 1 s

- Description: Sample 1

- Concentration: 0.1045mg/mL MeOH

- Pathlength: 1 mm

- Temperature: 20℃

Settings:

- Time-per-point: 1s (25us x 40000)

- SE

- Wavelength: 195nm - 400nm

- Step Size: 1nm

- Bandwidth: 1nm

# **Figure S36.** ECD (top) and UV (bottom) spectra of compound **4**


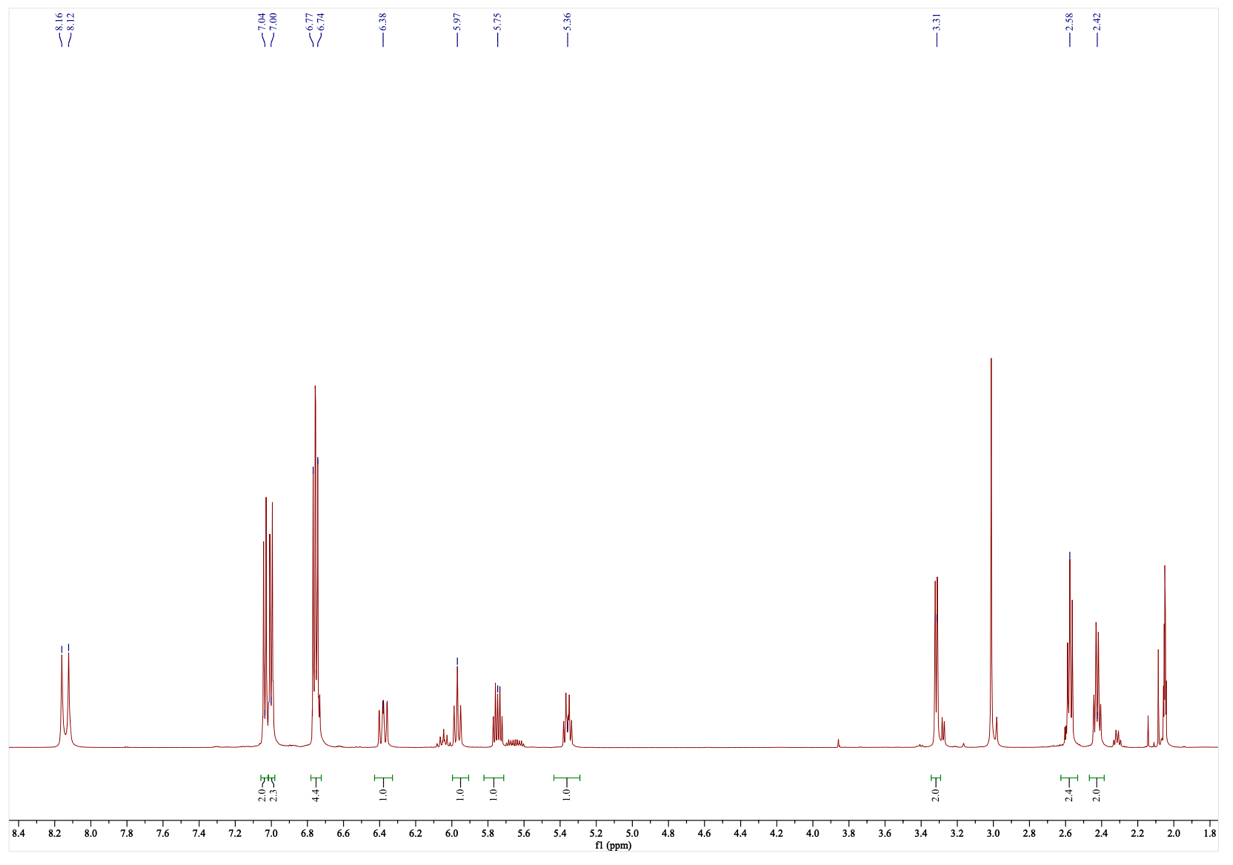


# **Figure S37.** ^1^H NMR spectrum of compound **5** (CD_3_COCD_3_, 600 MHz)


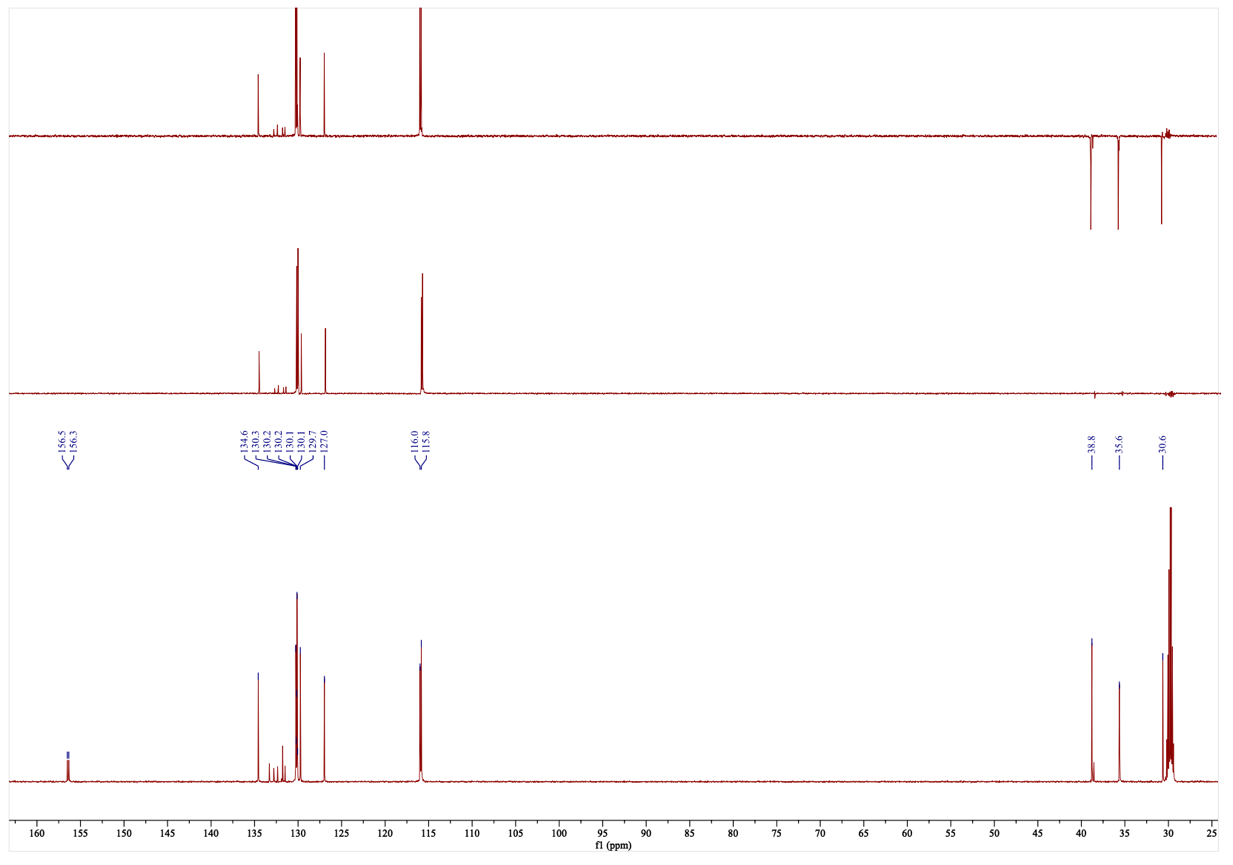


# **Figure S38.** ^13^C NMR and DEPT spectrum of compound **5** (CD_3_COCD_3_, 150 MHz)


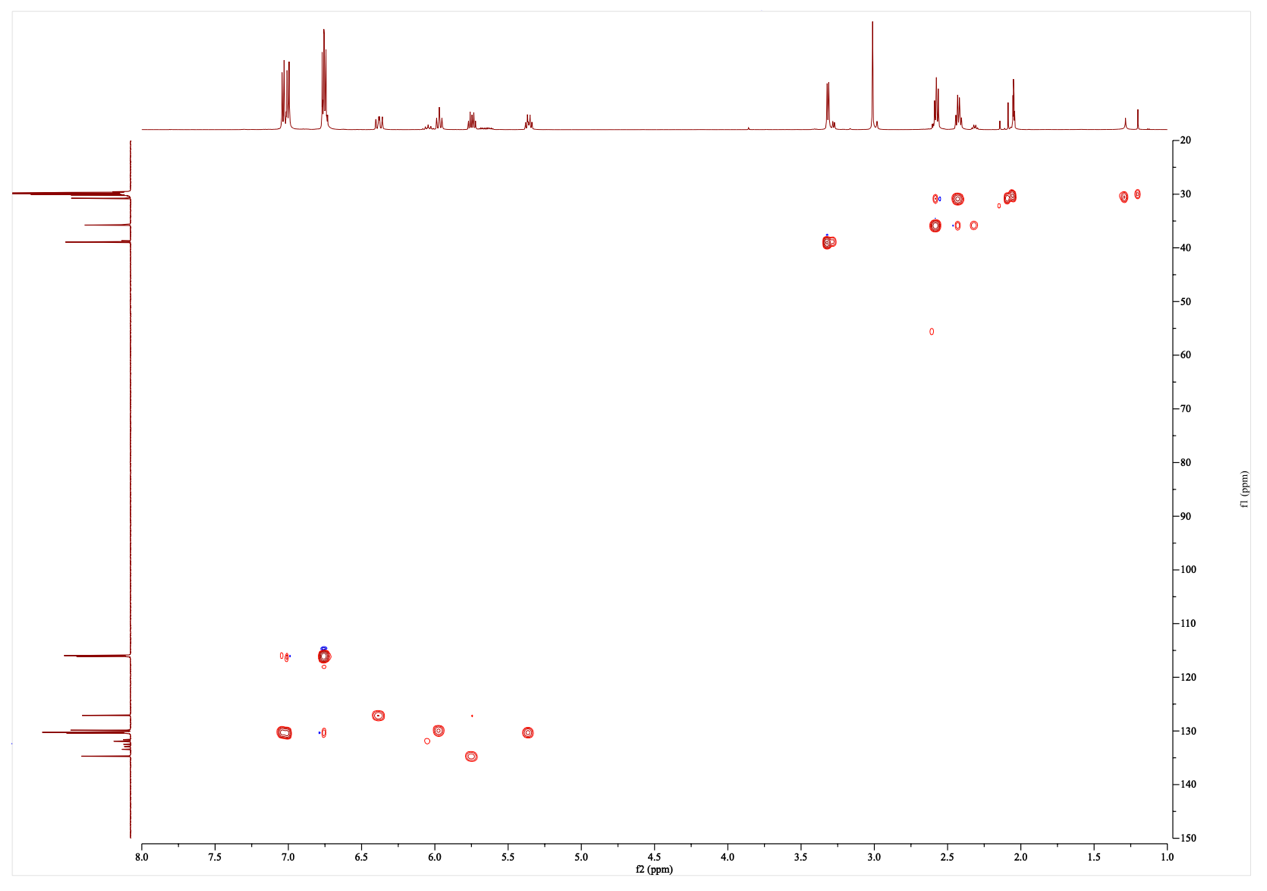


# **Figure S39.** HSQC spectrum of compound **5**


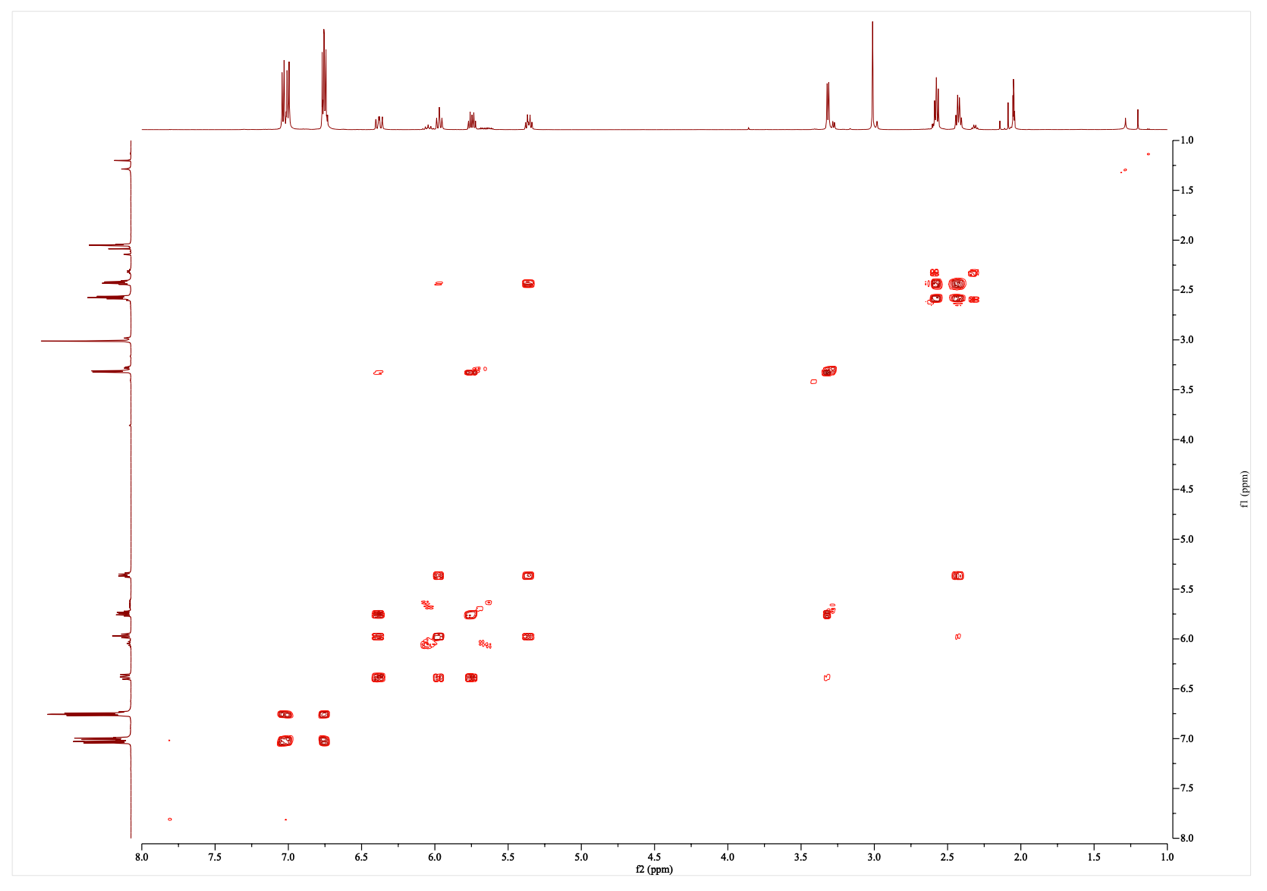


# **Figure S40**. ^1^H-^1^H COSY spectrum of compound **5**

**
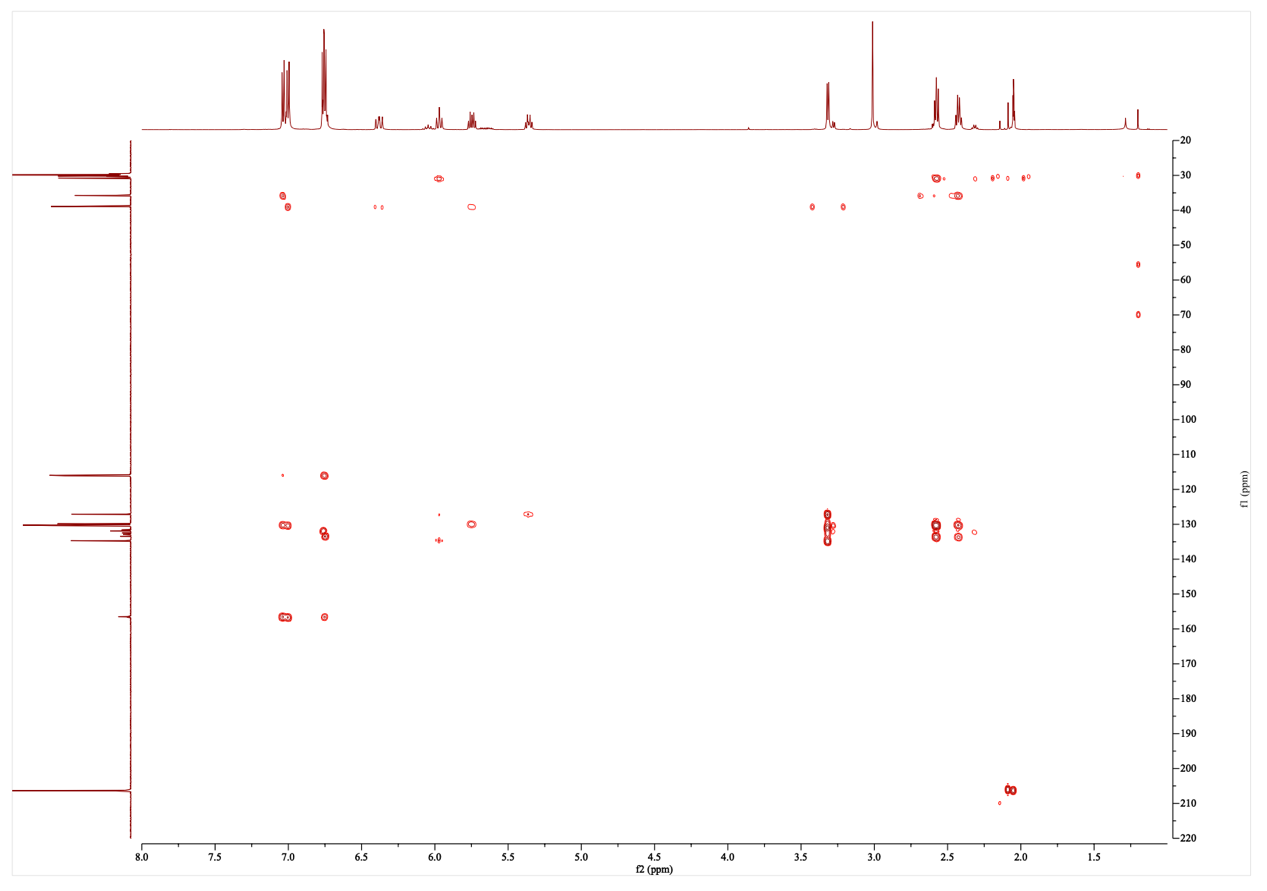
**

# **Figure S41.** HMBC spectrum of compound **5**


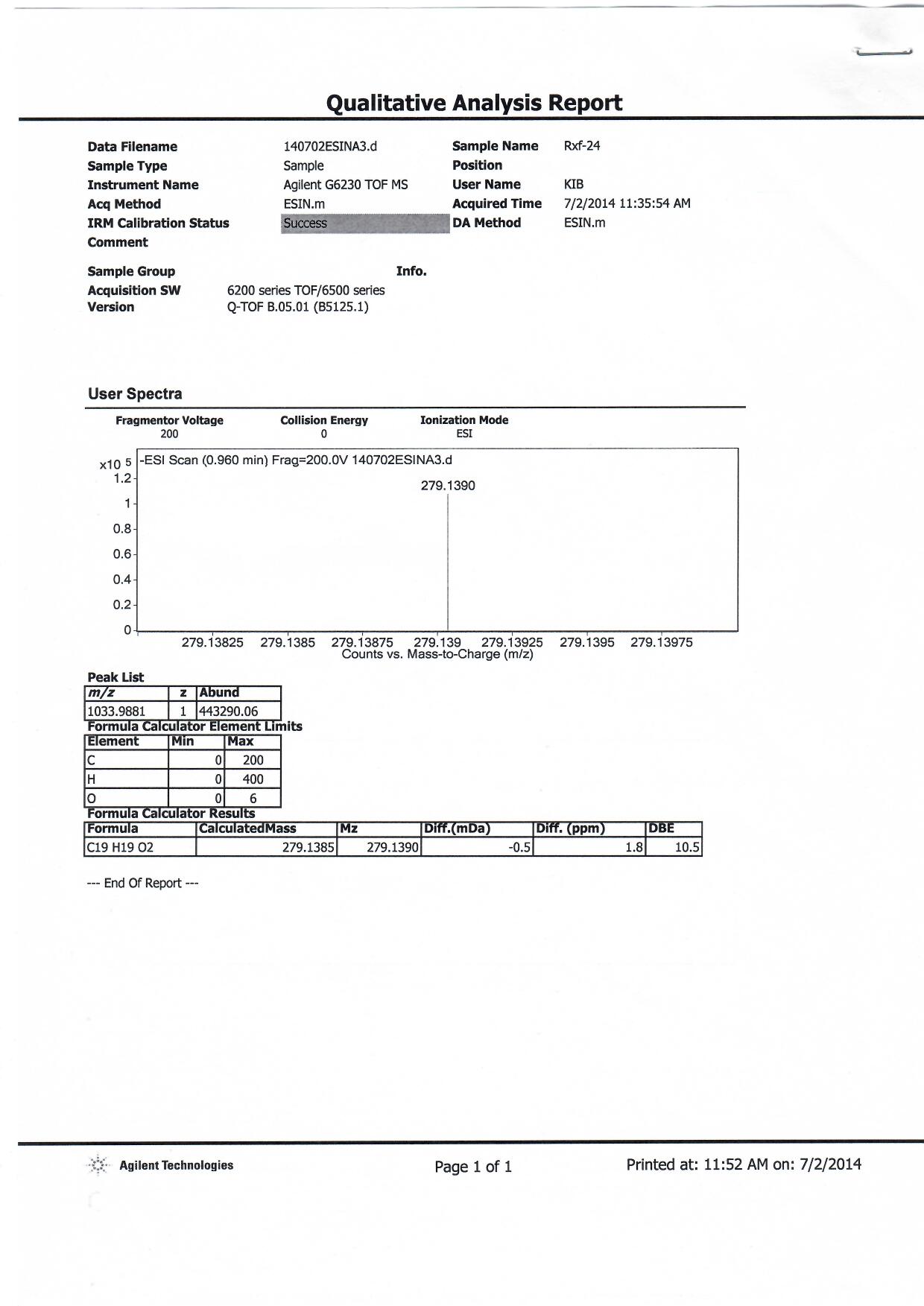


# **Figure S42.** HRESIMS spectrum of compound **5**


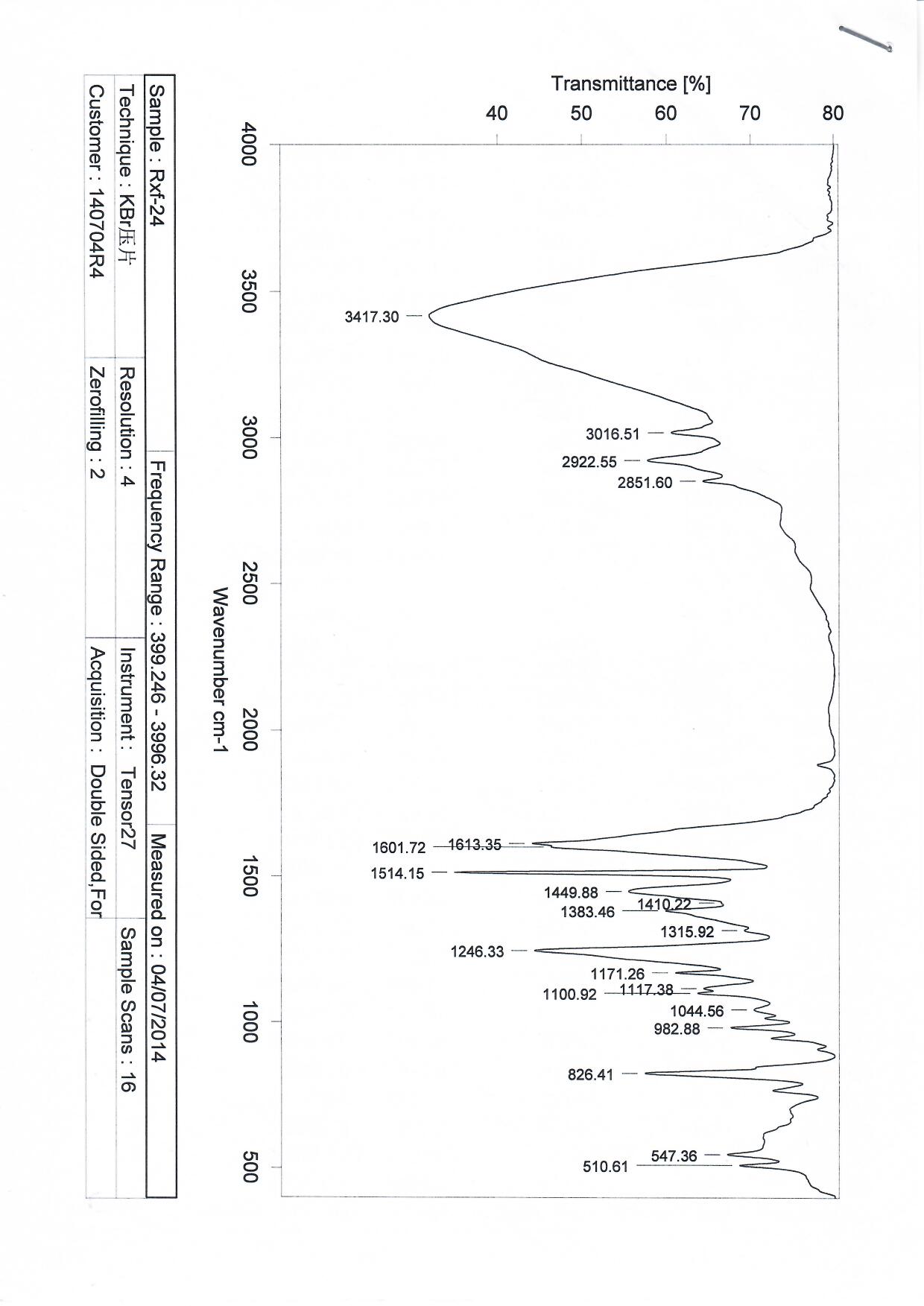


# **Figure S43.** IR spectrum of compound **5**


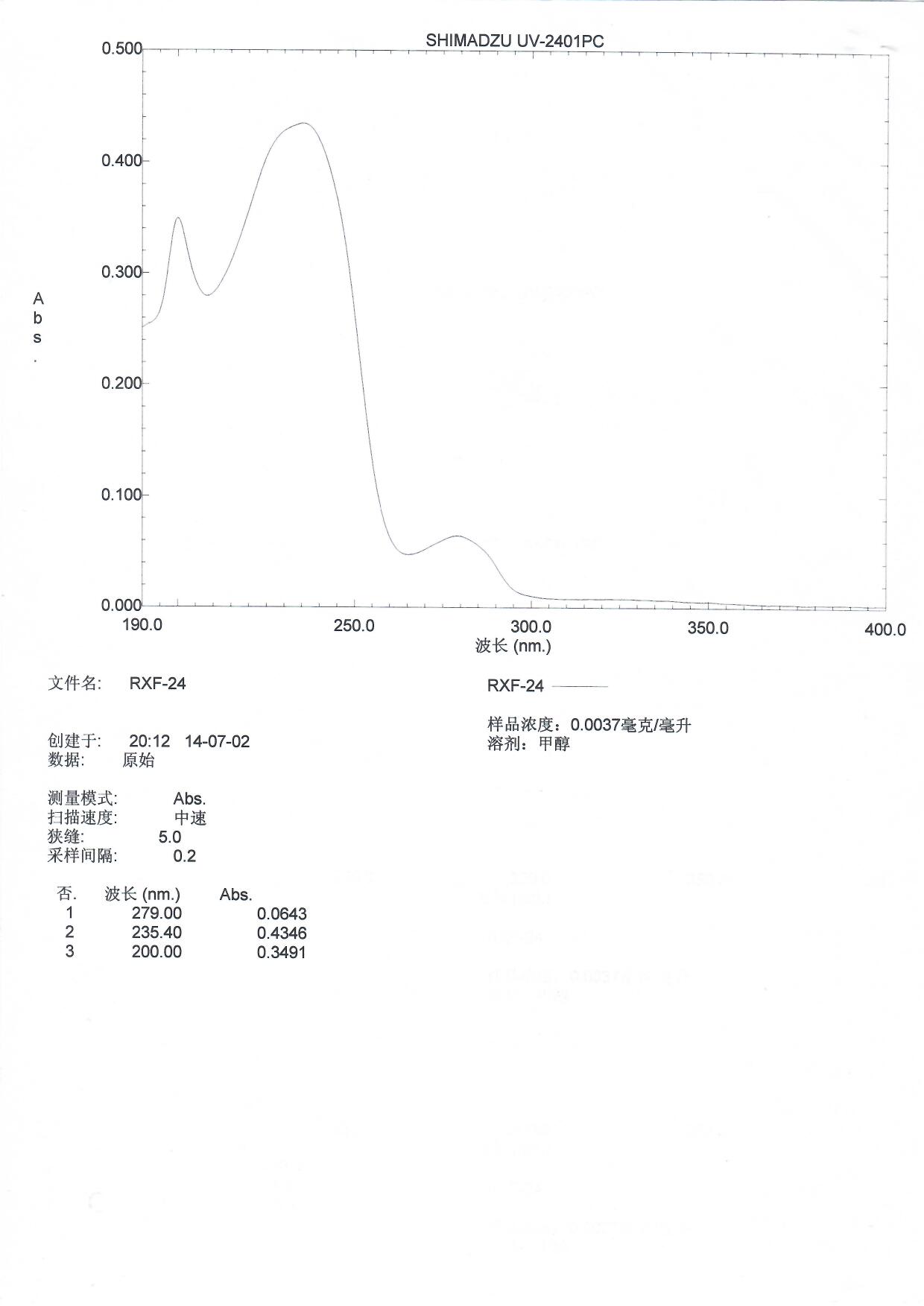


# **Figure S44.** UV spectrum of compound **5**


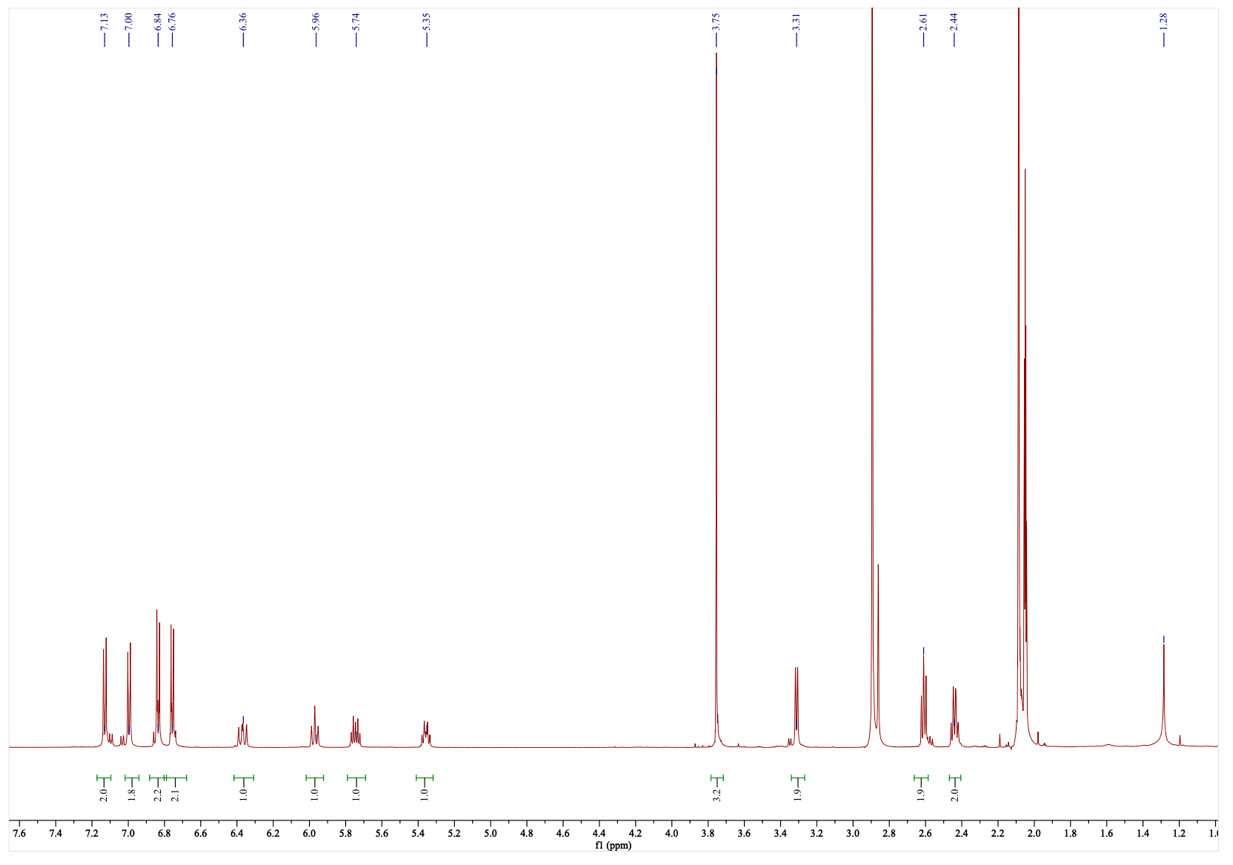


# **Figure S45.** ^1^H NMR spectrum of compound **6** (CD_3_COCD_3_, 600 MHz)


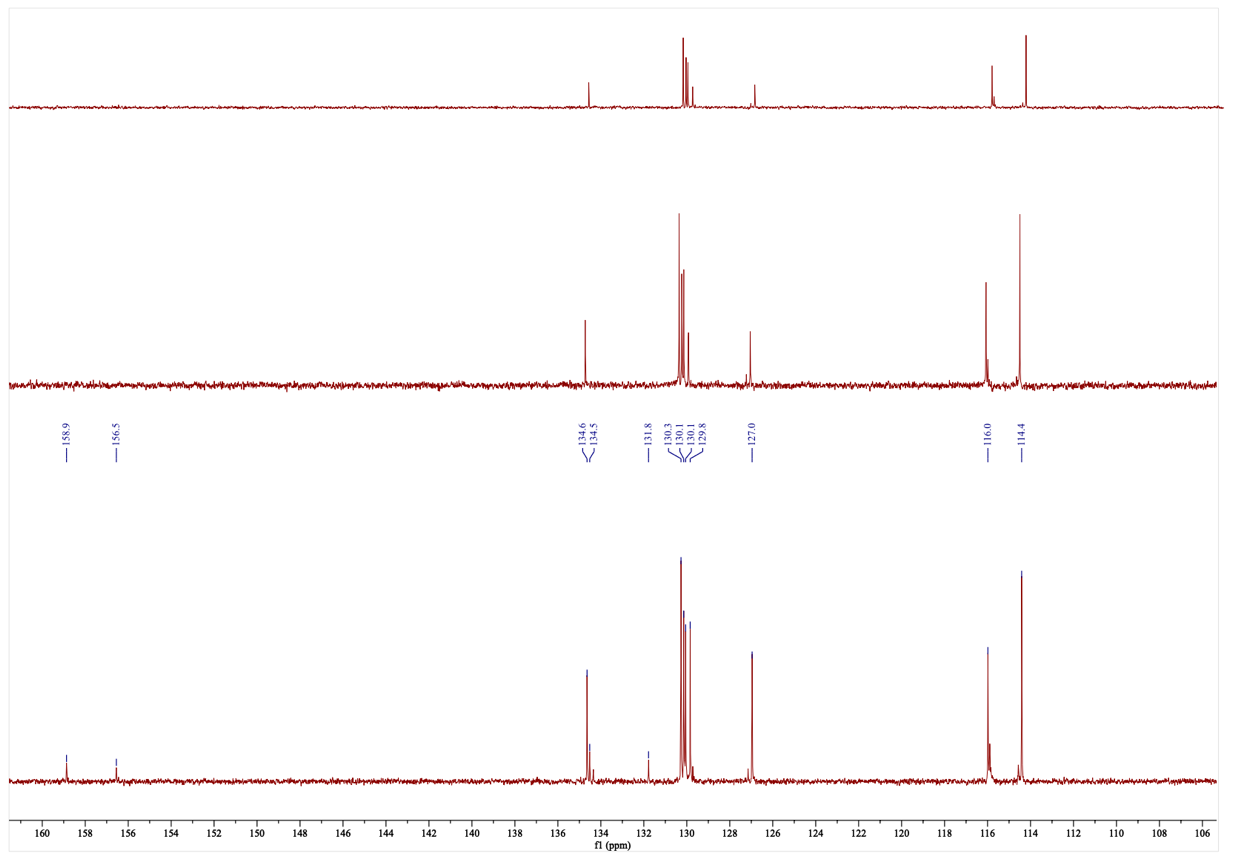


# **Figure S46.** ^13^C NMR and DEPT spectra of compound **6** (CD_3_COCD_3_, 150 MHz)


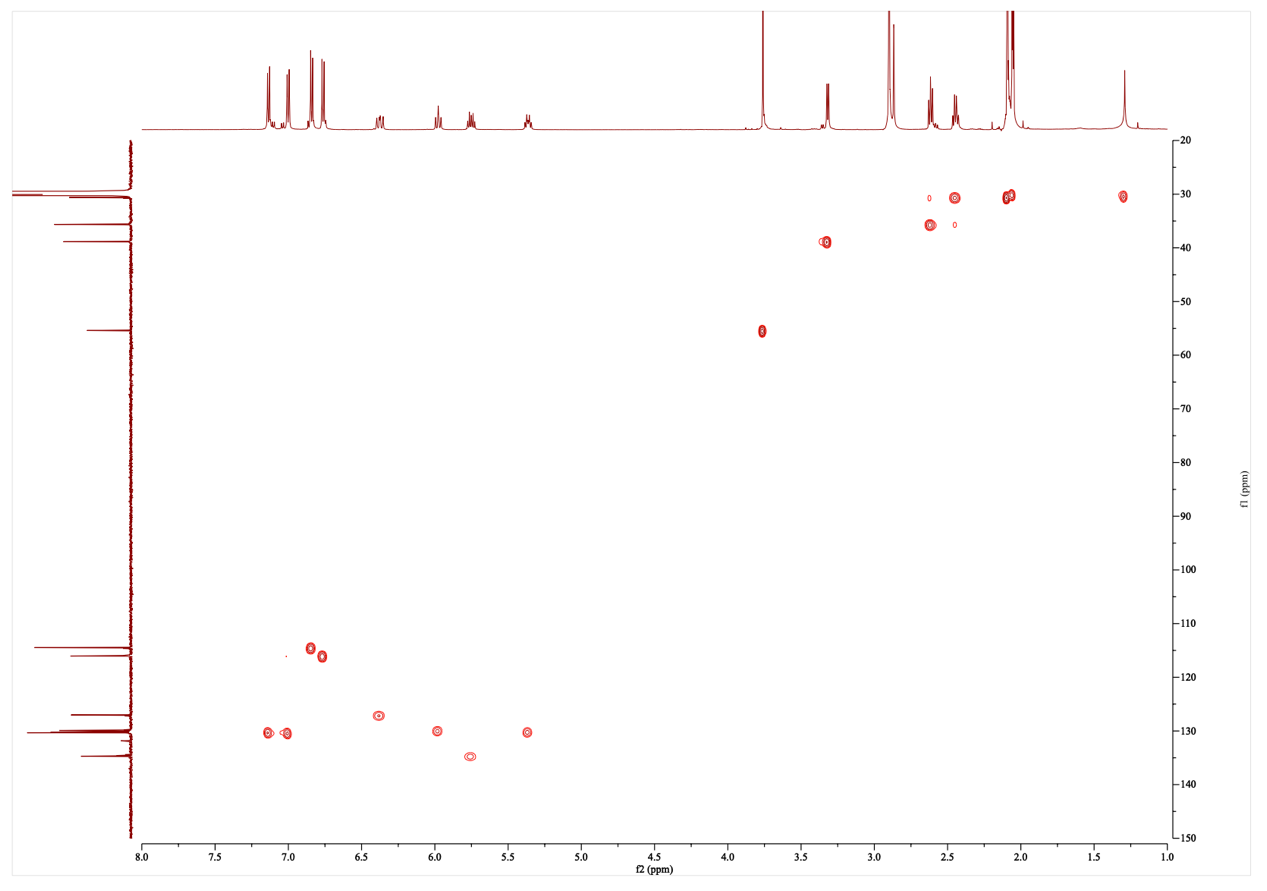


# **Figure S47.** HSQC spectrum of compound **6**


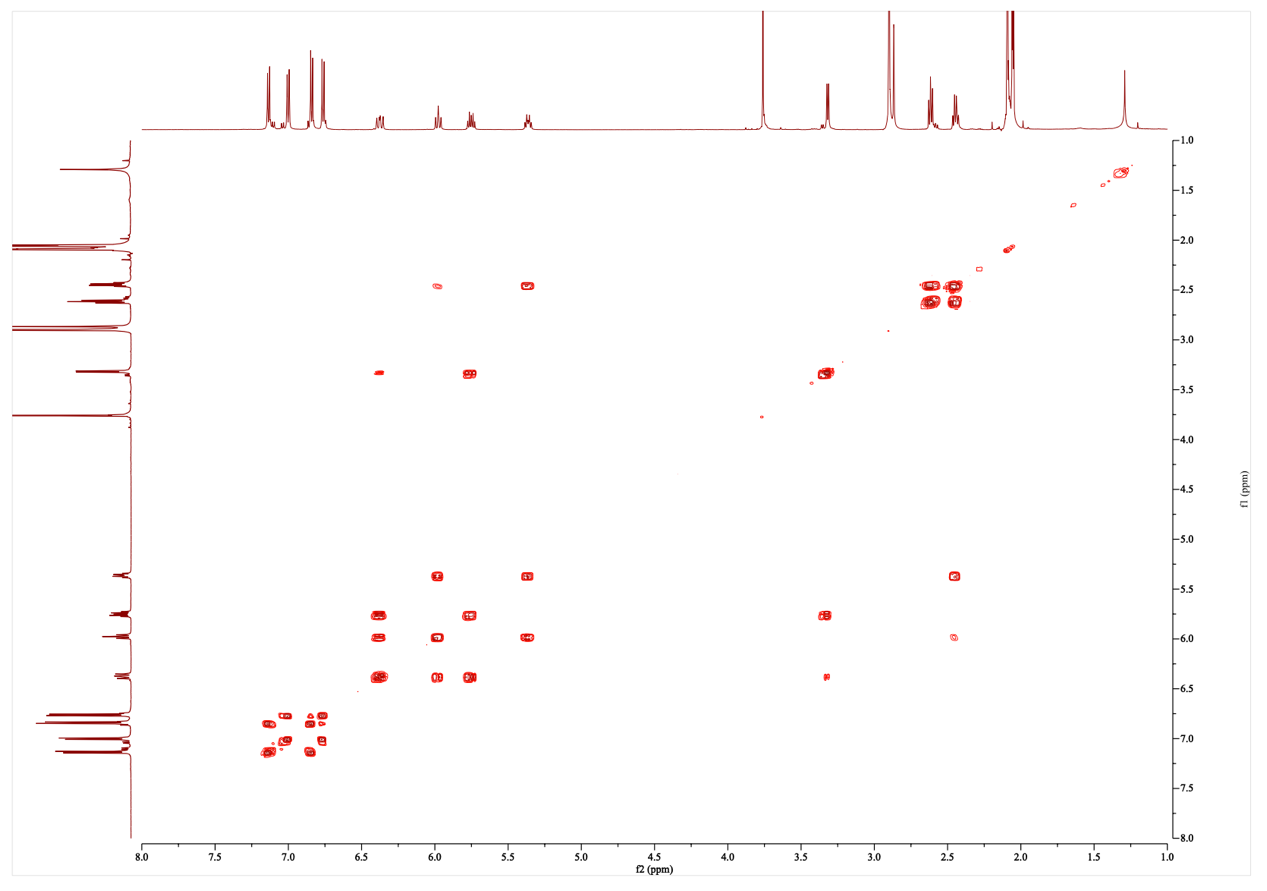


# **Figure S48.** ^1^H-^1^H COSY spectrum of compound **6**


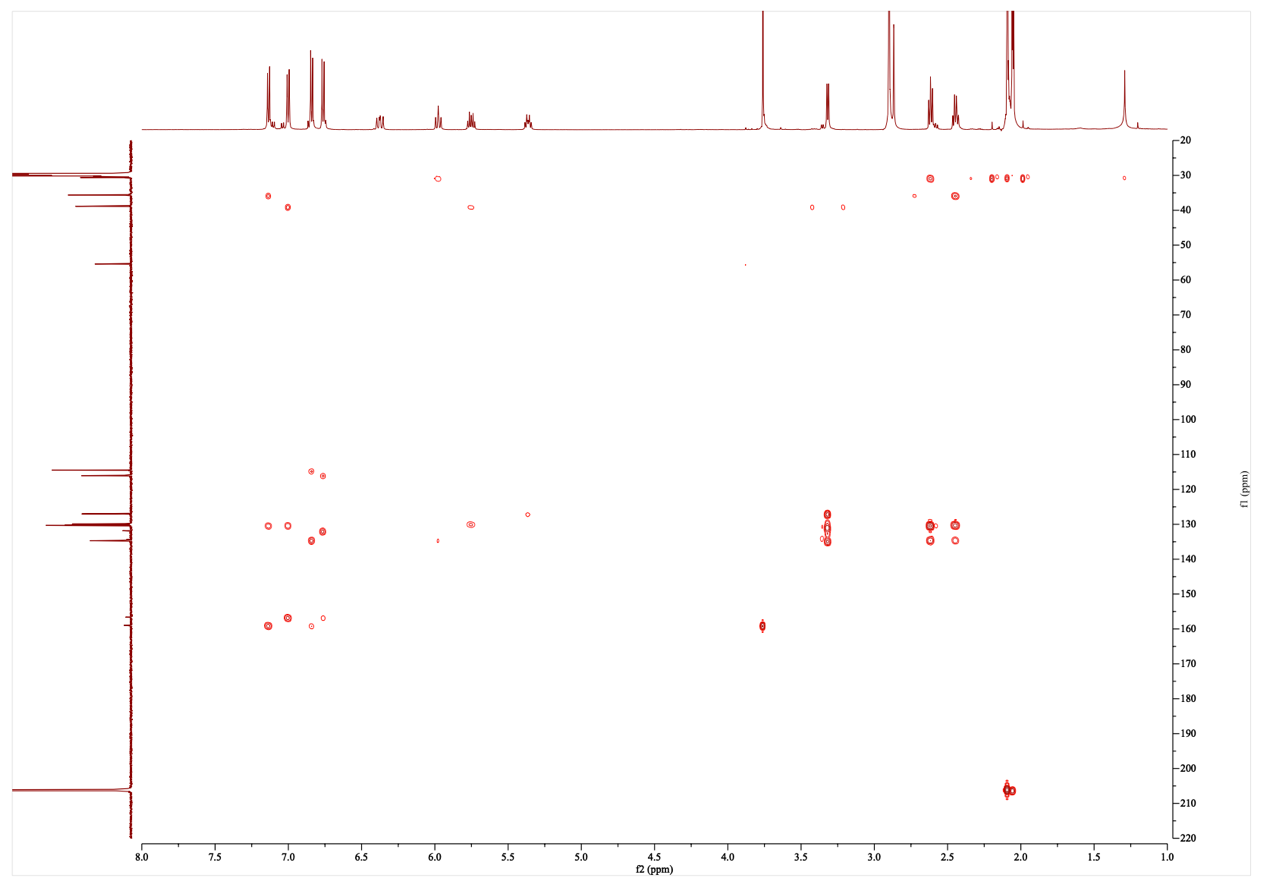


# **Figure S49.** HMBC spectrum of compound **6**


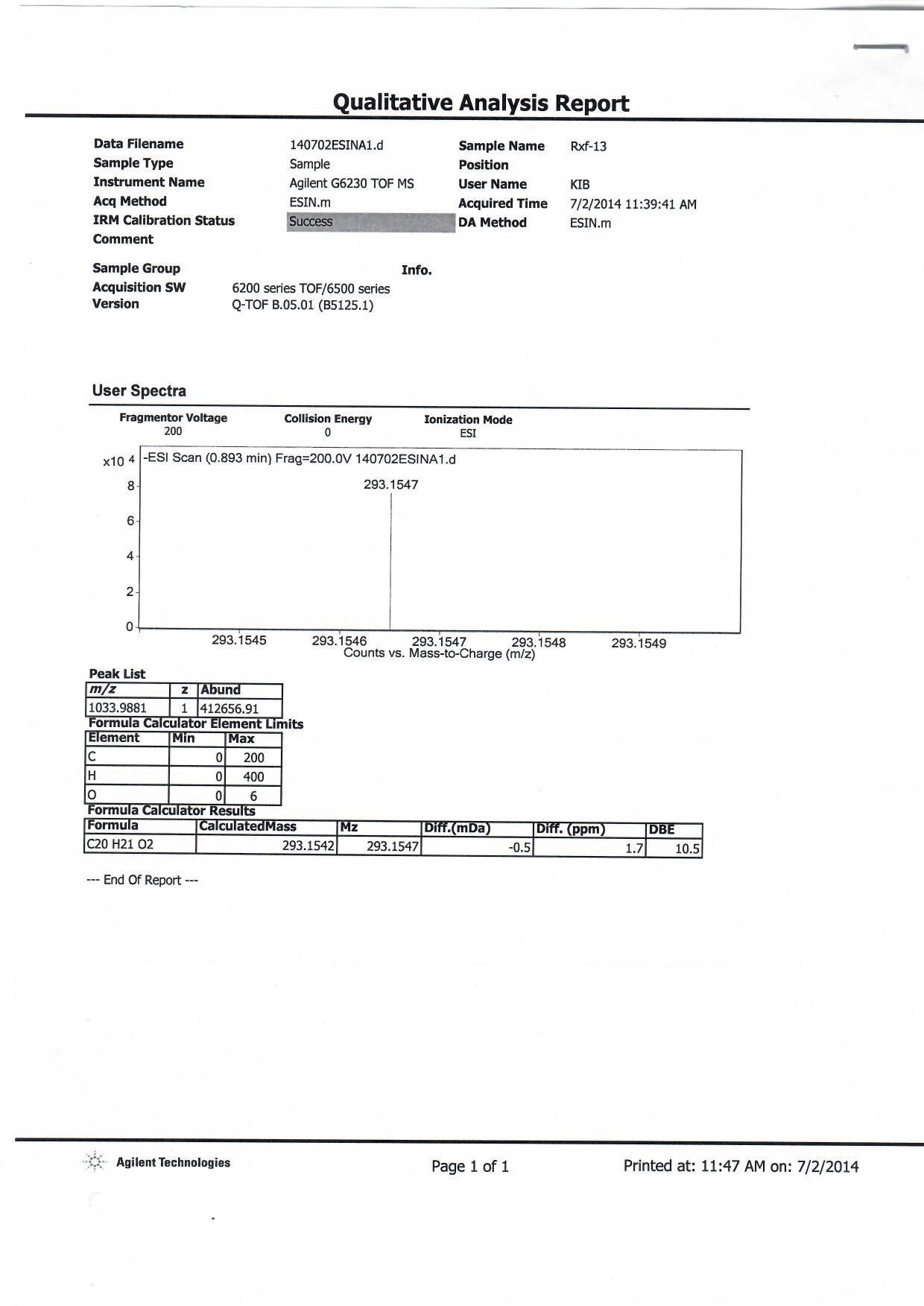


# **Figure S50.** HRESIMS spectrum of compound **6**

# **Figure S51.** IR spectrum of compound **6**

**Figure S52.** UV spectrum of compound **6**

**Calculated ECD data of compounds 2 and 4**

Conformational searching was performed with the Crest code (version 2.10) using the default iMTD-GC procedure^1^. The theoretical calculations were performed using Gaussian 16^2^. compounds **2** and (**+)-4** were optimized at M06-2X-D3/def2-SVP level, and energy of optimized conformers were evaluated at M06-2X-D3/def2-TZVP level. Subsequently, room-temperature (298.15 K) equilibrium populations were calculated according to Boltzmann distribution law:

$$p_{i}=\frac{n_{i}}{\sum_{j} n_{j}}=\frac{e^{-\Delta G_{i}/RT}}{\sum_{j} e^{-\Delta G_{j}/RT}}$$

where $P_{i}$ is the population of the $i^{th}$ conformer; $n_{i}$ the number of molecules in $i^{th}$ conformer; ΔG is the relative Gibbs free energy (kcal/mol); T is room temperature (298.15 K); R is the ideal gas constant (0.0019858995). The theoretical calculation of ECD was performed using time dependent Density Functional Theory (TDDFT) at Cam-B3LYP/TZVP level in methanol with IEFPCM solvent model. The calculated ECD curves were generated using the Multiwfn software^3^.

Reference:

[1] Pracht P, Bohle F, Grimme S. Phys. *Chem. Chem. Phys* 2020, **22**, 7169–7192.

[2] Frisch MJ, Trucks GW, Schlegel HB, Scuseria GE, Robb MA, Cheeseman JR, *et al*. Gaussian, Inc., Wallingford CT, 2016.

[3] Lu T, Chen FW, Multiwfn: A Multifunctional Wavefunction Analyzer. *J. Comput. Chem* 2012, **33**, 580–592.

| **** | | | |
| --- | --- | --- | --- |
| **2-1** | **2-2** | **2-3** | **2-4** |

# **Figure S53.** Optimized geometries of 4 dominate conformers of **2** at the M06-2X-D3/def2-SVP level of theory in the gas phase.

# **Table S1.** Conformational analysis of the M06-2X-D3/def2-SVP optimized conformers of **2** in the gas phase (T=298.15 K)

| Conformer | E (Hartree)^a^ | C (Hartree)^b^ | G (kcal/mol)^c^ | ΔG (kcal/mol)^d^ | Population^e^ |
| --- | --- | --- | --- | --- | --- |
| Rxv48-1 | -960.460673 | 0.284542 | -602510.522308 | 0.0 | 79.17% |
| Rxv48-2 | -960.458179 | 0.283898 | -602509.361498 | 1.16081 | 11.15% |
| Rxv48-3 | -960.458045 | 0.28427 | -602509.043789 | 1.478519 | 6.52% |
| Rxv48-4 | -960.458593 | 0.285497 | -602508.617448 | 1.90486 | 3.17% |

^a^Electronic energy obtained at M06-2X-D3/def2-SVP level of theory; ^b^Thermal correction to Gibbs free energy obtained at M06-2X-D3/def2-TZVP level of theory; ^c^Gibbs free energy (E + C); ^d^The relative Gibbs free energy; ^e^The Boltzmann distribution of each conformer.

# **Table S2.** Atomic coordinates (Å) of **2-1** obtained at the M06-2X-D3/def2-SVP level of theory in the gas phase.

| C | -0.093392 | -2.199130 | -1.124600 | O | 4.628912 | -1.492767 | 0.324049 |
| --- | --- | --- | --- | --- | --- | --- | --- |
| C | -1.474673 | -2.011910 | -1.135906 | O | -5.130640 | 0.458035 | -0.651149 |
| C | -2.166685 | -1.651507 | 0.026660 | H | 0.462666 | -2.407858 | -2.039440 |
| C | -1.452601 | -1.576279 | 1.228776 | H | -2.021647 | -2.105053 | -2.077252 |
| C | -0.073042 | -1.760088 | 1.255572 | H | -1.979918 | -1.318833 | 2.149646 |
| C | 0.601861 | -2.021805 | 0.066552 | H | 0.500811 | -1.626758 | 2.173741 |
| C | -3.611244 | -1.236664 | -0.045685 | H | -4.140872 | -1.455076 | 0.893999 |
| C | -3.764506 | 0.265787 | -0.348809 | H | -4.134345 | -1.773009 | -0.850722 |
| C | -3.368819 | 1.124867 | 0.821096 | H | -3.133160 | 0.496778 | -1.227233 |
| C | -2.295915 | 1.924219 | 0.945040 | H | -4.066261 | 1.053477 | 1.663735 |
| C | -1.228186 | 2.144450 | -0.029989 | H | -2.171093 | 2.456240 | 1.894246 |
| C | -0.080351 | 2.769367 | 0.280433 | H | -1.355921 | 1.741770 | -1.041378 |
| C | 1.109971 | 2.879894 | -0.633465 | H | 0.053080 | 3.137903 | 1.304345 |
| C | 2.103481 | 1.752941 | -0.378583 | H | 1.614054 | 3.847776 | -0.499385 |
| C | 3.473945 | 1.943974 | -0.213491 | H | 0.770885 | 2.831033 | -1.681091 |
| C | 4.323188 | 0.853941 | 0.011438 | H | 3.895332 | 2.950320 | -0.256191 |
| C | 3.817436 | -0.441009 | 0.087776 | H | 5.396444 | 0.991353 | 0.146345 |
| C | 2.434932 | -0.630160 | -0.074432 | H | 0.531929 | 0.292303 | -0.421745 |
| C | 1.601930 | 0.450577 | -0.312187 | H | 4.080873 | -2.289071 | 0.356807 |
| O | 1.981534 | -1.929824 | 0.039687 | H | -5.285279 | 1.404358 | -0.746304 |

# **Table S3.** Atomic coordinates (Å) of **2-2** obtained at the M06-2X-D3/def2-SVP level of theory in the gas phase.

| C | -0.095058 | -2.196298 | -1.123053 | O | 4.629291 | -1.497097 | 0.324678 |
| --- | --- | --- | --- | --- | --- | --- | --- |
| C | -1.475932 | -2.007349 | -1.133936 | O | -5.100227 | 0.552068 | -0.746795 |
| C | -2.167239 | -1.647909 | 0.029511 | H | 0.460354 | -2.405006 | -2.038272 |
| C | -1.452405 | -1.572175 | 1.231131 | H | -2.023056 | -2.099039 | -2.075324 |
| C | -0.073080 | -1.757412 | 1.257091 | H | -1.978148 | -1.313485 | 2.152594 |
| C | 0.601120 | -2.020300 | 0.067814 | H | 0.501427 | -1.624311 | 2.174864 |
| C | -3.610859 | -1.230138 | -0.047860 | H | -4.134991 | -1.455776 | 0.896004 |
| C | -3.772644 | 0.278322 | -0.356728 | H | -4.130188 | -1.769863 | -0.852928 |
| C | -3.369611 | 1.134692 | 0.814046 | H | -3.151163 | 0.510422 | -1.234282 |
| C | -2.292539 | 1.925057 | 0.944932 | H | -4.054994 | 1.067271 | 1.669770 |
| C | -1.226013 | 2.146982 | -0.030503 | H | -2.163697 | 2.448880 | 1.898014 |
| C | -0.075596 | 2.765533 | 0.282540 | H | -1.360241 | 1.755048 | -1.045237 |
| C | 1.114096 | 2.879134 | -0.631746 | H | 0.060322 | 3.126894 | 1.308677 |
| C | 2.107353 | 1.751527 | -0.379315 | H | 1.618144 | 3.846717 | -0.495287 |
| C | 3.478115 | 1.940862 | -0.214124 | H | 0.774523 | 2.833220 | -1.679300 |
| C | 4.326073 | 0.849902 | 0.010844 | H | 3.900554 | 2.946767 | -0.256641 |
| C | 3.818917 | -0.444549 | 0.087625 | H | 5.399484 | 0.986063 | 0.145848 |
| C | 2.436339 | -0.631862 | -0.074805 | H | 0.534459 | 0.293297 | -0.423029 |
| C | 1.604674 | 0.449687 | -0.313217 | H | 4.080675 | -2.292988 | 0.356428 |
| O | 1.980542 | -1.931097 | 0.040294 | H | -5.664576 | 0.472667 | 0.031206 |

# **Table S4.** Atomic coordinates (Å) of **2-3** obtained at the M06-2X-D3/def2-SVP level of theory in the gas phase.

| C | -0.094382 | -2.201398 | -1.115704 | O | 4.633920 | -1.496373 | 0.332242 |
| --- | --- | --- | --- | --- | --- | --- | --- |
| C | -1.474577 | -2.006014 | -1.125785 | O | -5.152982 | 0.564049 | -0.580865 |
| C | -2.162409 | -1.630653 | 0.034296 | H | 0.458583 | -2.422328 | -2.029489 |
| C | -1.445184 | -1.544242 | 1.234113 | H | -2.022579 | -2.107928 | -2.065790 |
| C | -0.067178 | -1.736596 | 1.259469 | H | -1.968853 | -1.272431 | 2.152762 |
| C | 0.604227 | -2.016265 | 0.072028 | H | 0.509465 | -1.595963 | 2.174773 |
| C | -3.607524 | -1.218700 | -0.036899 | H | -4.136480 | -1.438416 | 0.902854 |
| C | -3.789894 | 0.288337 | -0.346899 | H | -4.114749 | -1.784239 | -0.836450 |
| C | -3.382236 | 1.147622 | 0.815251 | H | -3.176990 | 0.529798 | -1.235615 |
| C | -2.292524 | 1.920468 | 0.941794 | H | -4.089785 | 1.100628 | 1.649939 |
| C | -1.222621 | 2.131707 | -0.032842 | H | -2.161719 | 2.449325 | 1.891709 |
| C | -0.074059 | 2.756938 | 0.274030 | H | -1.355776 | 1.732343 | -1.045044 |
| C | 1.114022 | 2.872126 | -0.642913 | H | 0.061373 | 3.125717 | 1.297545 |
| C | 2.111286 | 1.748349 | -0.389966 | H | 1.614916 | 3.841859 | -0.510083 |
| C | 3.481324 | 1.939364 | -0.220870 | H | 0.772849 | 2.822413 | -1.689826 |
| C | 4.329458 | 0.849466 | 0.009333 | H | 3.903013 | 2.945593 | -0.263255 |
| C | 3.823324 | -0.445350 | 0.088481 | H | 5.402236 | 0.986979 | 0.148020 |
| C | 2.441593 | -0.633986 | -0.078315 | H | 0.540692 | 0.289694 | -0.436157 |
| C | 1.610469 | 0.446151 | -0.323584 | H | 4.085762 | -2.292385 | 0.367417 |
| O | 1.983676 | -1.932587 | 0.041137 | H | -5.457552 | 0.004690 | -1.303514 |

# **Table S5.** Atomic coordinates (Å) of **2-4** obtained at the M06-2X-D3/def2-SVP level of theory in the gas phase.

| C | 0.227667 | -1.679603 | 1.299862 | O | -4.444313 | -1.657815 | 0.288350 |
| --- | --- | --- | --- | --- | --- | --- | --- |
| C | 1.603205 | -1.475703 | 1.310203 | O | 3.193331 | 0.602837 | -1.511616 |
| C | 2.363145 | -1.618149 | 0.140338 | H | -0.379005 | -1.502919 | 2.189238 |
| C | 1.716303 | -2.064734 | -1.020067 | H | 2.092304 | -1.150938 | 2.230653 |
| C | 0.334300 | -2.261186 | -1.047015 | H | 2.298363 | -2.212261 | -1.932830 |
| C | -0.404330 | -2.018227 | 0.104353 | H | -0.185374 | -2.536870 | -1.965333 |
| C | 3.790845 | -1.143886 | 0.079114 | H | 4.344012 | -1.699837 | -0.691283 |
| C | 3.850490 | 0.367277 | -0.296073 | H | 4.299617 | -1.299382 | 1.041673 |
| C | 3.393804 | 1.226470 | 0.857104 | H | 4.911325 | 0.605048 | -0.480911 |
| C | 2.324107 | 2.030665 | 0.976090 | H | 4.037260 | 1.133501 | 1.739243 |
| C | 1.263404 | 2.319646 | 0.004516 | H | 2.182316 | 2.506891 | 1.952763 |
| C | 0.060698 | 2.784214 | 0.378560 | H | 1.456022 | 2.119977 | -1.052517 |
| C | -1.128194 | 2.898916 | -0.534960 | H | -0.123817 | 2.977891 | 1.442277 |
| C | -2.063919 | 1.711913 | -0.340487 | H | -0.783234 | 2.929994 | -1.581270 |
| C | -3.443959 | 1.834344 | -0.186118 | H | -1.683147 | 3.829662 | -0.345998 |
| C | -4.245187 | 0.703980 | 0.009113 | H | -3.909639 | 2.821573 | -0.211188 |
| C | -3.680314 | -0.566805 | 0.072923 | H | -5.324840 | 0.789869 | 0.135251 |
| C | -2.289275 | -0.688703 | -0.077775 | H | -0.425216 | 0.324488 | -0.396157 |
| C | -1.503293 | 0.431579 | -0.295811 | H | -3.860997 | -2.428780 | 0.315115 |
| O | -1.781941 | -1.970010 | 0.029554 | H | 2.300425 | 0.239941 | -1.442247 |

| **** | | | |
| --- | --- | --- | --- |
| **+3-1** | **+3-2** | **+3-3** | **+3-4** |

# **Figure S54.** Optimized geometries of 4 dominate conformers of **+3** at the M06-2X-D3/def2-SVP level of theory in the gas phase.

# **Table S6.** Conformational analysis of the M06-2X-D3/def2-SVP optimized conformers of **+3** in the gas phase (T=298.15 K)

| Conformer | E (Hartree)^a^ | C (Hartree)^b^ | G (kcal/mol)^c^ | ΔG (kcal/mol)^d^ | Population^e^ |
| --- | --- | --- | --- | --- | --- |
| Rxv44-1 | -1076.205617 | 0.337982 | -675106.940668 | 0.0 | 82.12% |
| Rxv44-2 | -1076.203885 | 0.338004 | -675105.840302 | 1.100365 | 12.80% |
| Rxv44-3 | -1076.201867 | 0.337514 | -675104.88152 | 2.059148 | 2.54% |
| Rxv44-4 | -1076.201866 | 0.337514 | -675104.881194 | 2.059474 | 2.53% |

^a^Electronic energy obtained at M06-2X-D3/def2-SVP level of theory; ^b^Thermal correction to Gibbs free energy obtained at M06-2X-D3/def2-TZVP level of theory; ^c^Gibbs free energy (E + C); ^d^The relative Gibbs free energy; ^e^The Boltzmann distribution of each conformer.

# **Table S7.** Atomic coordinates (Å) of **+3-1** obtained at the M06-2X-D3/def2-SVP level of theory in the gas phase.

| C | 3.141720 | 1.929469 | -0.676150 | C | 3.728630 | -1.869909 | 1.806386 |
| --- | --- | --- | --- | --- | --- | --- | --- |
| C | 3.130472 | 0.552760 | -0.440095 | H | 4.015134 | 2.426403 | -1.099364 |
| C | 2.003378 | -0.062155 | 0.106906 | H | 4.006847 | -0.053428 | -0.676628 |
| C | 0.886898 | 0.720193 | 0.408650 | H | -0.006203 | 0.254779 | 0.826464 |
| C | 0.882914 | 2.081777 | 0.140544 | H | 1.266538 | -1.758599 | 1.194507 |
| C | 2.018461 | 2.707181 | -0.397575 | H | 2.039244 | -2.143248 | -1.718138 |
| C | 1.918072 | -1.557558 | 0.320896 | H | -3.564815 | 0.558285 | 2.332792 |
| C | 1.337468 | -2.328240 | -0.879270 | H | -3.277107 | 0.493594 | -1.956464 |
| C | -3.104482 | 0.912109 | 1.406843 | H | -1.377937 | 2.109621 | -1.848230 |
| C | -3.527295 | 0.383997 | 0.183293 | H | -1.665755 | 2.186753 | 2.427483 |
| C | -2.943459 | 0.877593 | -0.989844 | H | 1.139546 | 4.373263 | -0.412139 |
| C | -1.889714 | 1.782664 | -0.941391 | H | -0.309036 | -2.474526 | -2.223595 |
| C | -1.414492 | 2.206414 | 0.297316 | H | 0.017468 | -0.832596 | -1.653757 |
| C | -2.050493 | 1.825311 | 1.472976 | H | -0.907789 | -1.827358 | 0.737291 |
| O | -0.214159 | 2.893318 | 0.355729 | H | -2.703926 | -2.411594 | -1.670602 |
| O | 2.013738 | 4.031584 | -0.647693 | H | -4.242768 | -2.990611 | 0.276390 |
| C | -0.049325 | -1.886269 | -1.330551 | H | -3.173568 | -2.103080 | 1.379396 |
| C | -1.155115 | -2.010766 | -0.316108 | H | -4.917400 | -0.887695 | -0.846953 |
| C | -2.436721 | -2.229597 | -0.621001 | H | -5.200735 | -0.780443 | 0.899154 |
| C | -3.574397 | -2.118376 | 0.353419 | H | 2.150746 | -3.905382 | -0.178747 |
| C | -4.414699 | -0.832141 | 0.130946 | H | 3.070245 | -2.231399 | 2.616387 |
| O | 1.288093 | -3.692016 | -0.558653 | H | 4.692411 | -2.389630 | 1.871004 |
| O | 3.178357 | -2.149341 | 0.545708 | H | 3.893716 | -0.788197 | 1.945880 |

# **Table S8.** Atomic coordinates (Å) of **+3-2** obtained at the M06-2X-D3/def2-SVP level of theory in the gas phase.

| C | -3.659899 | 0.980796 | -0.531186 | C | -1.412181 | -2.062715 | 2.793026 |
| --- | --- | --- | --- | --- | --- | --- | --- |
| C | -3.323579 | -0.351789 | -0.271552 | H | -4.641109 | 1.254694 | -0.919614 |
| C | -2.063411 | -0.683535 | 0.227787 | H | -4.056976 | -1.139661 | -0.457916 |
| C | -1.141193 | 0.340948 | 0.465381 | H | -0.151621 | 0.090123 | 0.847769 |
| C | -1.466020 | 1.660194 | 0.191542 | H | -2.520767 | -2.747565 | 0.584340 |
| C | -2.735917 | 1.998892 | -0.306862 | H | -0.593370 | -3.726215 | -0.500863 |
| C | -1.620055 | -2.117737 | 0.448813 | H | 2.839029 | 1.131777 | -2.163919 |
| C | -0.895407 | -2.705772 | -0.782642 | H | 3.353073 | 1.272694 | 2.102104 |
| C | 2.477680 | 1.421925 | -1.174793 | H | 1.116043 | 2.372956 | 2.327433 |
| C | 3.235915 | 1.094928 | -0.044207 | H | 0.606108 | 2.222056 | -1.925583 |
| C | 2.765631 | 1.499357 | 1.208816 | H | -2.261724 | 3.819932 | -0.370585 |
| C | 1.521781 | 2.117686 | 1.347684 | H | -0.016713 | -0.922357 | -1.576923 |
| C | 0.743737 | 2.327702 | 0.215410 | H | 0.647463 | -2.424552 | -2.220082 |
| C | 1.235018 | 2.033031 | -1.053794 | H | 1.291316 | -1.626067 | 0.699605 |
| O | -0.581196 | 2.707882 | 0.350041 | H | 2.999762 | -1.855620 | -1.841463 |
| O | -3.042525 | 3.283742 | -0.569934 | H | 3.612001 | -1.387243 | 1.161170 |
| C | 0.329468 | -1.934118 | -1.287080 | H | 4.783187 | -1.989212 | -0.028604 |
| C | 1.507363 | -1.773243 | -0.363854 | H | 5.194453 | 0.399950 | 0.548962 |
| C | 2.777192 | -1.710995 | -0.775594 | H | 4.829387 | 0.204960 | -1.174458 |
| C | 3.925283 | -1.311880 | 0.107923 | H | -2.082400 | -1.945255 | -2.103370 |
| C | 4.396837 | 0.142405 | -0.163982 | H | -2.242104 | -2.780677 | 2.920967 |
| O | -1.831008 | -2.836455 | -1.826577 | H | -0.669883 | -2.233763 | 3.582237 |
| O | -0.771237 | -2.248505 | 1.560104 | H | -1.816868 | -1.040749 | 2.894704 |

# **Table S9.** Atomic coordinates (Å) of **+3-3** obtained at the M06-2X-D3/def2-SVP level of theory in the gas phase.

| C | -3.495025 | 1.456560 | 0.352713 | C | -3.076895 | -2.691222 | -1.771094 |
| --- | --- | --- | --- | --- | --- | --- | --- |
| C | -3.205797 | 0.103606 | 0.143240 | H | -4.485497 | 1.781295 | 0.672846 |
| C | -1.932098 | -0.279908 | -0.266348 | H | -3.969285 | -0.656396 | 0.317356 |
| C | -0.953340 | 0.696170 | -0.450882 | H | 0.052678 | 0.388428 | -0.742305 |
| C | -1.219620 | 2.029276 | -0.183143 | H | -0.780077 | -1.771369 | -1.254097 |
| C | -2.507856 | 2.430146 | 0.206664 | H | -0.146722 | -1.206966 | 1.276446 |
| C | -1.448872 | -1.709729 | -0.373502 | H | 3.564875 | 1.365969 | -2.045644 |
| C | -0.633406 | -2.113043 | 0.882725 | H | 2.832313 | 0.697166 | 2.139468 |
| C | 2.964528 | 1.535802 | -1.148263 | H | 0.774166 | 2.100470 | 1.995053 |
| C | 3.317496 | 0.897308 | 0.045943 | H | 1.492854 | 2.763757 | -2.177599 |
| C | 2.555978 | 1.162313 | 1.190561 | H | -1.950038 | 4.226094 | 0.346551 |
| C | 1.417048 | 1.957782 | 1.124783 | H | 0.035755 | -3.972346 | 0.010263 |
| C | 1.026108 | 2.488468 | -0.102385 | H | 0.857263 | -3.501183 | 1.514792 |
| C | 1.820361 | 2.330128 | -1.231953 | H | 1.442883 | -2.351084 | -1.311343 |
| O | -0.249071 | 3.012362 | -0.231003 | H | 2.954253 | -2.278040 | 1.351235 |
| O | -2.772882 | 3.729458 | 0.456142 | H | 4.760903 | -2.197007 | -0.506658 |
| C | 0.481485 | -3.121828 | 0.553608 | H | 3.595141 | -1.364737 | -1.546894 |
| C | 1.606543 | -2.509632 | -0.237328 | H | 4.755230 | -0.297571 | 1.098940 |
| C | 2.777218 | -2.119815 | 0.278469 | H | 5.265137 | 0.181777 | -0.525779 |
| C | 3.905196 | -1.500585 | -0.498403 | H | -2.165752 | -3.117092 | 1.427828 |
| C | 4.399896 | -0.151210 | 0.067091 | H | -3.876288 | -3.441523 | -1.742058 |
| O | -1.494765 | -2.584643 | 1.876965 | H | -3.509017 | -1.712499 | -2.039002 |
| O | -2.476935 | -2.657667 | -0.502958 | H | -2.345237 | -2.976737 | -2.547548 |

# **Table S10.** Atomic coordinates (Å) of **+3-4** obtained at the M06-2X-D3/def2-SVP level of theory in the gas phase.

| C | -3.495097 | 1.456479 | 0.352658 | C | -3.076786 | -2.691597 | -1.771027 |
| --- | --- | --- | --- | --- | --- | --- | --- |
| C | -3.205843 | 0.103520 | 0.143123 | H | -4.485565 | 1.781165 | 0.672844 |
| C | -1.932180 | -0.279948 | -0.266531 | H | -3.969327 | -0.656488 | 0.317217 |
| C | -0.953445 | 0.696179 | -0.451096 | H | 0.052565 | 0.388469 | -0.742582 |
| C | -1.219702 | 2.029247 | -0.183248 | H | -0.780083 | -1.771373 | -1.254211 |
| C | -2.507949 | 2.430071 | 0.206675 | H | -0.146525 | -1.206710 | 1.276174 |
| C | -1.448837 | -1.709745 | -0.373588 | H | 3.565249 | 1.366680 | -2.045434 |
| C | -0.633304 | -2.112812 | 0.882630 | H | 2.831902 | 0.696554 | 2.139321 |
| C | 2.964715 | 1.536193 | -1.148122 | H | 0.773709 | 2.099761 | 1.994951 |
| C | 3.317492 | 0.897363 | 0.045954 | H | 1.493122 | 2.764348 | -2.177363 |
| C | 2.555715 | 1.161965 | 1.190503 | H | -1.950144 | 4.226030 | 0.346477 |
| C | 1.416753 | 1.957379 | 1.124753 | H | 0.035800 | -3.972294 | 0.010421 |
| C | 1.026014 | 2.488410 | -0.102339 | H | 0.857280 | -3.500949 | 1.514893 |
| C | 1.820497 | 2.330456 | -1.231792 | H | 1.443007 | -2.351041 | -1.311307 |
| O | -0.249158 | 3.012316 | -0.231036 | H | 2.954256 | -2.278008 | 1.351318 |
| O | -2.772930 | 3.729357 | 0.456312 | H | 4.761011 | -2.196927 | -0.506525 |
| C | 0.481529 | -3.121700 | 0.553653 | H | 3.595225 | -1.364789 | -1.546815 |
| C | 1.606621 | -2.509600 | -0.237290 | H | 4.755465 | -0.297348 | 1.098838 |
| C | 2.777289 | -2.119792 | 0.278541 | H | 5.265057 | 0.181860 | -0.526002 |
| C | 3.905260 | -1.500557 | -0.498307 | H | -2.165270 | -3.117234 | 1.428096 |
| C | 4.399927 | -0.151108 | 0.067041 | H | -3.509263 | -1.713024 | -2.038933 |
| O | -1.494653 | -2.584127 | 1.877008 | H | -2.345044 | -2.976851 | -2.547493 |
| O | -2.476822 | -2.657808 | -0.502911 | H | -3.875933 | -3.442165 | -1.742004 |

# **Table S11.** Inhibitory effects of **1**–**8** against PTP1B^a^

| Compounds | PTP1B |
| --- | --- |
|  | inhibition ratio (%)^b^ |
| 1 | -9.47 ± 2.28 |
| 2 | 11.32 ± 2.03 |
| 3 | 19.07 ± 0.85 |
| 4 | 10.37 ± 2.17 |
| 5 | -0.28 ± 1.89 |
| 6 | -12.94 ± 2.98 |
| 7 | -26.62 ± 2.28 |
| 8 | -3.47 ± 1.21 |
| Suramin^c^ | 84.11 ± 0.42^d^ |

^a^Data expressed as means ± SD (n = 3). ^b^Inhibition rates than 50.0 % were screened for their IC_50_ values. ^c^Positive control.^d^At a concentration of 20 *μ*M.
